# Genome-wide data from medieval German Jews show that the Ashkenazi founder event pre-dated the 14^th^ century

**DOI:** 10.1016/j.cell.2022.11.002

**Published:** 2022-11-30

**Authors:** Shamam Waldman, Daniel Backenroth, Éadaoin Harney, Stefan Flohr, Nadia C. Neff, Gina M. Buckley, Hila Fridman, Ali Akbari, Nadin Rohland, Swapan Mallick, Iñigo Olalde, Leo Cooper, Ariel Lomes, Joshua Lipson, Jorge Cano Nistal, Jin Yu, Nir Barzilai, Inga Peter, Gil Atzmon, Harry Ostrer, Todd Lencz, Yosef E. Maruvka, Maike Lämmerhirt, Alexander Beider, Leonard V. Rutgers, Virginie Renson, Keith M. Prufer, Stephan Schiffels, Harald Ringbauer, Karin Sczech, Shai Carmi, David Reich

**Affiliations:** 1 Braun School of Public Health and Community Medicine, The Hebrew University of Jerusalem, Jerusalem 9112102, Israel; 2 Department of Human Evolutionary Biology, Harvard University, Cambridge, MA 02138, USA; 3 Department of Biology, University of Hildesheim, 31141 Hildesheim, Germany; 4 Department Anthropology, University of New Mexico, Albuquerque, NM 87131, USA; 5 Center for Stable Isotopes, University of New Mexico, Albuquerque, NM 87106, USA; 6 Research Reactor Center, University of Missouri, Columbia, MO 65211, USA; 7 Medical Genetics Institute, Shaare Zedek Medical Center, Jerusalem 9103102, Israel; 8 Faculty of Medicine, The Hebrew University of Jerusalem, Jerusalem 9112001, Israel; 9 Department of Genetics, Harvard Medical School, Boston, MA 02115, USA; 10 Broad Institute of MIT and Harvard, Cambridge, MA 02142, USA; 11 Howard Hughes Medical Institute, Harvard Medical School, Boston, MA 02115, USA; 12 Department of Zoology and Animal Cell Biology, University of the Basque Country UPV/EHU, 01006 Vitoria-Gasteiz, Spain; 13 Ikerbasque—Basque Foundation of Science, 48009 Bilbao, Spain; 14 Independent Scholar, Kalamazoo, MI 49008, USA; 15 Independent Scholar, New York, NY 10027, USA; 16 Faculty of Biotechnology and Food Engineering, Technion – Israel Institute of Technology, Haifa 3200003, Israel; 17 Department of Psychiatry, Division of Research, The Zucker Hillside Hospital Division of Northwell Health, Glen Oaks, NY 11004, USA; 18 Departments of Medicine and Genetics, Albert Einstein College of Medicine, Bronx, NY 10461, USA; 19 Department of Genetics and Genomic Sciences, Icahn School of Medicine at Mount Sinai, New York, NY 10029, USA; 20 Faculty of Natural Sciences, University of Haifa, Haifa 3498838, Israel; 21 Department of Pathology, Albert Einstein College of Medicine, Bronx, NY 10461, USA; 22 Departments of Psychiatry and Molecular Medicine, Zucker School of Medicine at Hofstra/Northwell, Hempstead, NY 11550, USA; 23 Institute for Behavioral Science, The Feinstein Institutes for Medical Research, Manhasset, NY 11030, USA; 24 Department of Medieval History, University of Erfurt, 99089 Erfurt, Germany; 25 Independent Scholar, 92370 Chaville, France; 26 Department of History and Art History, Utrecht University, Utrecht 3512 BS, the Netherlands; 27 Department of Archaeogenetics, Max Planck Institute for Evolutionary Anthropology, 04103 Leipzig, Germany; 28 World Heritage Coordinator, City of Erfurt, 99084 Erfurt, Germany; 29 Lead contact

## Abstract

We report genome-wide data from 33 Ashkenazi Jews (AJ), dated to the 14^th^ century, obtained following a salvage excavation at the medieval Jewish cemetery of Erfurt, Germany. The Erfurt individuals are genetically similar to modern AJ, but they show more variability in Eastern European-related ancestry than modern AJ. A third of the Erfurt individuals carried a mitochondrial lineage common in modern AJ and eight carried pathogenic variants known to affect AJ today. These observations, together with high levels of runs of homozygosity, suggest that the Erfurt community had already experienced the major reduction in size that affected modern AJ. The Erfurt bottleneck was more severe, implying substructure in medieval AJ. Overall, our results suggest that the AJ founder event and the acquisition of the main sources of ancestry pre-dated the 14^th^ century and highlight late medieval genetic heterogeneity no longer present in modern AJ.

## In brief

Genome-wide data was generated for 33 individuals whose skeletons were rescued from a late medieval Jewish cemetery in Erfurt, Germany. Medieval German Jews had similar genetic ancestry to modern Ashkenazi Jews but were more genetically heterogeneous, likely divided into two or more distinct groups. The medieval Jews showed evidence of an extreme founder event, including high levels of runs of homozygosity and distinctive genetic variants shared with modern Ashkenazi Jews.

## Graphical abstract

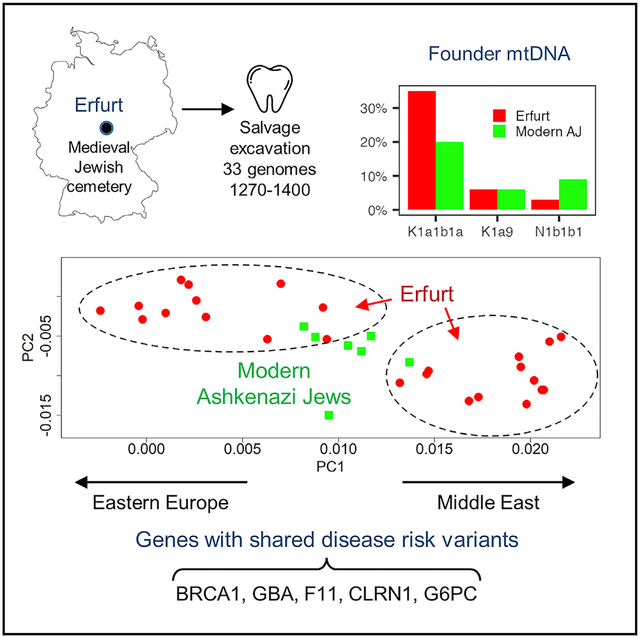

## INTRODUCTION

Ashkenazi Jews (AJ) emerged as a distinctive ethno-religious cultural group in the Rhineland in the 10^th^ century (Frishman, 2008; Gladstein and Hammer, 2016). Since then, the AJ population expanded substantially, both geographically, first to Eastern Europe and recently beyond Europe, and in number, reaching about 10 million today (DellaPergola, 2015; Motulsky, 1995). Starting from the early days of human genetics, dozens of pathogenic recessive variants were identified in AJ (Charrow, 2004; Goodman, 1979; Ostrer, 2012), leading to the development of successful pre-conception screening programs (Gross et al., 2008; Kaback et al., 1993). A large fraction of these variants are extremely rare outside AJ and appear on the background of long shared haplotypes (e.g., Frisch et al., 2004; Hamel et al., 2011; Laitman et al., 2013; Raskin et al., 2011), implying that AJ descend from a small set of ancestral founders (Diamond, 1994; Ostrer and Skorecki, 2013; Risch et al., 2003; Slatkin, 2004). The Ashkenazi “founder event” is also evident in four mitochondrial lineages carried by 40% of AJ (Behar et al., 2006; Costa et al., 2013). More recently, studies found high rates of identical-by-descent (IBD) sharing in AJ, that is, nearly identical long haplotypes present in unrelated individuals, a hallmark of founder populations (Atzmon et al., 2010; Carmi et al., 2014a; Gusev et al., 2012; Henn et al., 2012). Quantitative modeling suggested that AJ experienced a sharp reduction in size (a “bottleneck”) in the late Middle Ages and that the (effective) number of founders was in the hundreds (Carmi et al., 2014a; Granot-Hershkovitz et al., 2018; Palamara et al., 2012; Santiago et al., 2020; Tournebize et al., 2022).

The origins of early AJ, as well as the history of admixture events that have shaped their gene pool, are subject to debate (Data S1, section 1). Genetic evidence supports a mixed Middle Eastern (ME) and European (EU) ancestry in AJ. This is based on uniparental markers with origins in either region (Behar et al., 2006, 2017; Costa et al., 2013; Hammer et al., 2000, 2009; Nebel et al., 2001), as well as autosomal studies showing that AJ have ancestry intermediate between ME and EU populations (Atzmon et al., 2010; Behar et al., 2010, 2013; Bray et al., 2010; Carmi et al., 2014a; Granot-Hershkovitz et al., 2018; Guha et al., 2012; Kopelman et al., 2020). These and other autosomal studies also showed that individuals with AJ ancestry are genetically distinguishable from those of other ancestries. Recent modeling suggested that most of the European ancestry in AJ is consistent with Southern European-related sources, and estimated the total proportion of European ancestry in AJ as 50%–70% (Carmi et al., 2014a; Xue et al., 2017; Yardumian and Schurr, 2019). The AJ population is overall highly genetically homogeneous, with no major ancestry differences based on present-day country of residence (Guha et al., 2012; Gusev et al., 2012; Kopelman et al., 2020). However, there are subtle average differences in ancestry between AJ with origins in Eastern vs. Western Europe (Behar et al., 2013; Gladstein and Hammer, 2019; Granot-Hershkovitz et al., 2018) (Data S1, section 1).

Despite the recent progress, open questions remain, including the localization of the founder event, or events, in time and space and the sources and times of the admixture events. Studying the genomes of individuals who lived closer to the time of AJ formation has the potential to shed light on these questions. We present here a DNA study of historical Jews, focusing on AJ from 14^th^-century Erfurt, Germany. The medieval Erfurt Jewish community existed between the late 11^th^ century to 1454, with a short gap following a 1349 massacre (Weigelt, 2016). We report genome-wide data from 33 individuals whose skeletons were extracted in a salvage excavation. Our results demonstrate that Erfurt Ashkenazi Jews (EAJ) are genetically similar to modern Ashkenazi Jews (MAJ), implying little gene flow into the AJ gene pool since the 14^th^ century. Further analysis demonstrates that EAJ were more genetically heterogeneous than MAJ, with multiple lines of evidence supporting the presence of two subgroups, one of which had higher Eastern European affinity compared to MAJ. The EAJ population shows strong evidence of a recent bottleneck shared with the bottleneck that affected MAJ, as alleles that are highly enriched in MAJ—including mitochondrial lineages and pathogenic variants—are also common in EAJ.

## RESULTS

### Historical and archaeological context, community engagement, and sample collection

**Figure 1.**
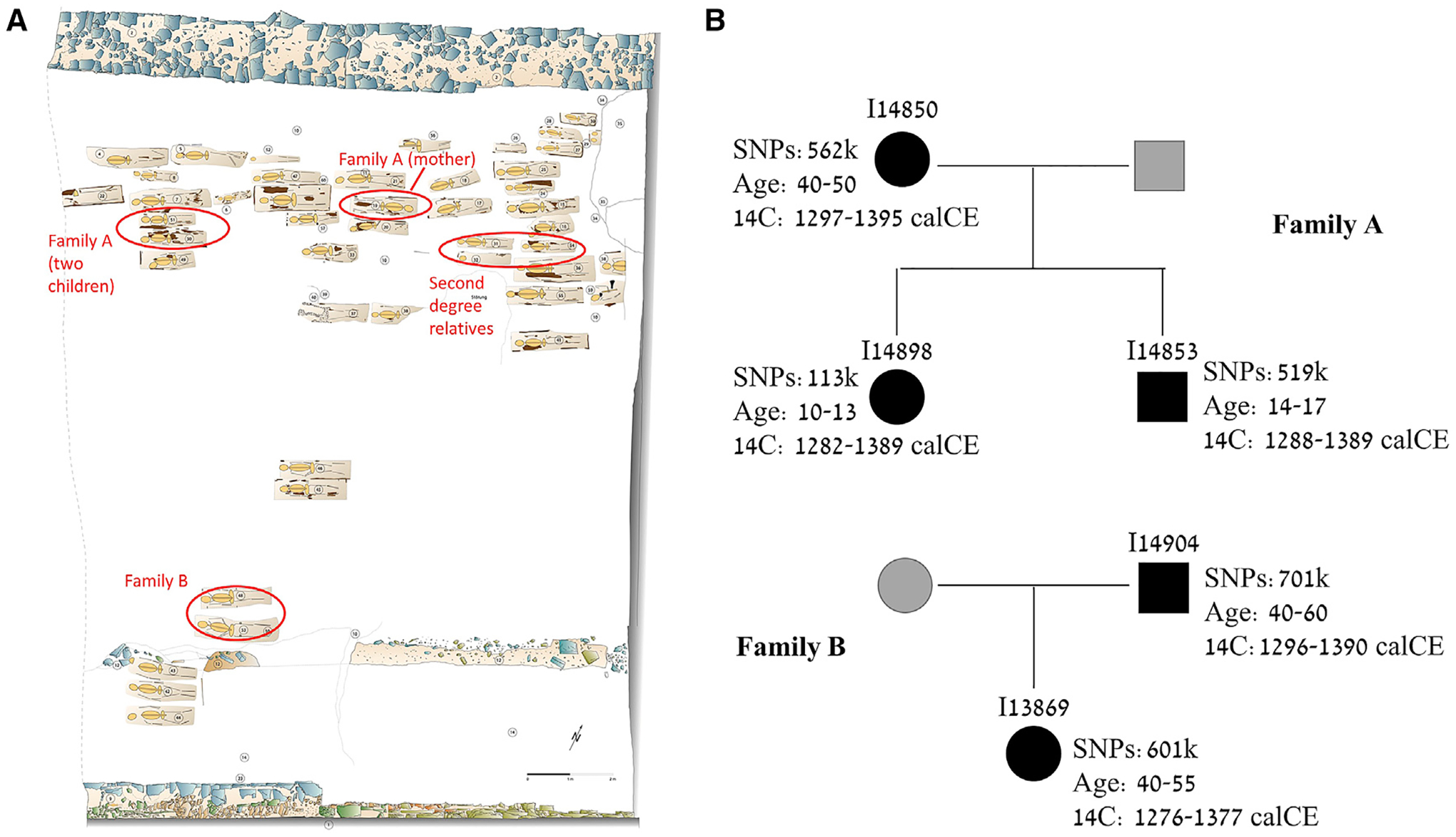
The medieval Jewish cemetery at Erfurt (A) The layout of the cemetery. The inner city wall and the outer city wall are at the bottom and top of the map, respectively. Family members are marked in red ellipses (see next). (B) The pedigrees of the two families identified based on first degree relationships. Black symbols represent individuals with DNA; gray symbols represent inferred family members. Circles: females; squares: males. For each individual, we indicate the ID, the number of genotyped SNPs, the estimated age at death, and the ^14^C date (95.4% probability intervals). See also Figure S1.

The first Jewish community of Erfurt (pre-1349) was the oldest in Thuringia, and its cemetery also served nearby towns (Lämmerhirt, 2015; Toch, 1992). During the 1349 pogrom, most Jews of Erfurt and nearby communities were murdered or expelled (Weigelt, 2016). Jews returned to Erfurt in 1354 to form the second community (Lämmerhirt, 2016), which was one of the largest in Germany (Toch, 1992) (Data S1, section 2). The individuals we studied were buried in the south-western part of the medieval Jewish cemetery of Erfurt, which underwent salvage excavations in 2013 (STAR Methods and Data S1, section 3). The layout of the cemetery is shown in Figure 1A (see also Data S1, section 3).

Archaeological evidence tentatively suggests that the site was used by the second community (Data S1, section 3). Radiocarbon dating of ten individuals demonstrated that all lived between about 1270 and 1400 CE (STAR Methods and Data S2, Tables 1 and 3), but data were not informative on whether the site was used by the first or second community, due to a wiggle in the ^14^C calibration curve around the mid-14^th^ century (Figure S1 and Data S1, section 3). The estimated ages at death (STAR Methods) ranged between 5 and 60 years old, with 14/33 (42%) estimated to be younger than 20 (Data S2, Table 1). The cause of death could be determined only for I14904, who was killed by several blows to the head by a sharp object.

Jewish rabbinical law, which was followed by EAJ (Data S1, section 2) prohibits exhumation of Jews for most purposes, and also proscribes disturbing the dead. Rabbinical discussions on testing ancient DNA from Jewish individuals have only recently appeared (Litke, 2021; Steinberg, 2019; Vigoda, 2017) and there is no centralized authority for establishing Jewish rabbinical guidance. As part of this study, we engaged with rabbinical authorities who reviewed our proposed research plan and approved the project under the conditions that only detached teeth are used, and that the analysis is performed only on already-excavated individuals. The study was then approved by the Jewish community of Thuringia, Germany. Following these guidelines, we sampled teeth from 38 skeletal remains.

### DNA sequencing

We followed existing protocols for DNA extraction, library preparation, and enrichment for about 1.24 million single nucleotide polymorphisms (SNPs). We then sequenced the enriched and non-enriched libraries and used multiple metrics to demonstrate very low levels of contamination (STAR Methods and Data S2, Table 2). We obtained genome-wide data passing quality control for 33 individuals: 19 females and 14 males. The median proportion of human sequences was 0.03 (range 0.0003–0.67; Data S2, Table 2), and the median coverage on autosomal targets was 0.45× (range 0.01–2.48×). The median number of SNPs covered by at least one sequence was 383k (range 11–842k). Children had significantly lower coverage than adults (p = 6.7·10^−7^; Data S1, section 4). We found no traces of *Yersinia pestis* bacteria in the DNA sequences (STAR Methods, Data S1, section 4, and Data S2, Table 4).

We identified two families based on first-degree relationships (STAR Methods and Figure S1): family A, with a mother, a son, and a daughter; and family B, with a father (the one killed by strokes to the head) and a daughter (Figure 1B). The two children of family A were buried next to each other, as were the two members of family B (Figure 1A). The mother of family A was buried three rows away from her children, in an orientation opposite to all other burials (Figure 1A). We also identified three distantly related individuals, likely second-degree relatives, who were all buried next to each other (Figure 1A). Two of them were the only ones in our sample to carry the U5a1a2a mtDNA lineage (Data S2, Table 1). However, the data for these three individuals were also consistent with a first-degree or no relationship (Figure S1), likely due to low coverage (13k, 15k, and 38k SNPs).

### Ancestry estimation and medieval population structure

To analyze the ancestry of the EAJ individuals, we represented their genomes as “pseudo-haploids” using a single random sequence for each covered SNP. We merged EAJ with modern genomes from the Human Origins dataset (about 593k autosomal SNPs, all also enriched in EAJ), which included seven AJ and 86 other Jews. We projected the EAJ individuals on principal components learned from West Eurasian individuals of the Human Origins dataset (n = 994; STAR Methods). Eight EAJ had (post-merging) coverage of fewer than 50k SNPs, which did not allow reliable projection (Data S1, section 5). We designated these individuals as “low-coverage” and excluded them from the principal components analysis (PCA).

**Figure 2.**
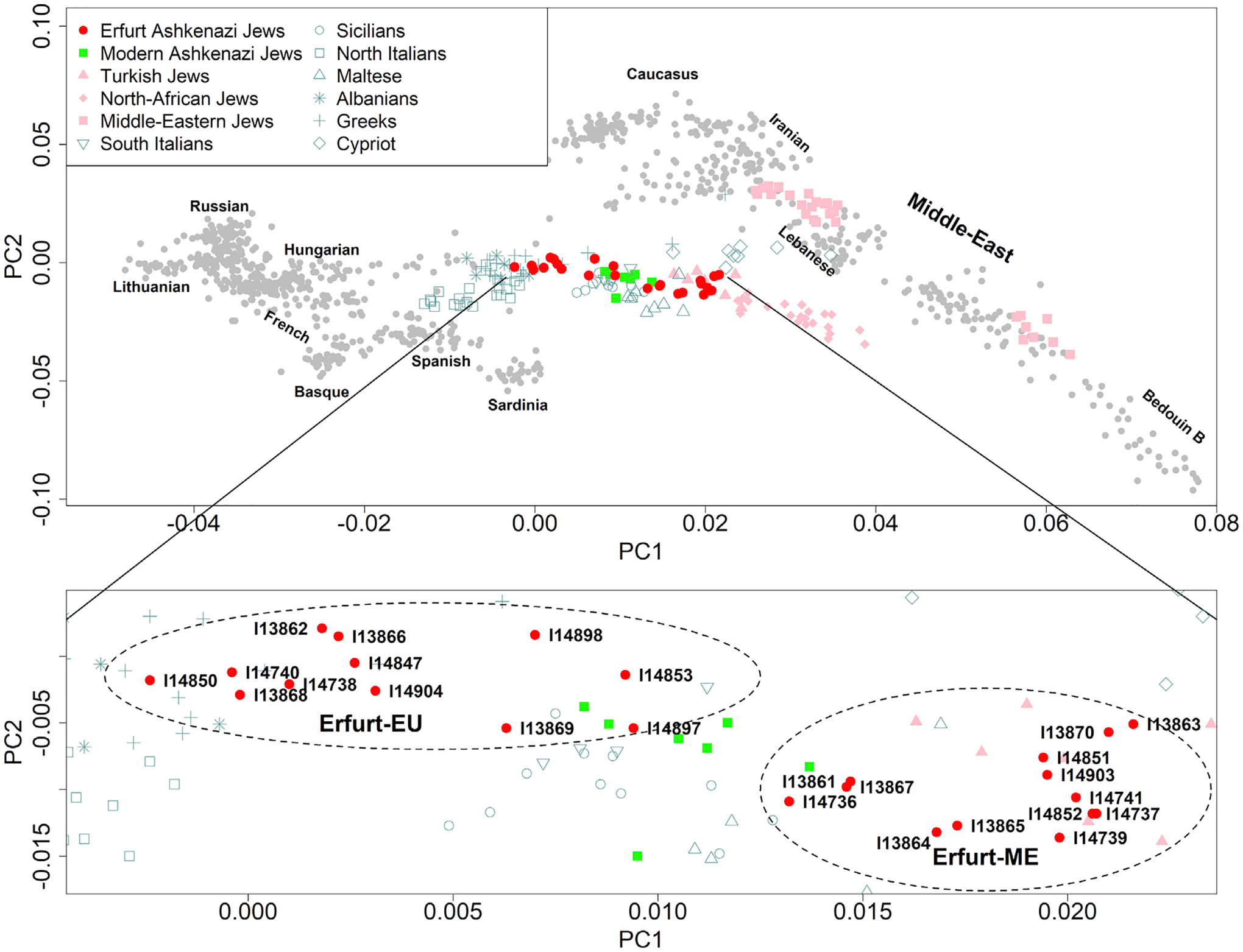
Principal components analysis We learned the principal components (PCs) using West Eurasian populations (Lazaridis et al., 2014) and projected the Erfurt individuals (filled red circles) onto the inferred axes. Modern Ashkenazi Jews (green squares), Jews of non-Ashkenazi origin (pink shapes), and Mediterranean populations (teal shapes) are highlighted. The inset zooms in on the region that contains AJ individuals. See also Figure S2.

In the PCA plot, EAJ individuals overlapped with MAJ, as well as with other European Mediterranean populations. Interestingly, EAJ had more variability along the European-Middle Eastern cline than MAJ (Figure 2). Higher variability in EAJ relative to MAJ was also observed when projecting a much larger MAJ sample (Figure S2) as well as in an ADMIXTURE analysis (Alexander et al., 2009) (Data S1, section 5). We used *K*-means to cluster the EAJ individuals based on their PC1 and PC2 coordinates into two groups. One group, which we call “Erfurt-EU”, falls closer to individuals from European populations, while the other, which we call “Erfurt-ME”, is closer to Middle Eastern populations (Figure 2, inset).

Multiple lines of evidence provided statistical support for the dichotomization of EAJ as compared to a continuous gradient. A “gap statistic” analysis suggested that the optimal number of clusters is two, and another method showed that the clusters are significantly different (Data S1, section 6). To investigate the question from a population genetic perspective, we simulated two scenarios, either where all individuals had the same demographic history, or where one subgroup had an additional admixture event in Europe (Data S1, section 6). Summary statistics from the single group simulations (but not from the two-group simulations) were significantly different from those of EAJ, again supporting the presence of two distinct subgroups (Data S1, section 6). We also confirmed that clustering the EAJ individuals into two groups using two additional methods yielded identical clusters (Data S1, section 6). These results support the presence of at least two genetically distinct (even if possibly overlapping) groups in EAJ.

To further characterize the two Erfurt subgroups, we analyzed them separately and tested how they relate to MAJ of Eastern European or Western European origin (Data S1, section 5). Western MAJ almost entirely overlapped with Erfurt-ME in PCA. Eastern MAJ were intermediate between the two EAJ groups, and closer to Erfurt-ME (Figure S2). The Erfurt-ME group also overlapped with present-day Turkish (Sephardi) Jews (Figure 2, inset).

We used f_4_-statistics to test for evidence of gene flow between EAJ, MAJ, and other EU and ME populations (STAR Methods). We first ran tests of the form f_4_(MAJ, EAJ; X, chimp), where X is any West Eurasian population. The results showed increasing *Z* scores, and hence increased affinity with MAJ as opposed to EAJ, as X changed from Eastern European to Central/Western European, Mediterranean, and Middle Eastern (Figure S3A). The same trend was observed for tests f_4_(MAJ, Erfurt-EU; X, chimp), but the trend was opposite for tests f_4_(MAJ, Erfurt-ME; X, chimp) (Figure S3A). These results suggest, in agreement with the PCA, that Erfurt-EU have more EU ancestry—particularly Eastern European—than Erfurt-ME or MAJ. In agreement, tests of the form f_4_(Erfurt-EU, Erfurt-ME; X, chimp) showed increasing affinity with Erfurt-ME as X changed from Eastern European to Central/Western European, Mediterranean, and Middle Eastern (Figure S3B). Finally, tests f_4_(MAJ, X; EAJ, chimp), where X is any Jewish non-Ashkenazi population, were positive and very large for all X, suggesting that EAJ (including both subgroups) are closer to MAJ than to any other Jewish group (Figure S3C).

**Figure 3.**
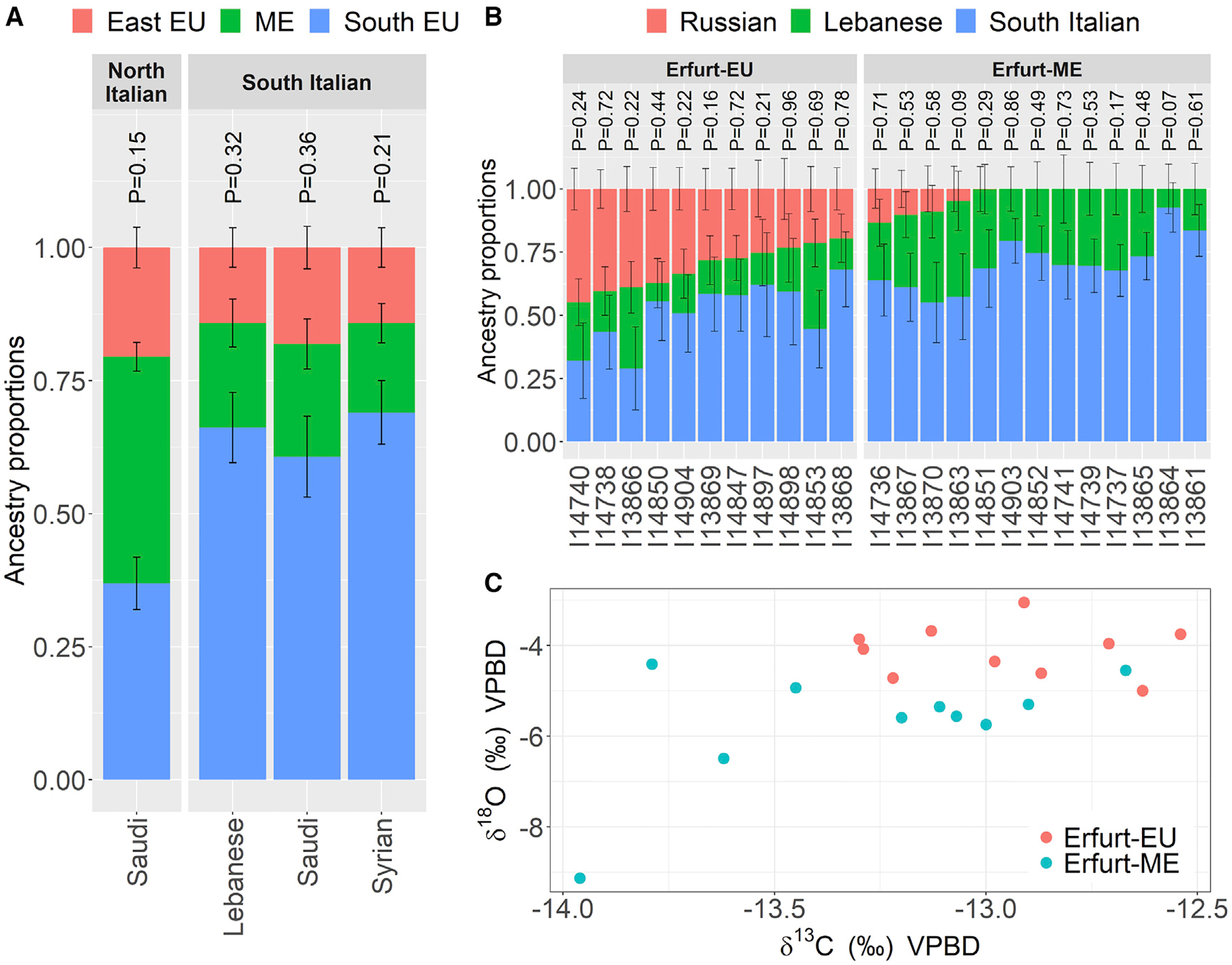
Models for the ancestry of Erfurt Ashkenazi Jews (A) Each qpAdm model for the ancestry of Erfurt Jews includes a Middle Eastern, a Southern European, and an Eastern European (Russians) source. The Southern European source was either South or North Italians, as indicated at the top of each panel. The Middle Eastern source is indicated in the x axis labels. Only models with qpAdm p value >0.05 in the main analysis and in the robustness tests are shown (Table S3). Error bars represent one standard error in each direction. qpAdm p values are presented above each model. (B) The ancestry of single Erfurt individuals, labeled by their IDs. We used qpAdm with Russian, Lebanese, and South Italian sources. The individuals are labeled by their Erfurt subgroup (EU/ME). qpAdm p values are shown for each individual. Results are not shown for low-coverage individuals (<50k SNPs), and for an additional individual who could not be modeled using these sources (p < 0.05). (C) A plot of δ^13^C_enamel_ and δ^18^O_enamel_ stable isotope ratios for a subset of 20 Erfurt individuals with >200k SNPs. The Erfurt subgroup affiliation (EU/ME) is color-coded (legend). See also Figure S3.

The increased affinity of Erfurt-EU with Eastern European populations may be related to the historically recorded migration of families from Bohemia, Moravia, and Silesia into the second Erfurt community (Data S1, section 2) (Lämmerhirt, 2019). To test whether some EAJ individuals had evidence of being migrants, we performed a stable isotope analysis on bioapatite derived from dental enamel (STAR Methods, Data S2, Tables 1 and 5). The δ^13^C_enamel_ and δ^18^O_enamel_ values are plotted for individuals with high coverage in Figure 3C, showing distinct distributions of stable isotope ratios between the two genetic groups. The differences were significant for δ^18^O (p = 0.0005; two-tailed Wilcoxon test), suggesting average differences in water sources during childhood between Erfurt-EU and Erfurt-ME. We found no correlation between the locations of the graves in the cemetery and the group affiliation (Mantel test p = 0.46) or PC1 and PC2 coordinates (Mantel test p = 0.41), showing that even though these groups were genetically distinctive, they show no evidence of being culturally or temporally segregated.

A qpWave analysis showed that EAJ and MAJ are consistent with forming a clade with respect to non-Jewish Europeans (p = 0.15; Table S1 and STAR Methods). This genetic similarity between EAJ and MAJ, despite living 600–700 years apart, suggests a high degree of endogamy over the period. Using simulations (STAR Methods), we inferred that any hypothetical admixture event between AJ and Eastern Europeans over the past ≈ 20 generations must have been limited to replacing at most 2%–4% of the total AJ gene pool (corresponding to at most 0.2% replacement per generation; Table S2 and STAR Methods). The same qpWave analysis with Erfurt-EU or Erfurt-ME had lower p values, particularly for Erfurt-EU, suggesting that each of these groups alone does not fully represent the entire modern AJ gene pool. When replacing EAJ or MAJ with (modern) Turkish Jews, South Italians, and Germans, all p values were under 0.05 (suggesting inconsistency with the given pair of populations being a clade), with the highest p value observed when comparing Erfurt-ME and Turkish Jews (p = 0.01; Table S1). We repeated the qpWave analysis to test whether pairs of populations form a clade with respect to non-Jewish Middle Eastern populations (Table S1). The p values were >0.05 for tests comparing MAJ and EAJ/Erfurt subgroups and either of these populations and Turkish Jews, suggesting that these populations have similar sources of Middle Eastern ancestry.

### Quantitative ancestry modeling

We used qpAdm to test quantitative models for the ancestral sources of EAJ (STAR Methods). Based on the PCA above and previous modeling (Xue et al., 2017), we considered a model where EAJ is a mixture of the following sources: Southern European (South Italians or North Italians), Middle Eastern (Druze, Egyptians, Bedouins, Palestinians, Lebanese, Jordanians, Syrians, or Saudis), and Eastern European (Russians). We used modern populations as sources, as modeling with ancient sources was unsuccessful (Data S1, section 7). Multiple models with South-Italians were plausible (p>0.05; Table S3), which would be consistent with historical models pointing to the Italian peninsula as the source for the AJ population (Data S1, section 16; though see below for alternatives and caveats). The mean admixture proportions (over all of our plausible models; Table S3) were 65% South Italy, 19% ME, and 16% East-EU (Figure 3A). We validated that our results did not qualitatively change when using only transversions vs. all SNPs, a different outgroup population, or fewer SNPs (Table S3; Data S1, section 7). Estimates of the admixture time were unreliable in our setting (Data S1, section 8).

Models with other sources, in particular Mediterranean, could also fit the EAJ data (Data S1, section 7). A model with North Italians as a source (Table S3) had ancestry proportions 37% North Italy, 43% ME, and 20% East-EU (Figure 3A). Models with Greek as a source had average ancestry proportions 51% Greek, 32% ME, and 17% East-EU (Table S3). Models with Spanish or North African sources (in addition to ME and East-EU) were not plausible. A model with all Levant populations merged together as the ME source fit the EAJ data (p = 0.07), with ancestry proportions 65% South Italy, 19% Levant, and 16% East-EU. A model with all Mediterranean populations merged as a single source (Middle Eastern, Greek, and Italian, with East-EU as the other source) fit the data (p = 0.11) with ancestry proportions 89% Mediterranean and 11% East-EU. Models with a Western European source (Germans) instead of Russians were not plausible. There was also no support for a minor ancestry component from East Asians.

We next used qpAdm to study the relations between EAJ, MAJ, and other Jewish groups (Data S1, section 7). Erfurt-ME could be modeled with Turkish (Sephardi) Jews (97% admixture proportion) and Germans (3%). Erfurt-EU could be modeled with Erfurt-ME (69%) and Russians (31%). Using a model with South Italian, Lebanese, and Russian sources for each EAJ individual (Figure 3B), we found striking variability in the Eastern European component, which was on average 33% in Erfurt-EU individuals but was not inferred in 9 of 13 Erfurt-ME individuals. Similar variability was observed using a North Italian source (Data S1, section 7). MAJ could be modeled with Erfurt-ME (87%) and Russians (13%), or Erfurt-ME (86%) and Germans (14%). MAJ could also be modeled as having 60% ancestry from Erfurt-ME and 40% from Erfurt-EU (Data S1, section 7).

Taken together, our results suggest that Erfurt-ME is a population genetically close to Sephardi Jews that has left nearly unadmixed descendants in modern AJ of Western European origin, while Erfurt-EU has experienced additional Eastern European-related admixture. The majority of AJ today likely formed as a nearly even mixture of populations represented by the two Erfurt groups. Linguistic, cultural, and onomastic studies have found differences between AJ from Western Europe, representing the early Rhineland communities, and AJ from Central and Eastern Europe (Data S1, section 16). Our results suggest the hypothesis that Erfurt-ME and Erfurt-EU may be related to the Western and Central/Eastern AJ groups, respectively. The non-genetic differences nearly vanished by the 16^th^ century (Beider, 2015), consistent with the lack of major genetic structure in modern AJ.

We caution that the specific identity of the source populations that we inferred, as well as the admixture proportions, should not be considered precise. This is due to the multiple Southern European populations that fit the EAJ data, as well as our reliance on modern populations as a proxy of the true ancestral sources. The levels of Middle Eastern ancestry in Italy were historically variable (Aneli et al., 2021; Antonio et al., 2019; De Angelis et al., 2021; Posth et al., 2021; Raveane et al., 2019), and Middle Eastern populations have also experienced demographic changes in the past two millennia, particularly African admixture (Moorjani et al., 2011) (Data S1, section 16). Under the extensive set of models we studied, the ME ancestry in EAJ is estimated in the range 19%–43% and the Mediterranean European ancestry in the range 37%–65%. However, the true ancestry proportions could be higher or lower than implied by these ranges (Data S1, section 16). Our results therefore should only be interpreted to suggest that AJ ancestral sources have links to populations living in Mediterranean Europe and the Middle East today.

### Evidence for a founder event in the history of Erfurt Jews

Previous analyses of identical-by-descent (IBD) haplotypes (Carmi et al., 2014a; Granot-Hershkovitz et al., 2018; Palamara et al., 2012), mtDNA lineages (Behar et al., 2006), and pathogenic variants (Risch et al., 2003; Slatkin, 2004) suggested that AJ experienced a medieval founder event (a bottleneck). However, the details of the event are yet to be fully resolved. Here, we used three sources of information — mtDNA lineages, runs of homozygosity, and MAJ-enriched variants — to test whether the EAJ population was shaped by the same founder event.

We list the mtDNA lineages of EAJ in Data S2, Table 1 and report the number of carriers of the four most common Ashkenazi lineages (Behar et al., 2006) in Table S4. Remarkably, among 31 unrelated EAJ, 11 (35%) carried the K1a1b1a lineage, which is nearly absent in individuals of non-Jewish ancestry (Behar et al., 2006). This is greater than the 20% frequency in MAJ (p = 0.04; two-tailed binomial test; Table S4). All 11 carriers had identical sequences except for a single C/T polymorphism at position 16223 (C count: 3/11; Data S1, section 9). A joint Bayesian analysis (Bouckaert et al., 2019) of MAJ and EAJ K1a1b1a carriers (accounting for the known date of the EAJ individuals; STAR Methods) suggested a median posterior time to the most recent common ancestor about 1500 years ago, slightly earlier than previous point estimates (Behar et al., 2006; Costa et al., 2013), but with very high uncertainty (95% highest posterior density interval: 650–6700; Data S1, section 9).

The frequency of K1a1b1a in Erfurt-ME (7/13 = 54%) was higher than the frequency in Erfurt-EU (2/10 = 20%). The difference was not statistically significant, given the small sample size (p = 0.20; two-tailed Fisher’s Exact test); however, a higher prevalence of K1a1b1a in Erfurt-ME is concordant with its higher frequency in AJ of Western European origin (Behar et al., 2006; Costa et al., 2013). Among the other AJ founder lineages, two EAJ individuals carried the K1a9 lineage, one carried N1b1b1 (N1b2), and none carried K2a2a1 (Table S4). Overall, the mtDNA results provide evidence that EAJ were affected by the same bottleneck that affected MAJ.

**Figure 4.**
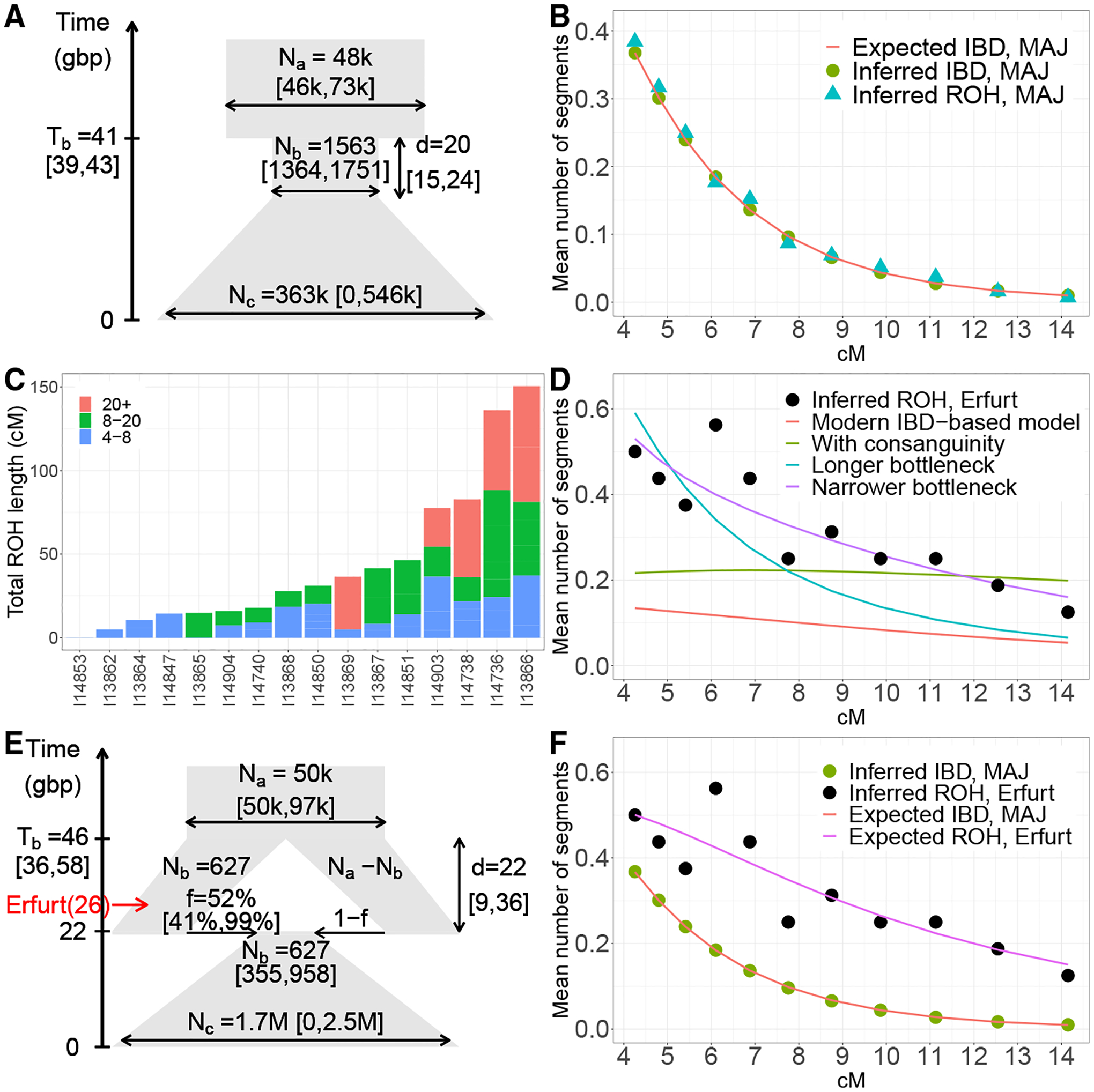
Models for AJ demographic history based on ancient and modern haplotypes (A) A single-population model for the demographic history of AJ, inferred based on modern IBD sharing (Table S5, model [A]). In the diagram, the y axis represents the time in generations before present (gbp) and the width is (schematically) proportional to the effective population size. The 95% confidence intervals (CI) were computed using bootstrapping and are indicated near each parameter. (B) The mean number of IBD and ROH segments (per pair of haploid autosomal genomes) in modern AJ vs. segment length (11 bins between 4 and 15 cM). Each symbol (circles for IBD, triangles for ROH) is placed at the middle of its corresponding bin. The red line shows the expected number of segments per bin based on the demographic model of (A). (C) The total length of ROH segments in 16 EAJ individuals with >400k SNPs. The bars are colored proportionally to the contribution of segments of different lengths (legend). (D) ROH counts in EAJ (circles) and the expected number based on various models (lines). The model inferred using modern IBD (panel [A]) is in red. The same model but allowing consanguinity in EAJ (Table S5, model [D]) is in green. Both models fit poorly to short ROH segments. A model similar to (A) but with either a narrower or a longer bottleneck (Table S5, models [E] and [F], respectively) are in purple and teal, respectively. (E) A two-population model inferred jointly using IBD in MAJ and ROH in EAJ (Table S5, model [H]). According to the model, the ancestral population split *T*_*b*_ = 46 generations ago into one population of effective size *N*_*b*_ = 627 (representing Erfurt, indicated in red) and another of size *N*_*a*_ − *N*_*b*_. At the end of the bottleneck, the two populations merged with proportions *f* = 52% and 1 − *f*, respectively, and expanded exponentially as in the single-population model. The time of sampling of the Erfurt population is shown at 26 generations ago (assuming 25 years per generation). (F) Counts of IBD segments in MAJ (green circles; same data as in [B]) and ROH in EAJ (black circles; same data as in [D]), and the expectations (lines) based on the two-population model of (E) (MAJ, red; EAJ, pink). See also Figures S4 and S5.

Runs of homozygosity (ROH) appear in individuals whose parents are genetically related and are also expected in founder populations (Severson et al., 2019). We detected ROH in EAJ using hapROH (Ringbauer et al., 2021) (STAR Methods and Figure S4). We focused on 16 individuals covered in at least 400k SNPs (Data S1, section 10). The EAJ individuals had substantially higher levels of ROH compared to most other individuals from the historical period (Figure S4), with an average of 44 cM per individual in segments greater than 4 cM (30 cM in segments >8 cM and 14 cM in segments >20 cM; Figure 4C). These results provide additional support to the hypothesis that EAJ were affected by a strong bottleneck.

We next considered variants that are specific (or nearly specific) to MAJ and are also present in EAJ. We define AJ *founder alleles* as minor alleles (not necessarily disease causing) in SNPs targeted by our in-solution enrichment that have frequency >0.5% in MAJ, <0.01% in Europeans (both using gnomAD [Karczewski et al., 2020]), and ≲ 1% in the Middle East (STAR Methods). Overall, we identified 216 AJ founder alleles. Among these, 15 were present in at least one EAJ individual (Data S2, Table 1). To determine whether this number is expected under the scenario where EAJ post-dated the bottleneck, we used MAJ allele frequencies and ran binomial simulations to estimate the number of observed alleles given the EAJ sample size and per-individual coverage (STAR Methods). The [2.5,97.5]-percentiles of the simulated allele counts in the EAJ-like individuals were [14,32] (Figure S5C). (These expected allele counts are likely somewhat overestimated, due to the conditioning on alleles whose MAJ frequency was above a cutoff [Data S1, section 11]). The presence of 15 founder alleles in EAJ is thus within the range expected if EAJ have already experienced the AJ bottleneck (Data S1, section 11).

### Inferring a demographic model of the founder event

To quantitatively estimate the AJ bottleneck parameters, we inferred demographic models based on IBD sharing (Palamara et al., 2012). We started with a model of an ancestral population of a constant (effective) size that experienced a bottleneck of effective size *N*_*b*_ starting *T*_*b*_ generations ago and lasting *d* generations, followed by an exponential expansion (Figure 4A). We used whole-genome sequencing data for *n* = 637 MAJ individuals (Carmi et al., 2014a; Lencz et al., 2018) and inferred IBD segments using IBDseq (STAR Methods). We then estimated the bottleneck parameters using a maximum likelihood approach (STAR Methods): *N*_*b*_ = 1563 (diploid individuals; 95% confidence interval (CI): [1364–1751]), *T*_*b*_ = 41 (generations; 95% CI: [39–43]), and *d* = 20 (generations; 95% CI: [15–24]; see Figure 4A and Table S5). The predicted counts of IBD segments (based on the model of Figure 4A) provide good fit to the counts from the MAJ data (Figure 4B). Assuming 25 years per generation, our model places the onset of the bottleneck about 1000 years ago, at the formation of the early AJ communities (Data S1, section 16). We inferred a very similar model using ROH in the same MAJ individuals (Data S1, section 12) with good fit to the empirical data (Figure 4B).

We next sought to determine whether our inferred demographic model provides a good fit to the ancient EAJ data (after accounting for the ≈650-year difference; STAR Methods), which would be expected given the genetic similarity between MAJ and EAJ. However, ROH counts in EAJ exceeded the expectation based on the model (Figure 4A), in particular for short and intermediate segments (Figure 4D), indicating that the Erfurt bottleneck must have been more severe. Given previous simulation results (Ringbauer et al., 2021) and further exploration here (Figure S4), the gap is unlikely to be due to false positive ROH calls. Modeling consanguinity in EAJ also did not explain the excess of short ROH (Figure 4D and Data S1, section 12). To fit the ancient data, the Erfurt bottleneck must have been either 3.0-fold narrower or 2.4-fold longer compared to the expectation based on modern IBD (Figure 4D, Table S5, and Data S1, section 12). However, these bottleneck parameters did not fit the modern data (Figure S5A). The best fit model to both modern IBD and ancient ROH data also did not provide good overall fit (Figure S5B, Table S5, and Data S1, section 12). Taken together, these results suggest that the single-population (and single-bottleneck) model of Figure 4A does not fully describe the AJ demographic history.

Given that the Erfurt population only represents a single AJ medieval site, we hypothesized that a missing component in our model is medieval substructure. We therefore considered a model in which the AJ population split at the onset of the bottleneck into two groups experiencing different bottleneck intensities, one of them represented by EAJ (Figure 4E) and the other unsampled. We showed by simulations that our method can infer the model parameters even in the presence of extreme imbalance between the amount of modern and ancient data (Data S1, section 13). In the best fit model (Figure 4E and Table S5), the bottleneck started *T*_*b*_ = 46 generations ago (95% CI: [36,58]) and lasted *d* = 22 generations (95% CI: [9,36]). The group represented by EAJ experienced a narrow bottleneck of size *N*_*b*_ = 627 (95% CI: [355, 958]) and contributed 52% (95% CI: [41,99]%) to the MAJ gene pool. The remaining contribution came from a second group that did not experience the initial bottleneck but contracted only at the end of the bottleneck, when merging with the other AJ group (Figure 4E). This model fits well both modern and ancient data (Figure 4F). We used parametric bootstrapping to demonstrate that the favorable fit is not due to over-fitting (STAR Methods and Data S1, section 13).

Our model makes several assumptions: that all demographic events happened in discrete epochs; that the non-Erfurt subgroup experienced no initial bottleneck; that there was no gene flow from non-AJ populations or between the AJ subgroups; that population splitting and merging coincided with the start and end of the bottleneck, respectively; and that the bottleneck remained of a constant size throughout its duration. Given that these assumptions are very specific, our results should not be interpreted as a statement on the correct form of the demographic model: we cannot rule out alternative models, particularly more complex ones. Our results serve as a proof-by-example illustrating how a model of substructure, with different groups undergoing different bottleneck intensities, can reproduce the modern and ancient haplotype sharing data.

### Pathogenic variants

If EAJ have experienced the AJ founder event, we expect them to carry some of the pathogenic variants enriched in present-day AJ. We compiled a list of 62 pathogenic variants (Carmi et al., 2014a), after excluding variants with high frequency in Europeans and East-Asians (STAR Methods and Data S2, Table 6). As only six of these were included in our 1240k enriched SNPs, we used imputation to search for additional variants. We used a reference panel of 702 MAJ whole-genomes (Carmi et al., 2014a; Lencz et al., 2018) and two imputation methods. The first, which we call PHCP (Pseudo-Haploid ChromoPainter), takes pseudo-haploid SNP data as input. It is based on the diploid Li and Stephens hidden Markov model (Lawson et al., 2012; Li and Stephens, 2003), as we describe in the STAR Methods. The second is GLIMPSE, based on genome-wide sequence data (Rubinacci et al., 2021). We validated the high accuracy of PHCP using Mendelian consistency and masking of genotyped founder alleles, as well as confirmed high concordance between the two methods (Data S1, section 14 and Data S2, Table 6).

We discovered 11 high-confidence pathogenic variants in the EAJ genomes (STAR Methods and Table 1). Five additional variants were detected with low confidence (STAR Methods and Data S2, Table 6). The high confidence variants were carried by eight EAJ individuals, with each variant appearing once (Data S2, Table 6). Six of the discovered variants were previously dated using modern genomic data, with their estimated times of origin consistent with presence in the 14^th^ century (Data S1, section 14). Most of variants are in genes included in pre-conception screening panels (Table 1). A caveat is that imputation demonstrates the presence of *haplotypes* carrying the variants, not the variants themselves. However, the low false positive rate we observed with genotyped founder alleles (Data S1, section 14) argues against this scenario being common.

Finally, we assessed variants associated with selected non-medical or polygenic phenotypes. Variants for lactase persistence, eye and hair pigmentation, and (putatively) plague risk had similar allele frequency between EAJ and MAJ (Data S1, section 15). Osteological stature estimates for 13 adults were correlated with polygenic scores for height, although not significantly (Data S1, section 15).

## DISCUSSION

By analyzing genome-wide data from historical AJ individuals, we refine our understanding of early AJ origins. The ancestry of EAJ was closely related to that of MAJ, as evidenced by the PCA, ADMIXTURE, and qpWave analyses, suggesting a high degree of continuity of AJ ancestry over the past ≈700 years. However, EAJ individuals had more variable ancestry than MAJ and were stratified by the presence of a minor Eastern European-related ancestry component. Multiple lines of evidence suggest that the EAJ population had already experienced a “bottleneck” shared with MAJ: the high frequency of Ashkenazi founder mtDNA lineages, the presence of AJ-enriched pathogenic variants and other alleles, and long runs of homozygosity. In agreement with previous studies (Carmi et al., 2014a; Granot-Hershkovitz et al., 2018; Santiago et al., 2020), we date the onset of AJ expansion to about 20–25 generations ago (Data S1, section 16).

Our ancient DNA data allowed us to identify patterns in the history of AJ that would not have been detectable from modern genetic variation. Specifically, our results suggest that the AJ population was more structured during the Middle Ages than it is today. Within Erfurt, one group had an enrichment of Eastern European-related ancestry (Figures 2 and 3B), while the other had ancestry close to that of MAJ of Western European origin and modern Sephardi Jews (Figures 2 and S2). The two groups also had distinct levels of enamel δ^18^O (Figure 3C). Our results cannot exclude even finer divisions within Erfurt, and medieval AJ may have been additionally structured beyond Erfurt (Figure 4E). In contrast, present-day AJ are remarkably homogeneous (Guha et al., 2012; Gusev et al., 2012; Kopelman et al., 2020). This suggests that even though the overall sources of ancestry remained similar between medieval and modern AJ, endogamy and within-AJ mixture since medieval times have contributed to the homogenization of the AJ gene pool.

We can speculate on the identity of the two Erfurt subgroups based on non-genetic data suggesting that in the late Middle Ages, the AJ population was divided linguistically and culturally along a west/east axis (Beider, 2015; Katz, 1993) (Data S1, section 16). The Western AJ group, which may correspond to Erfurt-ME, likely represented descendants of the early AJ settlers in the Rhineland. One source explicitly mentions Erfurt as lying on the boundary between the two groups (Weinreich, 2008), and in the 14^th^ century, given names in Erfurt were typical of both Western and Eastern AJ (Beider, 2001). These points, together with evidence on migration into the second Erfurt community from the East (Data S1, section 2), may explain why individuals belonging to both genetic groups were present in Erfurt. The increased genetic diversity in Erfurt does not contradict our inference that EAJ experienced a more severe bottleneck than MAJ: while EAJ descended from fewer founders compared to MAJ, these founders were likely more variable in terms of their European genetic ancestry.

### Limitations of this study

We caution against interpreting the qpAdm models for the ancestral sources of EAJ as quantifying direct contributions of specific populations to the early AJ gene pool. This is because (1) the wide range of inferred ancestry proportions across models; (2) the historical fluctuations in ME ancestry in Italy (Antonio et al., 2019); and (3) the large space of models not explored here. Instead, the results reflect genetic links between the ancestral sources of AJ and modern populations, who may or may not have genetic continuity with their ancient antecedents. Further caveats are our inability to identify a satisfactory model for modern AJ and the technical difficulties when modeling an ancient population using modern sources with qpAdm (Harney et al., 2021) (although see our robustness tests; Table S3 and Data S1, section 7).

As with other ancient DNA studies, our historical inferences are based on a single site in time and space. This implies that our data may not be representative of the full genetic diversity of early AJ, as we have indeed inferred (Figure 4E). However, even for a single site, our sample size was relatively large (>30), and we were able to capture substructure not present in MAJ. Further, we were able to show genetic continuity with MAJ, as well as sharing of the same founder event. Another limitation is the reliance of our demographic models on a relatively small number of short runs of homozygosity (Figures 4D and S5), which are difficult to infer from pseudo-haploid data. In general, our inferred demographic model (Figure 4E) should not be interpreted as a full and precise demographic reconstruction; rather, it should be viewed as a simplified model (perhaps one among many) that captures the main features of the observed genetic data.

## STAR★METHODS

### RESOURCE AVAILABILITY

#### Lead contact

Further information and requests for resources or reagents should be directed to and will be fulfilled by the lead contact, Shai Carmi (shai.carmi@huji.ac.il).

#### Materials availability

This study did not generate new unique reagents.

#### Data and code availability

- The genotypes for the ancient Erfurt individuals have been deposited at https://reich.hms.harvard.edu/datasets. The sequencing reads have been deposited at the European Nucleotide Archive under accession number PRJEB53475. All other ancient genomes and the Human Origins array data for modern populations are publicly available at https://reich.hms.harvard.edu/allen-ancient-dna-resource-aadr-downloadable-genotypes-present-day-and-ancient-dna-data. The modern Ashkenazi Jewish genomes are available at https://ega-archive.org/datasets/EGAD00001000781. The isotope data have been deposited at IsoBank under ID number 686.
- Original code of PHCP imputation has been deposited at https://github.com/ShamamW/PHCPImpute (DOI: https://doi.org/10.5281/zenodo.7233296) and is publicly available. All other software packages used in this study were previously published.
- Any additional information required to reanalyze the data reported in this paper is available from the lead contact upon request.

### EXPERIMENTAL MODEL AND SUBJECT DETAILS

#### Archaeology

The medieval Jewish cemetery of Erfurt served the Jews of Erfurt and the surrounding area (Data S1, section 2). After the expulsion of the Jews from Erfurt in 1454, a barn and a granary were built on top of the cemetery. An archaeological rescue investigation was performed in 2013 during the conversion of the granary into a multi-story car garage (Data S1, section 3).

The excavated region, of about 16 × 12 m, is located between the inner and outer city walls. This section was probably used for burial only after the construction of the outer wall, as before that time it was likely used for fortification. Given archaeological evidence that the outer wall was built in the second half of the 14^th^ century (Data S1, section 3), the excavated region was probably used by the second community (the one that returned to Erfurt five years after the 1349 pogrom).

The excavation identified 47 burials, which is likely a small fraction of the total number of graves in the cemetery (Data S1, section 3). Our genetic study was performed in 2018, using only detached teeth, after receiving the approval of the Jewish community of Thuringia. We collected 38 teeth, one per individual, mostly molars (Data S2, Table 1). In 2021, all skeletons were reburied in the Jewish cemetery of the 19^th^-century community (Data S1, section 3).

### METHOD DETAILS

#### Age at death estimation

We estimated the age at death for non-adult individuals by assessing the developmental stage of the dentition (Ubelaker, 1978) and the length of the long bones (Stloukal and Hanáková, 1978). In adult individuals, we estimated the age at death by established methods based on the stage of degeneration of the pubic symphysis face, the auricular joint face, the sternal rib ends, and additionally the cranial suture closure, as summarized e.g., in (Buikstra and Ubelaker, 1994). Adult age was determined in an individual when the epiphyses were fused.

#### Radiocarbon dating

We performed accelerator mass spectrometry radiocarbon dating on purified collagen extracted from tooth dentin at the Pennsylvania State University Accelerator Mass Spectrometry (AMS) laboratory using previously described methods (Kennett et al., 2017; Lohse et al., 2014). We calibrated dates using OxCal 4.4 (Bronk Ramsey, 2009) and the IntCal20^48^ calibration curve (Reimer et al., 2020). We assessed sample quality using stable isotope analysis. We found that carbon-to-nitrogen ratios for all collagen samples fell between 3.19 and 3.24, within the range of 2.9–3.6 expected for good collagen preservation (van Klinken, 1999).

The estimated dates are reported in Data S2, Table 1 (95.4% probability intervals) and the full measurements are reported in Data S2, Table 3. Our first attempt at dating the father and daughter from Family B (I14904 and I13869, respectively) yielded results inconsistent with their relationship (95.4% CI 1266–1298 calCE for the daughter and 1288–1398 calCE for the father). We radiocarbon dated these samples again, obtaining the same result for the father (1297–1395 calCE). For the daughter, the second run suggested a range of dates consistent with the relationship (1278–1380 calCE), due to the appearance of a small post-1350 peak (Figure S1C).

We note that we dated teeth, which form in childhood and early adulthood. Thus, all dates should not be interpreted as representing date of death, but instead a date during an earlier time of life.

#### Isotope analysis

To test the hypothesis that some Erfurt individuals were migrants, we selected enamel samples for δ^13^C and δ^18^O isotope analysis at the University of New Mexico Center for Stable Isotopes. Among samples with sufficient material, we considered 20 samples with coverage >200k SNPs, which were all confidently assigned to an Erfurt subgroup (EU/ME).

Enamel surfaces were cleaned with a rotary tool and a 200 μm endmill used to remove about 30 mg of enamel, avoiding any dentin. Samples were crushed in an agate mortar with UltraPure water (resistivity = 18.2 MΩ cm) to keep chips from shattering. About 15 mg of sample was transferred to a 2 mL centrifuge tube with about 0.1 M Acetic Acid and agitated for about 30 s using a VortexGenie to sufficiently mix the sample and solution. Samples were reacted for about 4 h, then rinsed to neutrality with UltraPure water and micro-centrifuged for 1 min at 3000 rpm between each rinse. After the final rinse samples were freeze dried overnight (about 12 h). Next, 7–8 mg of sample was weighed into 12 mL glass exetainers for analysis. An additional 1 mg aliquot of sample was separated and placed on the ThermoFisher Nicollet Summit FTIR diamond ATR for QC/QA analysis. Samples were analyzed on a Thermo Scientific Delta V IRMS with a GasBench carbonate device for δ^13^C_enamel_ and δ^18^O_enamel_.

The full results of the isotope analysis are reported in Data S2, Table 5, and the final values of δ^13^C_enamel_ and δ^18^ O_enamel_ appear in Data S2, Table 1.

#### DNA extraction and sequencing

Of the 38 teeth, four did not have root material and were not further processed. For the remaining 34, we performed the following steps. In dedicated clean rooms at Harvard Medical School, we drilled into the roots of the teeth to obtain powder from cementum and dentin. We extracted DNA using a protocol meant to retain short molecules (Dabney et al., 2013); converted the DNA into individual barcoded and/or indexed double-stranded and single-stranded libraries treated to remove characteristic ancient DNA damage (Briggs and Heyn, 2012; Gansauge et al., 2017, 2020; Rohland et al., 2015); enriched for approximately 1.24 million SNPs (Fu et al., 2015) and mitochondrial DNA (Maricic et al., 2010); and sequenced the enriched and non-enriched libraries on Illumina instruments (Data S2, Table 2).

#### Bioinformatics and quality control

We merged read pairs, requiring at least 15 overlapping base pairs, and allowing no more than one mismatch if base quality was at least 20, and as many as three mismatches if base quality was less than 20, and chose the nucleotide of higher quality when we observed mismatches. We mapped the sequences to the human genome reference sequence hg19 (GRCh37, https://www.ncbi.nlm.nih.gov/assembly/GCF_000001405.13/) and the inferred mitochondrial ancestral sequence RSRS (Behar et al., 2012) using the *samse* command of *BWA* version 0.7.15 using parameters -n 0.01, -o 2, and -l 16500 (Li and Durbin, 2009). We removed duplicate molecules that mapped to the same start and stop positions and (for double-stranded libraries) that had the same molecular barcodes.

We analyzed the data to assess ancient DNA authenticity based on the following metrics (Data S2, Table 2). First, the ratio of Y to X + Y chromosome sequences: uncontaminated females should have a very low (<0.030) and males should have a high (>0.35) ratio in the type of data we produced. Second, the estimated rate of variation in the mitochondrial genome and the X chromosome in males at known polymorphisms: uncontaminated individuals should be consistent with very little variation. We estimated the degree of contamination with *contamMix* version 1.0–12 (Fu et al., 2013) for the mitochondrial DNA and *ANGSD* for the X chromosome (Korneliussen et al., 2014). Third, we required an appreciable rate of cytosine to thymine damage in the final nucleotide, as expected for genuine ancient DNA. We initially obtained DNA data for 34 individuals. However, one individual was represented at only 52 SNPs, and was omitted from all analyses.

We determined mitochondrial haplogroups using *HaploGrep2* (Weissensteiner et al., 2016). The procedure for Y-chromosome haplogroup determination is described in Supplementary Text S5 of (Lazaridis et al., 2022), using the YFull YTree v. 8.09 phylogeny (https://github.com/YFullTeam/YTree/blob/master/ytree/tree_8.09.0.json), obtaining information about SNPs from ISOGG YBrowse (https://ybrowse.org/gbrowse2/gff/snps_hg38.csv; accessed Oct 18, 2020), lifting coordinates from hg38 to hg19 using liftOver and intersecting with the SNPs present in the v. 8.09 tree. The haplogroup calls were converted into letter-number haplogroup designations (e.g., J2a1) using the phylogeny of the International Society of Genetic Genealogy v. 15.73. We could not infer the haplogroup of four males due to insufficient coverage over the informative Y-SNP targets. The method we used to determine the terminal mitochondrial and Y chromosome lineages is described in Data S1, section 4.

#### Pathogen DNA scan

We screened the Erfurt individuals for the presence of pathogens using *MALT* (Herbig et al., 2016; Vagene et al., 2018). We used a custom *RefSeq* genomic dataset containing bacteria, viruses, eukaryotes, and the human reference sequence GRCh38 to construct the *MALT* database using default parameters (Giffin et al., 2020), with an index step size of 6. For each Erfurt individual, we screened all merged, de-duplicated sequences, applying a minimum complexity filter with a threshold of 0.3. We ran *MALT* (version 0.3.8), using the parameters –mode BlastN, –alignmentType SemiGlobal, –minPercentIdentity 0.85, –topPercent 1, –minSupport 1, -maxAlignmentsPerQuery 100. We then screened the results using the *HOPS* workflow (Hubler et al., 2019) to determine whether there was evidence of authentic DNA from a set of 348 pathogens of interest (Data S2, Table 4). We assessed authenticity using a standard three step screening pipeline considering (1) the edit distance distribution of all sequences that align to the pathogen of interest; (2) the presence of C-to-T (or G-to-A) sequence damage (which is characteristic of authentic ancient DNA); and (3) the edit distance distribution of the subset of aligned sequences that contain C-to-T damage (Hubler et al., 2019).

### QUANTIFICATION AND STATISTICAL ANALYSES

#### Detecting genetic relatives

We used *READ* (Monroy Kuhn et al., 2018) to identify 1^st^- and 2^nd^-degree relatives. We identified five individuals with 1^st^-degree relationships (Figure S1A). Three individuals — I14850 (female), I14853 (male), and I14898 (female) – were all identified as 1^st^-degree relatives of one another, and we denoted them as Family A (Figure 1B). To determine their relationships, we first used information on age at death and mitochondrial DNA haplogroup. Individuals I14853 and I14898’s estimated ages were 14–17 and 10–13 years old, respectively (Data S2, Table 1), and hence they must have been siblings. I14850 was estimated to die in her 40s, and all three individuals shared the same mtDNA haplogroup. These constraints are consistent with I14850 being either the mother or a sibling who has survived to adulthood. To distinguish between these two possibilities, we calculated the mismatch rate along the genome between I14850 and I14853 (who had the higher coverage among the two sibs). We compared the mismatch rate to that of (*i*) unrelated individuals; and (*ii*) two sets of sequences sampled of the same individual. In (*ii*), we only used SNPs covered by at least two sequences, and we generated each duplicate by sampling a single sequence (without replacement) for each SNP. We calculated the mismatch rate in blocks of 20 Mb and normalized it to the number of SNPs. Figure S1B shows that the mismatch rate between I14850 and I14853 is consistently intermediate between that of unrelated and duplicate individuals, as expected for a parent-child relationship. We thus conclude that I14850 is the mother of I14853 and I14898. Two other individuals — I13869 (female) and I14904 (male) – were 1^st^-degree relatives and were designated as Family B. As they carried different mtDNA haplogroups, the only possible relationship is that of a father and a daughter.

Individual I14855 was found by *READ* to be a 2^nd^-degree relative of I14854 and I14848. The latter two individuals had a low mismatch rate but were not classified as 2^nd^-degree relatives, possibly due to their very low coverage (15k and 13k SNPs, respectively). I14854 and I14855 carried the same mtDNA haplogroup, but not I14848. The uncertainty ranges for the two identified relationships included a 1^st^-degree relationship and no relationship (Figure S1A).

#### Investigating genetic ancestry using PCA

We used *smartPCA* (Patterson et al., 2006), with the option “lsqproject”, which enables projection of individuals with high missingness when the PCs were learned from modern individuals. The following populations from the Human Origins dataset were used: Armenian, Iranian, Turkish, Albanian, Bergamo, Bulgarian, Cypriot, Greek, Italian_South, Maltese, Sicilian, Italian_North, English, French, Icelandic, Norwegian, Orcadian, Scottish, BedouinA, BedouinB, Jordanian, Palestinian, Saudi, Syrian, Abkhasian, Adygei, Balkar, Chechen, Georgian, Kumyk, Lezgin, Ossetian, Jew_Ashkenazi, Jew_Georgian, Jew_Iranian, Jew_Iraqi, Jew_Moroccan, Jew_Tunisian, Jew_Libyan, Jew_Turkish, Jew_Yemenite, Basque, Spanish, Spanish_North, Druze, Lebanese, Belarusian, Croatian, Czech, Estonian, Hungarian, Lithuanian, Ukrainian, Canary_Islander, Sardinian, Finnish, Mordovian, Russian, and Polish.

We projected the Erfurt individuals on the PC space learned by the West-Eurasian populations. Down-sampling experiments showed that 50k or more SNPs provide reproducible assignment of individuals to Erfurt subgroups (Data S1, section 5). We consequently excluded eight EAJ individuals with <50k SNPs from all PC analyses.

We also ran PCA with a larger sample size of MAJ, as follows. We merged the Human Origins dataset with whole-genomes of *n* = 544 modern AJ sampled in the USA and Israel (Lencz et al., 2018). These genomes were generated in Phase 2 of The Ashkenazi Genome Consortium (TAGC), and relatives were removed (Fridman et al., 2020). The merged dataset had about 470k SNPs. We used the same Human Origins populations as above to learn the PC space, except that here we removed the Human Origins MAJ population. We then projected both EAJ and the TAGC MAJ on the PC space. Due to the large MAJ sample size, we plotted the positions of these individuals as a 2-dimensional kernel density plot (Figure S2A) using the function *stat_density_2days()* from the package *ggplot2* in *R*. The density was scaled to 1 using the argument *contour_var = “ndensity”*. To evaluate the effect of coverage on placement in PC space, we down-sampled a randomly selected subset of *n* = 525 MAJ individuals to match the set of SNPs covered by the EAJ genomes. Each of the 25 (non-low-coverage) EAJ genomes was used to match 525/25 = 21 MAJ individuals. We then selected a single allele at random from each down-sampled MAJ individual and projected the MAJ and EAJ individuals as above (Figure S2B). See Data S1, section 5 for details on the PCA that included MAJ of Western and Eastern European origin.

#### Admixture modeling with *ADMIXTOOLS*

For the f_4_ and *qpWave* analyses, we used the Human Origins dataset merged with whole-genomes of *n* = 544 modern AJ (Lencz et al., 2018). We used *ADMIXTOOLS* (Patterson et al., 2012) version 5.1 for running the analyses. We computed f_4_-statistics with default parameters. In the *qpWave* and *qpAdm* analyses, we only used transversion SNPs (about 110k) to avoid bias due to ancient DNA damage. In all population-level analyses, we (1) omitted one of each pair of first-degree relatives, keeping the individual with the higher coverage; and (2) included the low-coverage individuals (<50k SNPs), except in analyses that required the Erfurt-EU and Erfurt-ME group affiliation.

#### Detecting admixture with *qpWave*

We ran *qpWave* with the option “allsnps:YES”, which means that in each f_4_ test, the program uses all SNPs that were non-missing in all four populations, but a different set of SNPs can be analyzed for each underlying f_4_-statistic. We ran *qpWave* tests separately against reference (“right”) European and reference Middle Eastern populations. In our *qpWave* analyses with European reference populations, we chose the right populations as modern populations that represent main European ancestries: Russian, Norwegian, French, Spanish, Bulgarian, and Italian_North, with Primate_Chimp as an outgroup (first right population). Middle Eastern populations are closely related, and we therefore chose the right populations as follows. We ran f_4_ tests of the form f_4_(EAJ, MAJ; ME1, ME2) where ME1 and ME2 represent all possible pairs of populations from: BedouinA, BedouinB, Palestinian, Lebanese, Syrian, Jordanian, Egyptian, Saudi, and Druze. We used populations that were involved in tests with p value<0.05: Druze, Lebanese, Jordanian, and BedouinA. We again used Primate_Chimp as an outgroup. In tests with South-Italians, we used individuals of Sicilian and Italy_South together as one group.

#### Inferring admixture models with *qpAdm*

Here too, we used the option “allsnps:YES”. The reference populations (right populations) for the *qpAdm* analyses were: Mbuti, Ami, Basque, Biaka, Bougainville, Chukchi, Eskimo_Naukan, Han, Iranian, Ju_hoan_North, Karitiana, Papuan, Sardinian, She, Ulchi, and Yoruba. Mbuti was used as the outgroup (provided to *ADMIXTOOLS* as the first in the list of reference populations) in all analyses. In robustness tests, we replaced Mbuti with Ami as the outgroup. As in the *qpWave* analyses, in models with South-Italians we used individuals of Sicilian and Italy_South together as one group. In models with ancient Germans, we used individuals from (Veeramah et al., 2018), not including individuals with elongated skulls or with Southern European ancestry. The ancient Levant (Canaanite) individuals (Agranat-Tamir et al., 2020) were from Bronze-Age Megiddo (Megiddo_MLBA) and the ancient Rome individuals (Antonio et al., 2019) were from Late Antiquity (Italy_LA.SG) and Imperial Rome (Italy_Imperial.SG).

For the analyses at the individual level, we used all SNPs, as the coverage of many individuals was already low. To guarantee that using all SNPs did not bias the results, we repeated the analyses at the population level with all SNPs instead of just transversions and verified that the results remained qualitatively unchanged (Data S1, section 7). We included first-degree relatives in the individual-level analyses, but omitted the low-coverage individuals (<50k SNPs). For individuals for which the Eastern-EU ancestry proportion was inferred to be negative (Figure 3), we re-ran *qpAdm* with only Southern EU and Middle Eastern sources.

#### Inferring admixture time with *DATES*

We attempted to estimate the time of Eastern European gene flow into Erfurt-EU using *DATES* (Chintalapati et al., 2022) (Data S1, section 8). However, our simulations showed that *DATES* estimates in our setting were unreliable (Data S1, section 8).

#### Maximal post-medieval gene flow into AJ

The *qpWave* test comparing EAJ and MAJ gave p = 0.15 (Table S1), consistent with these groups being a clade with respect to reference European populations. To estimate the maximal degree of post-medieval gene flow into AJ that would still be consistent with this result, we used simulations. Specifically, we simulated AJ groups that have experienced increasing magnitudes of admixture with Eastern European sources. We then tested, using *qpWave* (with respect to the same European populations as described above), which simulated group is still inferred as a clade with modern AJ. The Eastern European sources included 30 individuals from Belarusians, Lithuanians, Ukrainians, and Russians. These individuals were removed from the subsequent *qpWave* analyses. The AJ source was 30 MAJ individuals that were sequenced in Phase 1 of The Ashkenazi Genome Consortium (Carmi et al., 2014a) and were not included in the MAJ dataset that was used for the original *qpWave* analyses. For each admixture scenario, we simulated 30 individuals using the procedure described in Data S1, section 6. We used admixture times of *G* = 5; 10; 15; 20 generations.

The *qpWave* p values reported in Table S2 show that the maximal proportion of the AJ gene pool that could have been replaced by East-EU admixture and still remain consistent with being a clade with MAJ is about 2%–4%. In our simulations, all gene flow was assumed to occur over a single generation. With continuous gene flow, a replacement of a proportion *m* of the gene pool per generation over 20 generations would lead to a total replacement of 1 – (1 – *m*)^20^ of the total ancestry. Equating to 4% and solving for *m* gives *m* = 1 – (1 − 0:04)^1/20^ = 0.2%.

#### Correlation between EAJ grave location and group affiliation

We used the Mantel test to investigate the correlation between group affiliation and the distances between the graves in the cemetery. We calculated the distances based on the approximate coordinates of the skeletons’ heads in the cemetery map (Figure 1A). For group affiliation, we set the distance between Erfurt-ME and Erfurt-EU to 1 and the distance within each group to zero. We used the function *mantel.rtest* from the *ade4* package in *R*. We also ran the Mantel test by replacing the group affiliation distance with the distances in the PC1-PC2 space, based on the PCA of Figure 2 of the main text and using Euclidean distances.

#### Aligning the mtDNA sequences of modern and ancient K1a1b1a carriers

To obtain the sequences of modern K1a1b1a carriers, we used *n* = 544 genomes of Phase 2 of The Ashkenazi Genome Consortium (after removing related individuals) (Lencz et al., 2018). We first discarded sites with GQ < 40 and indels. Some sites had heterozygous genotypes (possibly due to heteroplasmy). We encoded these sites as having the alternate allele if the allele was observed at more than 50% of the reads and otherwise encoded them as having the reference allele. We used *bcftools consensus* (Li, 2011) with parameter -H A using the rCRS reference sequence to generate an alignment of the sequences of all individuals. We then used *HaploGrep*2 (Weissensteiner et al., 2016) to call mitochondrial haplogroups and focused on the 107 carriers of K1a1b1a. For the ancient individuals, haplogroups were called as described above and we only considered the 11 K1a1b1a carriers. Their median coverage was 304× (range: 47–447×; Data S2, Table 2). We discarded indels and otherwise performed no additional filtering. We again used *bcftools consensus* to generate an alignment of the sequences, but this time with the RSRS reference sequence (Behar et al., 2012).

#### Estimating the time to the most recent common ancestor (TMRCA) of the K1a1ba1 carriers

To estimate the TMRCA of the K1a1ba1 lineage, we used a Bayesian coalescent analysis, as implemented in *BEAST* 2 (v2.6.6) (Bouckaert et al., 2019). The input was the mtDNA sequence alignment of the 11 EAJ carriers and the 107 MAJ carriers. We set the dates of the EAJ individuals to 650 years ago. For modeling mutations, we used the HKY model with Gamma distributed rates (four categories), and the strict clock model. For the population size prior, we used the coalescent Bayesian skyline with four segments. We also ran *BEAST* with a coalescent exponential population prior, but convergence was poor and we did not further consider this prior. For the skyline model, we ran 10 chains for 100 million steps each. For each chain, we used 10% of the steps as burn-in and recorded the parameters every 100,000 steps, resulting in a total of 9000 samples. All other parameters were set to their default values. We combined the samples from all chains using *LogCombiner* and visualized the results using *Tracer* and *FigTree*.

#### Detecting runs of homozygosity in the ancient genomes

To identify runs of homozygosity (ROH) in the ancient genomes, we used the Python package *hapROH* version 0.1a8 (Ringbauer et al., 2021), using 5,008 global haplotypes from the 1000 Genomes Project as the reference panel and the pseudo-haploid genotypes of the ancient genomes as input. As recommended for datasets with genotype data for 1240k SNPs (Ringbauer et al., 2021), we applied our method to 16 Erfurt individuals covered in at least 400k SNPs and called ROHs longer than 4 cM. These individuals include two parent-child pairs. We used the default parameters and post-processing of *hapROH*, which are optimized for this data type (described in detail in (Ringbauer et al., 2021)). We report the sum of the lengths of all ROHs longer than 4 cM in each individual in Data S2, Table 1.

We manually inspected the ROH results by examining the positions of putatively heterozygous sites (Figure S4). Given that the called genotypes are pseudo-haploid (representing the allele of a randomly selected read), information on heterozygosity requires the original sequencing reads. For each SNP, we obtained the read counts for each allele from the processed BAM files using *samtools mpileup* (Li, 2011). Due to the low coverage, high quality diploid genotype calls could not be made. We identified putatively heterozygous sites as SNPs with at least one read supporting each of the reference and alternate alleles. While not all heterozygous sites could be detected using this approach (due to the low coverage), their depletion at inferred ROH segments is evident (Figure S4). There was no correlation between ROH counts and coverage (Data S1, section 10).

To contextualize the ROH results, we repeated the ROH analysis in other ancient populations. We used the Allen Ancient DNA resource (V50.0, Oct 10 2021; https://reich.hms.harvard.edu) and extracted previously published genomes from individuals who lived in the past ≈2000 years. We downloaded the data in *eigenstrat* format. As above, we used *hapROH* with the recommended settings (Ringbauer et al., 2021) and applied it to pseudo-haploid genotypes. The populations we studied included Hungary Langobard (Amorim et al., 2018); Germany Early Medieval (Veeramah et al., 2018); Italy Imperial, Late Antiquity, and Medieval (Antonio et al., 2019); and Denmark Viking (Margaryan et al., 2020). The sum of the lengths of ROH segments in individuals belonging to these populations is shown in Figure S4.

#### Ashkenazi founder alleles in Erfurt Jews

We defined founder alleles as those having minor allele frequency >0.5% in MAJ and <0.01% in non-Finnish Europeans (using *gnomAD* [Karczewski et al., 2020]). To exclude variants that may have a Middle Eastern source (which is not covered by *gnomAD*), we used 221 Middle Eastern individuals from the Human Origins dataset (Data S1, section 6, Table 1 therein), and excluded alleles that appeared more than once among these individuals. Finally, we removed SNPs that were genotyped in fewer than three EAJ individuals (after removing first-degree relatives), leaving a total of 216 SNPs.

Among the EAJ individuals, we excluded all children from families A and B, as well as one individual who was not genotyped in any of the founder SNPs, and was thus uninformative. Within the remaining 29 EAJ individuals, we detected 15 founder alleles in 11 individuals (Data S2, Table 1). All variants except one appeared in just a single individual. The remaining variant appeared in three individuals, and thus the total number of copies of these alleles was 17.

#### Binomial simulations of founder allele counts

To determine whether the number of founder alleles present in EAJ is as expected if EAJ had already experienced the AJ bottleneck, we used binomial simulations. In each run and for each founder allele, we drew an EAJ allele count as a binomial variable with *n* equals to the number of EAJ individuals that were genotyped in that SNP, and *p* equals to the allele frequency in MAJ (based on *gnomAD*). Note that we used the number of EAJ individuals (and not twice the number) as our genotypes are pseudo-haploid. In each run, we recorded the number of alleles (out of 216) that appeared in at least one individual. The distribution of the number of observed alleles across 10,000 runs is shown in Figure S5C. The results remained similar when modeling reference allele bias (Data S1, section 11).

#### Detecting IBD and ROH segments in modern genomes

We used sequencing data of *n* = 637 MAJ from the two phases of The Ashkenazi Genome Consortium (Carmi et al., 2014a; Lencz et al., 2018), after relatives and duplicates were removed (Fridman et al., 2020). To detect IBD sharing in MAJ, we used *IBDseq* (Browning and Browning, 2013) with default parameters. We detected runs of homozygosity in *n* = 574 genomes (Phase 2 data) using the software *bcftools/ROH* (Narasimhan et al., 2016). To meaningfully compare ROH in ancient and modern individuals, we first down-sampled the modern data to the 1240k SNPs used in the ancient DNA analysis. We used the same sex-averaged genetic map as in the analysis of the ancient data (provided with the *hapROH* reference panel), and set the hidden Markov model transition probabilities to parameters optimized for 1240k data (see (Ringbauer et al., 2021); toA = 6.7e-8, toHW = 5e-9). To obtain allele frequencies, we used the diploid data of the 574 modern individuals (using the VCF field MLEAF). As in the ancient data, we merged gaps of length up to 0.5 cM between two ROHs (both at least 2 cM long, one at least 4 cM). We manually inspected the ROH results by determining whether inferred ROH segments are depleted of heterozygous sites (Figure S4), considering only bi-allelic SNPs in the 1240k set. We finally removed nine individuals with total ROH length >50 cM (in segments longer than 4 cM), as these individuals likely have closely related parents.

#### Demographic inference using IBD and ROH segments

Complete details are provided in Data S1, section 12. Briefly, we searched for a model that would fit the total count of IBD segments between all pairs of individuals in 11 length bins between 4 and 15 cM (Figure 4B). The model of Figure 4A is characterized by five parameters. For each proposed set of values for these parameters, we computed the expected number of IBD segments based on theory developed in (Carmi et al., 2014b; Palamara et al., 2012; Ringbauer et al., 2017). For each length bin, and conditioning on the TMRCA, the theory provides the expected number of segments shared between two haplotypes in a chromosome of a given length. We then summed over all possible values of the TMRCA, with the probability of each TMRCA computed using coalescent theory given the currently assumed demographic model. We then summed over all chromosomes and multiplied by $\left(\begin{array}{l} \begin{array}{l} 2n\\ 2 \end{array} \end{array}\right)-n$ to sum over all (non-ROH) pairs of haplotypes coming from *n* diploid individuals. The likelihood of the count in each length bin was computed by assuming a Poisson distribution for the number of segments with mean equal to the theoretical expectation. Finally, we constructed a composite likelihood by multiplying the likelihood across length bins. We searched for the parameters that maximize the log of the composite likelihood using the *R* package *DEoptim* (Mullen et al., 2011). To compute confidence intervals, we used parametric bootstrap. To generate each bootstrap replicate, we drew a new count for the number of segments, for each length bin, as a Poisson variable with mean equals to the real count.

When using ROH to infer the parameters of the demographic models, we used the same approach, based on counts of ROH segments across length bins, except for the following differences. First, the number of haplotype pairs was *n*. Second, we assumed no coalescence in the past two generations (reflecting no sib mating). Third, for modeling ROH in EAJ, we considered segments of length up to 40 cM. We assumed that the EAJ genomes were sampled 26 generations ago, and computed the coalescence probabilities accordingly. To model consanguinity, we assumed that a fraction of the individuals are children of couples who are first cousins. To estimate the parameters of a model with a narrower bottleneck than expected based on the modern data, we estimated *N*_*a*_ and *N*_*b*_ using ROH in EAJ, after fixing all other parameters to their values from Figure 4A. To infer a longer bottleneck, we estimated *T*_*b*_ using ROH in EAJ, after fixing all other parameters. In both cases, we then used the modern IBD data to re-estimate *d* and *N*_*c*_.

To estimate demographic parameters using both modern IBD and ancient ROH, we computed their expected counts based on the same demographic model, accounting for the sampling of the ROH segments 26 generations ago. We then computed the log-(composite) likelihood per haplotype pair separately for IBD and ROH, and computed the joint log-likelihood as their sum. This guarantees that each data type contributes roughly equally to the likelihood. Finally, to estimate the parameters of the two-population model, we used the same approach as above, except that we computed the coalescence probabilities taking into account that two lineages can coalesce during the period of population split only if both descended from the same sub-population.

We used parametric bootstrap for model selection, using simulations to generate the empirical null distribution of the increase in log-likelihood when fitting a two-population model to data simulated under a a single-population model (Data S1, section 13). Specifically, we simulated segment counts under the single-population model as Poisson variables with means equal to the theoretical expectations. We then compared the increase in the log-likelihood when fitting the data to a two-population (vs a single-population) model to the increase observed in the real data (Data S1, section 13).

#### Defining pathogenic founder variants

We started with Supplementary Table 4 from an earlier paper analyzing AJ sequencing data (Carmi et al., 2014a). We excluded 11 variants that were not present in modern AJ (based on *gnomAD*) or had higher frequency in other populations in *gnomAD*, leaving 62 variants. Among them, 47 were present in our reference panel and could be imputed. None of the pathogenic variants appeared in the list of AJ founder alleles defined above, as most of them were not enriched. Of the enriched variants, four had frequency >0.01% in Europeans, one was not genotyped in the modern Middle Eastern individuals, and one did not appear in *gnomAD*.

#### Imputation using pseudo-haploid ChromoPainter (PHCP)

We developed a method for imputation of pseudo-haploid genotypes based on the previously reported *PHCP* model (Agranat-Tamir et al., 2020; Wasik et al., 2021). Briefly, *PHCP* (Pseudo-Haploid *ChromoPainter*) is an extension of the *ChromoPainter* model (Lawson et al., 2012), which itself is based on the Li-Stephens (Li and Stephens, 2003) hidden Markov model (HMM). *PHCP* models a target ancient sequence as a mosaic of modern haplotypes (“donors”). For each SNP, the hidden state of the HMM is a pair of modern haplotypes from which the target is “copied.” The observed (haploid) ancient allele is assumed to derive from each of these two haplotypes with equal probability. Transitions between donor haplotypes along the target sequence are assumed to be due to ancestral recombinations, and emissions model the combined effect of mutations, gene conversion events, and genotyping and other errors that lead to imperfect copying of the donor haplotypes. The full transition and emission probabilities were previously described in (Agranat-Tamir et al., 2020), where we used the population labels of the inferred donors of each target to learn about the target’s ancestry composition.

To impute an ancient target genome, we used the forward-backward algorithm. We computed, for each SNP of the target, the marginal posterior probability of each possible pair of donor haplotypes. For SNPs in the reference panel that were not enriched or were not covered in the target, we assigned to the target the marginal probabilities of the nearest covered SNP (in cM). For each SNP, we divided all haplotype pairs into three classes, based on the diploid genotype they imply for the target (AA/AB/BB). For each of the three possible genotypes, we defined their marginal probability as the sum of the marginal probabilities of all pairs of donors in that genotype’s class. In downstream analyses, we used for each SNP the most likely genotype.

To impute the EAJ genomes, we used *n* = 702 MAJ genomes (both phases of the Ashkenazi Genome Consortium (Carmi et al., 2014a; Lencz et al., 2018); without removing relatives or other individuals). We considered only autosomal chromosomes. We set the parameters for *PHCP* to *N*_*e*_ = 64:57; *θ* = 0:0014 (Agranat-Tamir et al., 2020). To reduce running time, we used, for each chromosome, a set of 200 donor haplotypes that were most informative for that chromosome. The donor haplotypes were ranked based on the number of SNPs where they share an alternate allele with the target (Wasik et al., 2021) (and scaled by the number of SNPs where they have an alternate allele, as in (Wasik et al., 2021), although we used all available sites and not just the rare variants). The *PHCP* imputation software is available at https://github.com/ShamamW/PHCPImpute.

#### Imputation using *GLIMPSE*

We used *GLIMPSE* (v1.0.0) (Rubinacci et al., 2021) with *n* = 702 MAJ individuals as the reference panel (as for *PHCP*). Diploid genotype calls were generated using *bcftools mpileup* (v1.10.2) (Li, 2011). We imputed all autosomal bi-allelic SNPs and indels in MAJ. However, genotype likelihoods were used only for bi-allelic SNPs (generated by *mpileup*) as input to build the phasing and imputation model. Indels were imputed without genotype likelihood information, due to their more severe reference bias.

#### Analyzing the pathogenic founder variants detected in Erfurt Jews

After imputing the EAJ genomes with *PCHP* and *GLIMPSE*, 16 pathogenic variants were either directly genotyped or successfully imputed (Data S2, Table 6). We defined a set of high-confidence pathogenic variants present in EAJ based on the following criteria: (i) the *PHCP* probability for having at least one alternate allele was >97% and (ii) the alternate allele was detected by *GLIMPSE* with probability >50%. One variant (*CLRN1*, NP_001182723.1:p.N48K) was detected by *PHCP* with probability >97% in an individual that was not imputed with *GLIMPSE* due to its low coverage (Data S2, Table 6); we considered this variant as high-confidence. We also defined a set of low-confidence pathogenic founder variants as follows: (i) *PHCP* marginal probability >97% and *GLIMPSE* probability <50%; or (ii) *PHCP* probability in the range 50%–97%; or (iii) *PHCP* marginal probability <50% and *GLIMPSE* probability >50% (Data S2, Table 6).

For each high-confidence variant detected in EAJ, we determined whether the corresponding gene is present in pre-conception carrier screening (PCS) panels. We considered four PCS sequencing-based panels aimed at the AJ population: Genpath (https://www.genpathdiagnostics.com/hcp/womens-health/carrier-screening/ashkenazi-jewish-cancer-screening/), SEMA4 (https://sema4.com/elements/expandedcarrierscreen/), fulgent (https://www.fulgentgenetics.com/beacon-ashkenazi-jewish-female-carrier-screening) and Baylor genetics (https://www.baylorgenetics.com/geneaware/). All data were downloaded in November 2020. For each variant, we indicate in Table 1 the number of panels in which the gene is included.

## Supplementary Material

MMC2

MMC1

1

## Figures and Tables

**Table 1.** High-confidence AJ-enriched pathogenic variants detected in Erfurt

Disease	Gene	Variant	Carrier ID and PHCP probability of carrying at least one alternate allele	Frequency in modern AJ (gnomAD)	Frequency in non-Finnish Europeans (gnomAD)	Gene included in Ashkenazi PCS panels
Retinitis pigmentosa 59	*DHDDS*	c.124A>G	I14903, 0.985	0.52%	0.004%	4/4
Gaucher disease, type 1	*GBA*	c.84dupG	I13861, 1.000	0.08%	0.002%	4/4
Usher syndrome, type 3	*CLRN1*	p.N48K	I14897, 0.980	0.58%	0.008%	4/4
Factor XI deficiency	*F11*	p.E135X	I13870, genotyped	1.71%	0.03%	3/4
Factor XI deficiency	*F11*	p.F301L	I13865, genotyped	2.38%	0.03%	3/4
Cystic fibrosis	*CFTR*	p.G542X	I14736, 1.000	0.15%	0.04%	4/4
Parkinson’s disease	*LRRK2*	p.G2019S	I14739, genotyped	0.84%	0.03%	0/4
Acyl CoA dehydrogenase deficiency	*ACADS*	c.319C>T	I14737, 0.999	1.88%	0.02%	0/4
Familial Mediterranean fever	*MEFV*	p.V726A	I14739, 0.993	3.93%	0.09%	2/4
Glycogen storage disease, type 1A	*G6PC*	p.R83C	I13870, 0.970	0.66%	0.05%	4/4
Breast/ovarian cancer predisposition	*BRCA1*	c.68_69delAG	I13861, 1.000	0.41%	0.009%	0/4

For each of the 11 variants, we indicate the disease, the gene, and the variant in HGVS (Human Genome Variation Society) nomenclature. The c.68_69delAG BRCA1 variant is also known as 185delAG. Variants that were detected in sequences covering enriched SNPs are designated as “genotyped”. For imputed variants, we provide the marginal posterior probability in PHCP for having at least one alternate allele. We further provide the carrier IDs, the allele frequency in MAJ and non-Finnish Europeans (gnomAD), and the number (out of four) of Ashkenazi-specific pre-conception screening (PCS) panels where the gene is included (STAR Methods).

**Table:** KEY RESOURCES TABLE

REAGENT or RESOURCE	SOURCE	IDENTIFIER
Chemicals, peptides, and recombinant proteins		
2× HI-RPM hybridization buffer	Agilent Technologies	5190-0403
Herculase II Fusion DNA Polymerase	Agilent Technologies	600679
Pfu Turbo Cx Hotstart DNA Polymerase	Agilent Technologies	600412
50% PEG 8000	Anatrace	OPTIMIZE-82 100 ML
0.5 M EDTA pH 8.0	BioExpress	E177
Sera-Mag^™^ SpeedBead Carboxylate-Modified [E3] Magnetic Particles	Cytiva Life Sciences	65152105050250
silica magnetic beads	G-Biosciences	786-916
10 × T4 RNA Ligase Buffer	New England Biolabs	B0216L
Bst DNA Polymerase2.0, large frag.	New England Biolabs	M0537
UGI	New England Biolabs	M0281
USER enzyme	New England Biolabs	M5505
Buffer PB	QIAGEN	19066
Buffer PE concentrate	QIAGEN	19065
1 M Tris-HCl pH 8.0	Sigma Aldrich	AM9856
1 M NaOH	Sigma Aldrich	71463
20% SDS	Sigma Aldrich	5030
3 M Sodium Acetate (pH 5.2)	Sigma Aldrich	S7899
5 M NaCl	Sigma Aldrich	S5150
Ethanol	Sigma Aldrich	E7023
Guanidine hydrochloride	Sigma Aldrich	G3272
Isopropanol	Sigma Aldrich	650447
PEG-8000	Sigma Aldrich	89510
Proteinase K	Sigma Aldrich	P6556
Tween-20	Sigma Aldrich	P9416
Water	Sigma Aldrich	W4502
10× Buffer Tango	Thermo Fisher Scientific	BY5
50× Denhardt’s solution	Thermo Fisher Scientific	750018
AccuPrime Pfx Polymerase (2.5 U/ul)	Thermo Fisher Scientific	12344032
ATP	Thermo Fisher Scientific	R0441
dNTP Mix	Thermo Fisher Scientific	R1121
Dyna MyOne Streptavidin C1 beads	Thermo Fisher Scientific	65002
FastAP (1 U/μL)	Thermo Fisher Scientific	EF0651
GeneAmp 10× PCR Gold Buffer	Thermo Fisher Scientific	4379874
Human Cot-I DNA	Thermo Fisher Scientific	15279011
Klenow Fragment (10 U/mL)	Thermo Fisher Scientific	EP0052
Maxima Probe qPCR 2xMM	Thermo Fisher Scientific	K0233
Maxima SYBR Green kit	Thermo Fisher Scientific	K0251
Maxima SYBR Green kit	Thermo Fisher Scientific	K0253
Salmon sperm DNA	Thermo Fisher Scientific	15632-011
SSC Buffer (20 ×)	Thermo Fisher Scientific	AM9770
T4 DNA Ligase	Thermo Fisher Scientific	EL0012
T4 DNA Ligase, HC (30U/μL)	Thermo Fisher Scientific	EL0013
T4 DNA Polymerase	Thermo Fisher Scientific	EP0062
T4 Polynucleotide Kinase	Thermo Fisher Scientific	EK0032
2× HI-RPM hybridization buffer	Agilent Technologies	5190-0403
2% Sodium Hypochlorite Solution	Millipore Sigma	Cat# XX0637-76
Acetic Acid, Glacial (TraceMetal Grade)	Fisher Chemical	Cat# A507-P212
7 M HNO_3_ (Optima)	Fisher Chemical	Cat# A467-2
6 M HCL (TraceMetal Grade)	Fisher Chemical	Cat# A508-4
0.05 M HNO_3_ (Optima)	Fisher Chemical	Cat# A467-2
30% H_2_O_2_ (GR ACS Grade)	Millipore Sigma	Cat# HX0635-2
0.1 M CH_3_COOH (GR ACS Grade)	Millipore Sigma	Cat# AX0073-6
Critical commercial assays		
MinElute PCR Purification Kit	QIAGEN	28006
NextSeq^®^ 500/550 High Output Kit v2 (150 cycles)	Illumina	FC-404-2002
HiSeq X Reagent Kits	Illumina	FC-501-2501
Deposited data		
Raw and analyzed data from 33 newly reported ancient humans	This paper	ENA: PRJEB53475
A stable isotope analysis of 20 ancient samples reported in this study	This paper	IsoBank. ID: 686
Human Origins genotype data	Reich Lab website	https://reich.hms.harvard.edu/allen-ancient-dna-resource-aadr-downloadable-genotypes-present-day-and-ancient-dna-data
Western and Eastern Ashkenazi Jews	Behar et al. (2013)	http://www.evolutsioon.ut.ee/MAIT/jew_data/
Ashkenazi Genome Consortium sequencing data	Carmi et al. (2014a); Lencz et al. (2018)	https://ega-archive.org/studies/EGAS00001000664
Italy Imperial, Late Antiquity and Medieval sequencing data	Antonio et al. (2019)	https://reich.hms.harvard.edu/allen-ancient-dna-resource-aadr-downloadable-genotypes-present-day-and-ancient-dna-data
Canaanites SNP enrichment	Agranat-Tamir et al. (2020)	https://reich.hms.harvard.edu/allen-ancient-dna-resource-aadr-downloadable-genotypes-present-day-and-ancient-dna-data
Germany Early Medieval sequencing data	Veeramah et al. (2018)	https://reich.hms.harvard.edu/allen-ancient-dna-resource-aadr-downloadable-genotypes-present-day-and-ancient-dna-data
Hungary Langobard SNP enrichment and sequencing data	Amorim et al. (2018)	https://reich.hms.harvard.edu/allen-ancient-dna-resource-aadr-downloadable-genotypes-present-day-and-ancient-dna-data
Denmark Viking sequencing data	Margaryan et al. (2020)	https://reich.hms.harvard.edu/allen-ancient-dna-resource-aadr-downloadable-genotypes-present-day-and-ancient-dna-data
Software and algorithms		
OxCal 4.4	Bronk Ramsey (2009)	https://c14.arch.ox.ac.uk/oxcal.html
BWA version 0.7.15	Li and Durbin (2009)	https://bio-bwa.sourceforge.net/
contamMix version 1.0-12	Fu et al. (2013)	https://github.com/DReichLab/ADNA-Tools
ANGSD	Korneliussen et al. (2014)	http://www.popgen.dk/angsd/index.php/ANGSD
HaploGrep2	Weissensteiner et al. (2016)	https://haplogrep.i-med.ac.at/
MALT	Herbig et al. (2016); Vagene et al. (2018)	https://uni-tuebingen.de/fakultaeten/mathematisch-naturwissenschaftliche-fakultaet/fachbereiche/informatik/lehrstuehle/algorithms-in-bioinformatics/software/malt/
READ	Monroy Kuhn et al. (2018)	https://bitbucket.org/tguenther/read
smartPCA	Patterson et al. (2006)	https://reich.hms.harvard.edu/software
ADMIXTOOLS version 5.1	Patterson et al. (2012)	https://reich.hms.harvard.edu/software
Samtools	Li (2011)	http://www.htslib.org/download/
BEAST 2 v2.6.6	Bouckaert et al. (2019)	https://www.beast2.org/
hapROH version 0.1a8	Ringbauer et al. (2021)	https://pypi.org/project/hapROH/
bcftools/ROH	Narasimhan et al. (2016)	https://samtools.github.io/bcftools/howtos/roh-calling.html
IBDseq	Browning and Browning (2013)	https://faculty.washington.edu/browning/ibdseq.html
PHCPImpute	This paper	https://github.com/ShamamW/PHCPImpute (DOI: https://doi.org/10.5281/zenodo.7233296)
GLIMPSE v1.0.0	Rubinacci et al. (2021)	https://odelaneau.github.io/GLIMPSE/
ADMIXTURE version 1.3.0	Alexander et al. (2009)	http://dalexander.github.io/admixture/download.html
DATES	Chintalapati et al. (2022)	https://github.com/MoorjaniLab/DATES_v3600
HOPS	Hubler et al. (2019)	https://github.com/rhuebler/HOPS
Other		
Sr-Spec Resin (50–100 μm)	Eichrom Technologies	Cat# Sr-B25-S
gnomAD	Karczewski et al. (2020)	https://gnomad.broadinstitute.org/

## Highlights

- Genome-wide data for 33 Jewish individuals from 14^th^-century Erfurt, Germany
- Medieval and modern Ashkenazi Jews (AJ) have similar ancestral genetic sources
- Medieval AJ were genetically heterogeneous, likely divided into two or more groups
- The individuals descend from an extreme founder event shared with modern AJ
