# Supplementary material for: Genome-wide data from medieval German Jews show that the Ashkenazi founder event pre-dated the 14th century
Source: Cell. Author manuscript; Available in PMC 2022 Dec 27. (PMC9793425; doi:10.1016/j.cell.2022.11.002)
Supplement: MMC1 [file NIHMS1852590-supplement-MMC1.pdf]

Cell, Volume *185*

Supplemental information

Genome-wide data from medieval German Jews show that the Ashkenazi founder event pre-dated the 14th century

Shamam Waldman, Daniel Backenroth, Éadaoin Harney, Stefan Flohr, Nadia C. Neff, Gina M. Buckley, Hila Fridman, Ali Akbari, Nadin Rohland, Swapan Mallick, Iñigo Olalde, Leo Cooper, Ariel Lomes, Joshua Lipson, Jorge Cano Nistal, Jin Yu, Nir Barzilai, Inga Peter, Gil Atzmon, Harry Ostrer, Todd Lencz, Yosef E. Maruvka, Maike Lämmerhirt, Alexander Beider, Leonard V. Rutgers, Virginie Renson, Keith M. Prufer, Stephan Schiffels, Harald Ringbauer, Karin Sczech, Shai Carmi, and David Reich

Data S1: Supplementary information: historical background, archeological details, supplementary methods, additional statistical analyses, and supplementary discussion. Related to STAR Methods.

Section 1. Early Ashkenazi Jewish history and previous genetic studies

1. The origins of early Ashkenazi Jews

There are currently two main competing (although not entirely mutually exclusive) historical theories to explain Ashkenazi Jewish early origins. The first holds that AJ are at least partially descendants of Roman-period Diaspora Jews. This theory is supported by dispersed historical and archaeological evidence along the Germanic frontiers of the late Roman Empire. On the basis of the results of the Cologne synagogue excavations — a building that the excavator controversially dates to the early Carolingian period — it was argued that there is direct demographic continuity between the scattered late Roman Jewish "proto-Ashkenazic" presence in the region and the Jewish communities of the Rhineland of later times [1, 2].

The second theory, which is supported by more historical, onomastic, and linguistic data, regards AJ as a purely medieval formation that did not arise until the 10th century. According to this theory, AJ communities initially arose in the form of just a handful of family groupings in a few episcopal and royal urban centers and were the descendants of Jews from Southern Europe. There was continuous Jewish presence in Southern Europe since Roman times, and an extensive network of intercommunal ties linked these Jewish communities economically, culturally, and demographically to other Jewish communities around the Mediterranean [3-6]. Research suggests that early AJ of Northern Europe were the recipients of Jewish liturgical, legal, mystical, and linguistic practices from medieval Southern Italy.

The available historical evidence does not support a third hypothesis that early AJ were primarily descendants of early medieval non-Jewish converts to Judaism known as Khazars — a polyethnic tribal constellation then resident in the Caucasus and adjacent regions [6].

2. Previous genetic studies of substructure in Ashkenazi Jews

A number of previous studies have searched for genetic patterns of substructure in Ashkenazi Jews. Gusev et al. [7] analyzed genome-wide data from about 400 Ashkenazi Jewish individuals from Israel and about 300 from New York. They did not find any difference in the distribution of IBD segment lengths within or between the groups (Figures 3 and 4A therein). Guha et al. [8] studied about 1300 Ashkenazi volunteers from Israel recruited from blood banks. They showed all AJ individuals on a PCA plot colored by country of origin (Figure 6 therein) and did not observe any pattern indicating the existence of structure. The study of Kopelman et al. [9] included about 160 AJ samples from multiple studies, not overlapping the two mentioned above. Their MDS and *ADMIXTURE* analyses on AJ individuals from different countries of origin did not show any visible structure (Figure 4E and 5A therein). Finally, Privé et al. [10] was able to identify about 1700 UK Biobank participants of Ashkenazi ancestry based on their overlap in PC space with reference Ashkenazi samples from other studies. These results suggest the absence of major population structure in Ashkenazi Jews, regardless of the present country of residence and the pre-WWII geographic origin.

Three studies used genome-wide data to search for subtler evidence of population structure by comparing AJ of Western and Eastern European origin. Behar et al. [11] studied about 200 Jewish individuals from various Jewish communities in Israel, including 16 AJ of Eastern European origin and 13 of Western European origin. In an *ADMIXTURE* analysis, they found a minor component (≈2%) of Central/East Asia-related ancestry in AJ of Eastern European, but not Western European origin (Figure 3 therein). Granot-Hershkovitz et al. [12] studied about 900 Jewish individuals from Kibbutzim in Israel. Among individuals with a documented country of birth, 42 AJ individuals were born in Eastern Europe

and 47 in Germany (a proxy for Western Europe). A PCA plot (Supplementary Figure 3 therein) showed that the distributions of Western and Eastern AJ were highly overlapping, yet slightly shifted from one another. They also found higher levels of IBD sharing within Western AJ. Gladstein and Hammer [13] studied 239 Eastern AJ and 19 Western AJ (partly overlapping with [11]), finding that these groups were not fully overlapping in PCA and *ADMIXTURE* analyses (Figures S10 and S11 therein), and that a model of Western/Eastern substructure was more likely than a single population model. They dated the divergence between the two groups to 15 generations ago and inferred a higher growth rate for Eastern AJ (Table 1 therein) and more genetic drift in Western AJ (Figures S12-S14 therein).

We also note that AncestryDNA found that its AJ users could be divided into three sub-clusters, but was not able to assign genealogical or geographical interpretation to these clusters [14] (pages 2-3 in their supplementary). Finally, a number of earlier studies found geographic differences within AJ in the allele frequencies of specific loci, e.g., mtDNA [15, 16], HLA genes [17], or specific pathogenic variants [18, 19].

Section 2. The medieval Erfurt Jewish community

1. <u>Historical background</u>

The medieval Jewish community in Erfurt was the oldest in Thuringia, and existed between the late 11th century to 1454. The Erfurt old synagogue is the oldest (partly) intact synagogue in Europe [20]. The community practiced rabbinical Jewish law [21]. Erfurt belonged to the territory of the archbishop of Mainz, but was surrounded by territories of different counts and nobles. The Jews in surrounding towns were also part of the Erfurt community and buried their deceased in Erfurt [22, 23]. In the second half of 13th century, several families from the region of Franconia (in today's Northern Bavaria) immigrated to Erfurt and probably to other towns in Thuringia. By the 14th century, about 30 families or more lived in Erfurt [23].

In 1349, a wave of pogroms (massacres) occurred, and many Jews in Erfurt and other towns in Thuringia were murdered [24-26]. Like in other cities with resident Jewish communities, in Erfurt, too, anti-Jewish persecutions started even before the arrival of the Black Death in 1350 [26]. Some families, particularly the wealthy ones, survived in territories in the region where pogroms did not occur, and could even keep parts of their property. It is unknown whether these families lived in Erfurt or in nearby towns before 1349, but in 1354, they belonged to those who resettled in Erfurt [25].

After 1354, the newly founded, "second" community of Erfurt grew to become one of the largest Jewish communities in Germany [27]. As the lists of rentals show, about 50 Jewish families lived in Erfurt by the 1370s. The rapid increase in the population between the 1350s and the 1370s was due in substantial part to migration of several Jewish families from Bohemia, Moravia, and Silesia to Erfurt and nearby towns (see Section 2.2) [28]. Surrounding Jewish settlements were part of the Erfurt community and buried their deceased in Erfurt after 1354 as well [29].

Some families left Erfurt in the 1380s and 1390s, whereas after 1400, families from nearby towns moved into Erfurt. The number of Jews in Erfurt after 1407 is unknown, as no lists of rentals remained [22, 23]; but it is known that in 1418, at least 20 families lived in Jewish settlements in the region that was part of the Erfurt community [29]. During the 1430s and 1440s, Jews in some areas of Thuringia were expelled or were forced to leave, and a few moved to Erfurt [29]. In 1453, the city council of Erfurt no longer granted the protection of the Jews. The Jewish families left Erfurt within a year,

marking the end the medieval Jewish community [30]. Resettlement of Jewish individuals only occurred in the 19th century in a different part of the city.

2. Documented migration from the East into the second Erfurt community

The information on the origin of Jewish families who migrated to Erfurt comes mainly from records of home rentals from 1354 to 1407. Most persons in these records are mentioned with bynames, which often name the town where they lived before [28]. Information in topographic bynames is limited, as they can change, and as the time period when a person has lived in the other town could vary. But in some cases, we have independent sources validating the former place of residence. From 1354, and especially in the 1360s, many families moved to Erfurt whose bynames refer to former places of residence in Bohemia, Moravia, and Silesia. For example, several families came from Breslau (Wrocław) after a pogrom in 1360, some after moving to Wrocław from other Silesian towns. After 1400, there are no known cases of families migrating into Erfurt from the East [28, 29].

Towns in Silesia (present-day Poland) from where families moved to Erfurt include Bunzlau/Bolesławiec (one family, first mentioned in the records in 1383), Liegnitz/Legnica (two related families in 1360), Löwenberg/Lwówek Śląski (one person whose family was originally from Brno), Breslau/Wrocław (one family in 1355/6, more families after 1360), Striegau/Strzegom (one family in 1366), Schweidnitz/Świdńica (one person in 1389), and Glatz/Kłodzko (one family in 1380). Towns in Bohemia and Moravia (present-day Czech Republic) from where families moved into Erfurt include the neighboring towns Braunau/Broumov and Náchod (two families in 1360 or later who moved through Wrocław), Prag/Praha (one family in 1366), Pilsen/Plzeň (one family in 1365), Eger/Cheb (one family in 1359), and Brünn/Brno (one family in 1363, with a son-in-law in Vienna) [28]. One man is known to have moved to Erfurt from Poland in 1327 (i.e., in the first community).

Section 3. The archaeological excavation

1. Ethics

Traditional Judaism imposes very strict regulations on the management of Jewish cemeteries, including the directive that the dead should be left in peace—relocation is possible only under very particular circumstances. As a result, archaeological investigations in areas where Jewish cemeteries have survived (or are suspected to exist) are not allowed by traditional Jewish law as a matter of principle. While we know of quite a few medieval Jewish cemeteries in Europe, only few have been excavated [1, 31]. All published examples are the result of rescue excavations. In most cases, the human bones were reburied as quickly as possible, in consultation with the Jewish communities. The excavations in Erfurt took place under similar circumstances.

2. The cemetery

The medieval Erfurt Jewish cemetery was located, following religious regulations, outside the city of Erfurt itself. Its location today is confidently known based on 14th-century sources [26]. It is unknown when the excavated section of the cemetery was used for burial by the Erfurt Jewish community. However, some hints arise from examining the fortifications around the site and from archaeological evidence from elsewhere in the city. Our excavated section is located between the first city wall (12th century) to the south and an outer wall to the north, in an area where a moat used to lie in front of the first city wall (Figure 1A in the main text; Figure 1 below). It is conceivable that the excavated section was used as a cemetery only after the construction of the outer wall, as prior to constructing that wall, the area was used for fortification and the original cemetery must have extended beyond

the outer wall to the north. In Brühl, a site in the western part of Erfurt, wood retrieved from the moat in front of the first city wall was dated by dendrochronology to 1324/1325. Several years later – at an unknown date – the moat was filled and a second fortification wall was built with a new moat in front of it. It is plausible that the construction of an outer wall in the Jewish cemetery and in Brühl happened at around the same time. We consequently hypothesize that the outer wall in the area of the Jewish cemetery was constructed only in the second half of the 14th century. If correct, this would imply that the excavated section of the cemetery was used only by the second community. Radiocarbon dating of the teeth we sampled indicated dates primarily in the 14th century and definitely not later (Data S2, Table 1; Data S2, Table 3). However, the radiocarbon results could not exclude origins in the first half of the 14th century or even slightly earlier, which would place the samples in the first community (Figure S1C; Figure 2 below). After the expulsion of the Jews from Erfurt in 1454, a barn and a granary were built by the city in the years 1465-1473 on top of the cemetery. The granary (Kornhofspeicher) still exists today. The southern and northern walls of the granary were constructed on top of the inner and outer city walls, respectively (Figure 1 below).

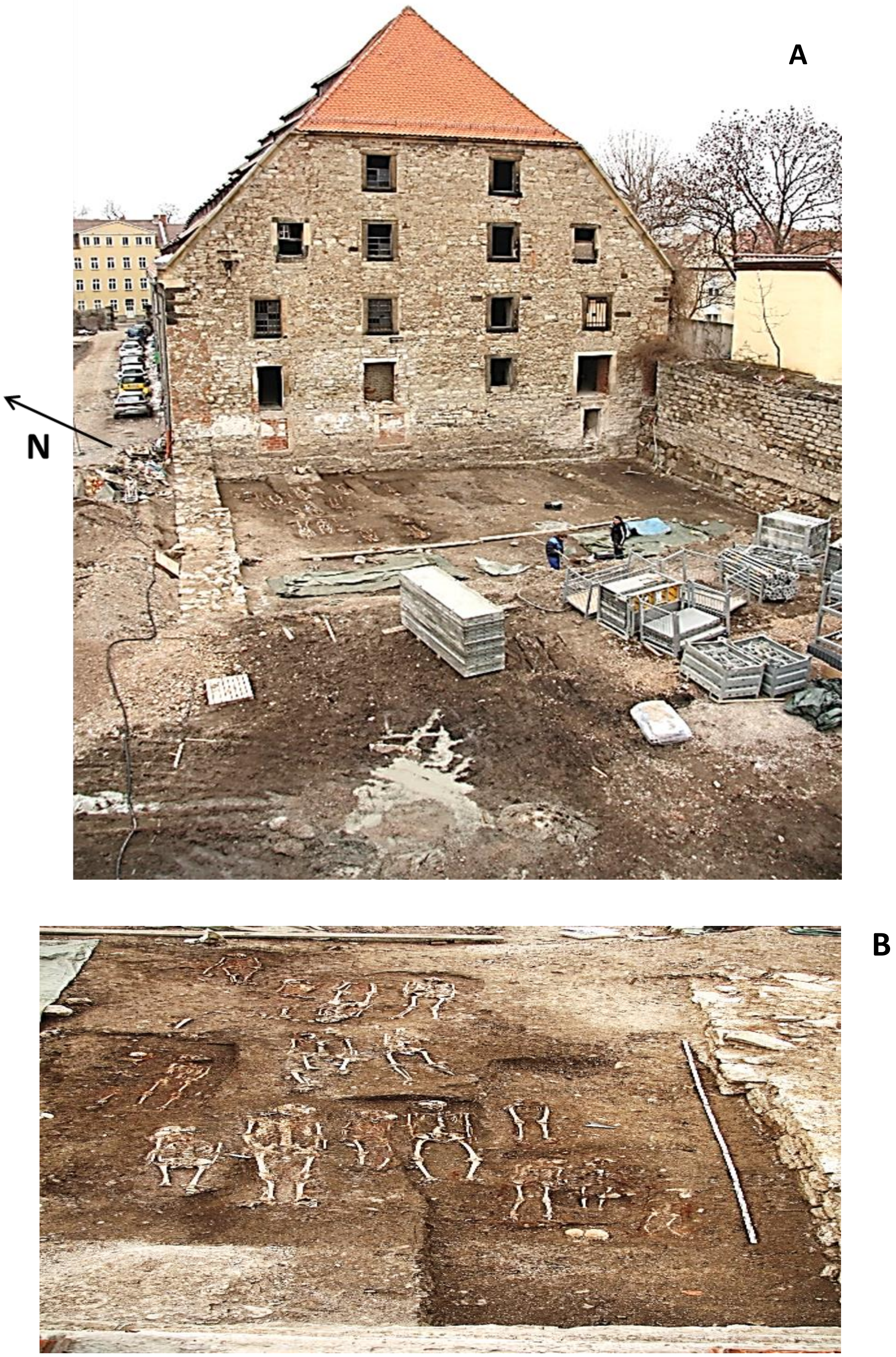

Figure 1. The archeological site. (A) The excavation of the medieval Jewish cemetery in Erfurt, Germany. The arrow points in the approximate direction of the north. The large structure behind (to the east of) the excavation site is a granary (the "Kornhofspeicher") that was built in the 15th century on top of the cemetery and now serves as a garage. Behind the granary is Moritzstraße (distant building to the left), which delimits the area of the original cemetery to the east. To the right of the site is the wall of the old town of Erfurt, which bounded the

cemetery from the south. To the left (north) is an outer city wall that was built later. The area between the walls underwent salvage excavations before the construction of a ramp in 2013. The cemetery likely extended further north and west beyond the area of the excavation: the main part of the cemetery was possibly north of the city's fortifications. (B) Skeletons that were discovered in the excavation (view from the granary). Both photos were adjusted for brightness and contrast.

3. The excavation

In earlier investigations of the area surrounding the granary (over a period of several years), numerous gravestones and human bones were recovered. Burials *in situ* (in graves) were only observed in the area a little further north of the outer wall. These could not be recovered due to safety reasons. The conversion of the granary into a multi-story car garage in 2013 required the construction of an external ramp, which then necessitated an archaeological rescue investigation. The excavation was carried out between 8 March and 17 April 2013. Since graves were not recognizable, a planum was first laid out by machine, at which point wood remains and some bones started to become visible. From then on, excavation was only done by hand. In the course of the excavation, at the suggestion of the builder, the recovery of the skeletons was restricted to areas where the constructions were expected to destroy the graves. In other areas, the skeletons remained in the ground.

The number of graves exposed during the excavation is likely a small fraction of the total in the cemetery. The size of the excavation area was about 16 x 12 meters. It is certain that the cemetery continued to the west, up to an unknown boundary. To the east, burials were partially destroyed by the construction of the granary. The ground level inside the granary is so low that its construction in the 15th century destroyed all burials along a length of more than 80 meters. If one assumes an overall occupancy of a similar density as in the excavation field, about 1000 graves were destroyed by the construction of the granary. This assumes burial only on one level, as was encountered in the excavation area.

4. The findings

The archaeological documentation includes 47 burials. Six further graves were documented only after construction was underway and could only be partially recovered. Remains of wooden coffins were found in almost all graves. The graves were located remarkably close to one another (Figure 1A in the main text), and followed medieval Jewish funerary practice in that the integrity of the graves is always preserved. With one exception (I14850), all the burials lay parallel to the city wall, with the legs of the interred pointing roughly to the east – i.e., roughly in the direction of Jerusalem (Figure 1A in the main text). No grave goods were observed, but there is evidence that some of the dead were buried with their clothes. This is suggested by the presence of buckles (I14904, who was violently killed), a piece of jewelry on one of the women (I14850 again; the piece has a close parallel in the treasure trove from Weißenfels from 1349), and a silk ribbon on the head of a child. A full report detailing the physical anthropology of the remains will be published in the future [32].

A full description of the site, including an excavation report, will appear in the *Die mittelalterliche jüdische Kultur in Erfurt* (volume 6).

5. Our study

The Jewish community of Thuringia approved the genetic study in 2018 under the conditions that only detached teeth are used and no excavation is performed specifically for the purpose of DNA research.

In 2018, we (K.S. and S.F.) collected detached teeth (mostly molars) for the DNA study. Overall, we found 38 teeth, one per individual (see teeth numbers in Data S2, Table 1). In 2021, all skeletons were reburied in the recently recovered Jewish cemetery of the 19th-century community, and signs explain the history of the cemetery and provide information about the reburied Medieval dead.

6. Radiocarbon dating

Radiocarbon dating of ten individuals showed that all lived between about 1270-1400 CE (Figure S1C). However, due to a wiggle in the ^{14}C calibration curve (Figure 2 below), we could not determine whether they lived before or after the 1349 pogrom. Hence, we could not determine if they belonged to the first or second Jewish communities.

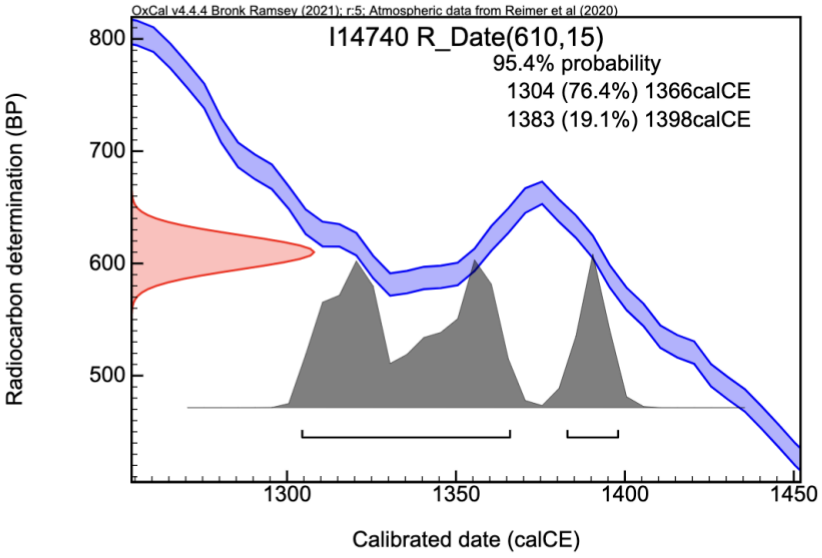

Figure 2. The radiocarbon calibration curve of a representative sample. We show a screenshot from OxCal for sample I14740 (see also Figure S1C). The figure demonstrates the wiggle in the calibration curve throughout the 14th century, which prohibits a definitive dating of the sample to before or after the 1349 pogrom.

Section 4. The DNA analysis

1. The number of covered SNPs

The DNA of Erfurt individuals was enriched for about 1.24 million SNPs. The mean and median number of covered SNPs in the autosomes of the Erfurt genomes were 402k and 383k, respectively. After merging with the Human Origins dataset, the mean and median number of SNPs in the Erfurt genomes were 219k and 205k, respectively.

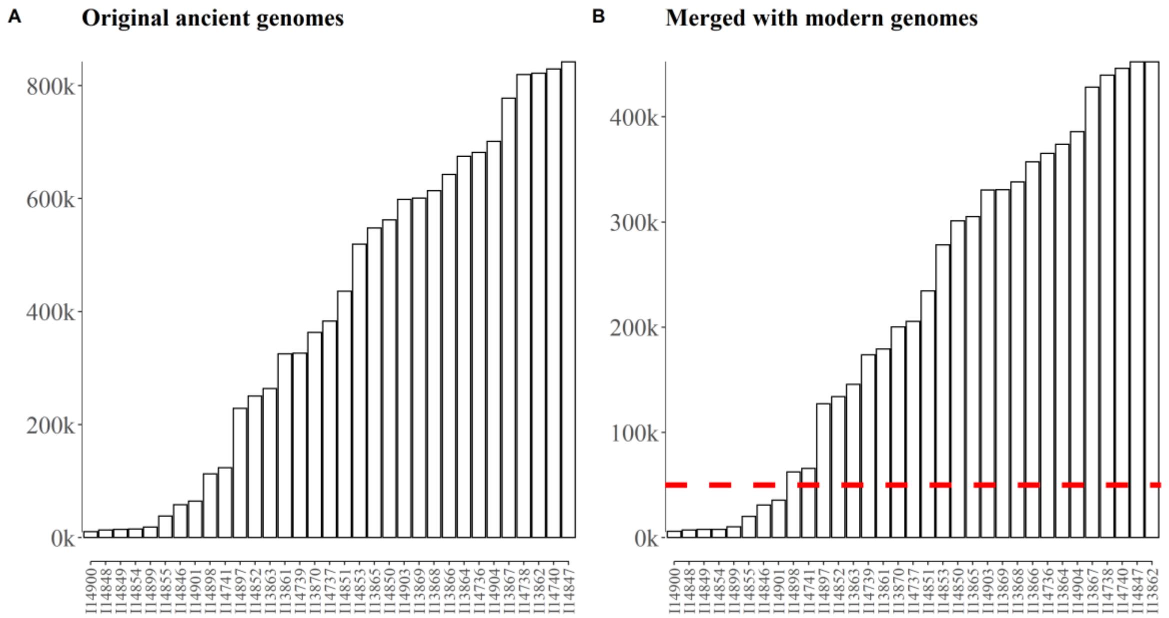

Figure 3. The number of covered SNPs in each Erfurt sample. (A) In the original ancient genomes (autosomes only). (B) After merging with the Human Origins dataset. The horizontal dashed line indicates the cutoff defining the low-coverage genomes (see Section 5 below).

2. Lower coverage in children

We observed that all EAJ genomes covered at <100k SNPs were under the age of 13. To formally test whether children had lower coverage, we used the mid-range of the estimated age at death (see STAR Methods) and classified all individuals of estimated age ≤20 as children and all others as adults. We excluded two samples whose ages were not estimated. We then used a two-tailed t-test to compare the number of covered SNPs between children and adults. The significantly lower coverage in the children ($P=6.7\cdot10^{-7}$) raises the possibility that DNA may be less well preserved (on average) in teeth that are not fully developed.

3. Pathogen DNA scan

The screening pipeline included a three-step authenticity check of the sequences that align to a pathogen: the edit distance distribution, the presence of C-to-T sequence damage, and the edit distance distribution of the sequences that contain damage. Only a single pathogen, *Enterobius vermicularis,* passed all three authenticity screening steps (Data S2, Table 4). However, *E. vermicularis*, commonly known as a pinworm, is a human intestinal parasite that has previously been sampled from ancient latrines [33]. It is therefore unlikely that this pathogen would have been present in the teeth of the Erfurt individuals at their time of death, and the most likely source of this DNA is contaminated groundwater in the Erfurt cemetery after burial. The groundwater contamination hypothesis is further supported by the presence of reads aligning to *E. vermicularis* in as many as 23/33 Erfurt individuals (Data S2, Table 4), and by the fact that most cases (21/23; Data S2, Table 4) failed tests for the authenticity of ancient DNA. Similarly, five Erfurt individuals showed weak evidence for the *Schistosoma mansoni* pathogen, another water-borne human intestinal parasite that could have been introduced in the sampled teeth via environmental contamination after death. Very weak evidence was detected for pathogens associated with periodontal disease (*Parvimonas micra*, *Fusobacterium*, and *Fusobacterium nucleatum*) in a single individual, I14850 (the mother of family A, who was also

buried in opposite orientation to all other individuals and possibly with her clothes on; Section 3). Fewer than 30 sequences aligned to each of these pathogens, and no evidence of C-to-T damage was detected in any of the aligned sequences. However, these are all common oral pathogens [34], therefore it is possible that the sequences represent authentic DNA that was present in the oral microbiome during individual I14850's lifetime, but that was not well preserved in this sample.

Overall, our pathogen screening analysis did not find convincing evidence of any pathogens of interest among the Erfurt individuals. Particularly, we found no evidence of *Yersinia pestis*, the pathogen responsible for the plague, among any of the Erfurt individuals. The lack of evidence cannot completely rule out the possibility of *Y. pestis* infection in any given individual, as the preservation rate of *Y. pestis* DNA in teeth from individuals who are known to have died from plague has previously been estimated at only 37% [35]. However, the failure to detect any evidence of this pathogen among any of the Erfurt individuals suggests that the Erfurt cemetery is unlikely to have been a mass burial site for victims of a plague epidemic.

4. Assignment of terminal Y chromosome and mitochondrial lineages

Y chromosome. We only considered the ten male Erfurt genomes with >50k covered autosomal SNPs. Initial haplogroup calls were generated as described in the STAR Methods section. We used these assignments to search for *terminal SNPs*, which define more refined branches or subclades. We defined the terminal branches using reference haplotrees from YFull (v10.05), a Y chromosome sequence interpretation service, and from FamilyTreeDNA (FTDNA), a direct-to-consumer genetic testing company. For each Erfurt genome, we used the haplogroup assignment to initiate the search. We then manually compared the Erfurt genome sequence to downstream or nearby haplotree levels where modern AJ are commonly found. We repeated the search until finding a match with a terminal SNP. For each putative terminal branch, we validated the absence of variants that define downstream branches. Modern AJ Y sequences were available to us (L. R. C. and J. L.) as project administrators at FTNDA. The study received the approval of FTDNA. We called the genotype in each relevant SNP in each Erfurt genome manually using the BAM file in the Integrative Genomics Viewer (version 2.8.2). We converted SNP IDs to hg19 coordinates using https://www.genetichomeland.com/welcome/dnamarkerindex.asp. Across all individuals, only in two SNPs multiple alleles were present, and we called the genotype in these SNPs based on the majority of the reads. The method was effective in establishing a terminal branch for all ten genomes. The terminal branches and the nearest associated AJ lineage are listed in Data S2, Table 1.

Mitochondrial DNA. We used YFull's MTree (v1.02.17740) to define the reference haplotrees. As for Y, we started with the initial haplogroup call. For each Erfurt genome, we searched the mitochondrial DNA sequence for SNPs downstream of the initial haplogroup to define terminal branches.

Section 5. Ancestry estimation

1. <u>The minimal number of SNPs for PCA</u>

To determine the minimal number of SNPs for a reliable PC projection, we down-sampled seven high-coverage EAJ (four Erfurt-EU and three Erfurt-ME) to different levels of coverage (5k, 10k, 30k, 50k, and 100k SNPs) and examined their location in PC space relative to the original samples. For each genome and for each coverage level, we generated 20 down-sampled copies, and projected them onto the West-Eurasian PC space, as in the main text.

The results (Figure 4 below) show that starting from 50k SNPs, the down-sampled genomes remain reasonably close to their original positions and there is no overlap between genomes originally designated as Erfurt-EU or Erfurt-ME. Hence, in downstream analyses of the PCA results, we only used genomes covered by at least 50k SNPs.

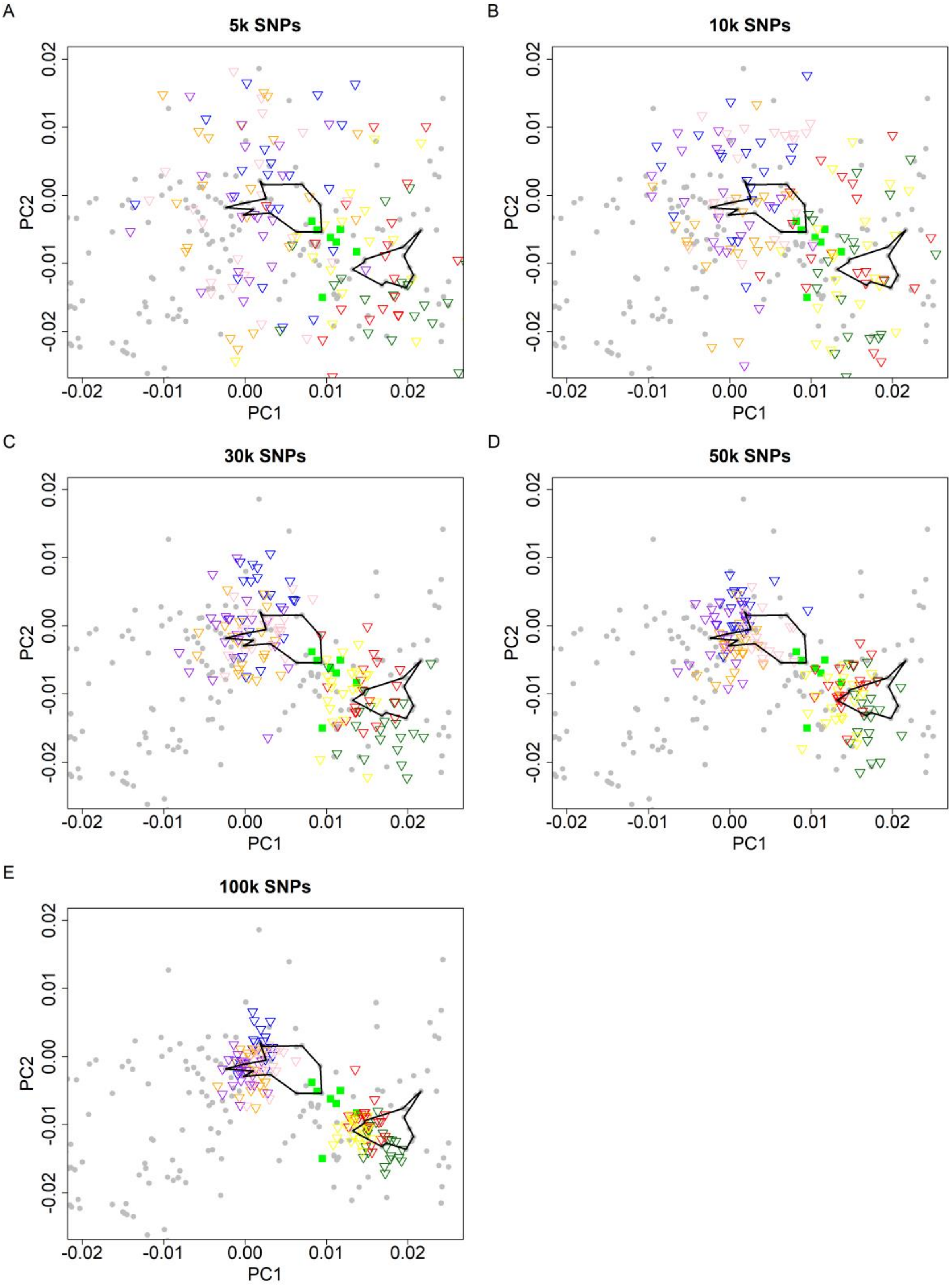

Figure 4. The effect of the coverage of the Erfurt genomes on the PCA results. Each panel represents different coverage level with 20 down-sampled copies for each original sample. The down-sampled genomes are plotted as triangles, colored based on their original sample ID. MAJ are plotted as filled green squares, and the original EAJ genomes are marked by two polygons corresponding to Erfurt-EU (to the left of MAJ) and Erfurt-ME (to the right of MAJ). All PCA plots are zoomed-in versions of the PCA in the main text.

2. <u>PCA with AJ of Western and Eastern European origin</u>

For the analyses of MAJ of Eastern European vs Western European origin, we merged the EAJ genomes with those from [11]. The merged dataset included 245,792 autosomal SNPs. The following modern populations from [11] dataset were used to learn the PCs: Abkhasian, Adygei, Algerian_Jewish, Armenian, Balkar, Bedouin, Belarusian, Bulgarian, Chechen, Croat, Cypriot, Druze, Estonian, French, French_Basque, Georgian, Georgian_Jewish, Greek, Hungarian, Iranian, Iranian_Jewish, Iraqi_Jewish, Italian, Jordanian, Kumyk, Lebanese, Lezgin, Libyan_Jewish, Lithuanian, Mordovian, Moroccan, Moroccan_Jewish, North_Ossetian, Orcadian, Palestinian, Russian, Saudi, Sephardi_Jewish, Spanish, Syrian, Tunisian_Jewish, Turkish, Polish, and Ukranian. The total resulting sample size was $n = 882$. We projected the Erfurt and MAJ (Ashkenazi_Jewish_Eastern and Ashkenazi_Jewish_Western) genomes on the resulting PC space. Due to the smaller number of SNPs after merging the datasets, three additional EAJ genomes had fewer than 50k SNPs and were excluded from the PC analysis.

We designated AJ individuals as being Western European if they had origins in France, Netherlands, or Germany. We designated AJ individuals as being Eastern European if they had origins in Russia, Belarus, Lithuania, Latvia, Poland, Romania, or Austria-Hungary.

3. <u>Estimating genetic ancestry using *ADMIXTURE*</u>

We ran *ADMIXTURE* version 1.3.0 [36] using default parameters and using only SNPs that were covered in at least 18 Erfurt genomes (about 86k SNPs). We used the populations included in the PCA and the following populations: Erfurt, Egyptian, Han, Hazara, Kalash, Mbuti, Mandenka, Yoruba, Pima, China_Lahu, She, Adygei, Oromo, Somali, Dinka, Mala, Saami_WGA, Burbur_WGA, Ain_Touta_WGA, Azeri_WGA, Shaigi_WGA, Kurd_WGA, Assyrian_WGA, Naro, Shua, Nogai, Altaian, Dolgan, Tajik, Turkmen, Luo, Savo, Tunisian, Jew_Ethiopian, Algerian, Mansi, Jew_Cochin, Turkish_Balikesir, Saharawi, Irish, Moroccan, German, and Yemeni. Similarly to the PCA, individuals with fewer than 50k SNPs were not included in the *ADMIXTURE* analysis. We ran ADMIXTURE with an increasing number of ancestral populations, starting from K=4. At K=10, the Erfurt individuals were modeled as having a unique ancestry component, and we thus show results for $4 \leq K \leq 9$. Each run terminated whenever the change in the log-likelihood was under 10^{-4}. We visually inspected the ancestry profiles generated by *ADMIXTURE* using different seeds (for K=4 and K=7) to verify that convergence was reached.

The *ADMIXTURE* results demonstrated that EAJ are genetically similar to MAJ (and South-Italians), but with higher variance (Figure 5 below), consistent with the PCA findings. Individuals classified based on the PCA as Erfurt-EU had higher EU-related ancestry. The results also revealed a small but consistent East-Asian-related component, especially in the Erfurt-EU group (means of 2.7% and 1.6% in Erfurt-EU and all EAJ, respectively, for K=7), as previously observed [11]. This suggests either a minor gene flow event from East-Asia, as previously attested by mtDNA [37], or gene flow from Eastern European populations, who carry (at least today) a minor component of this ancestry (Figure 5 below). See also Section 7.5.

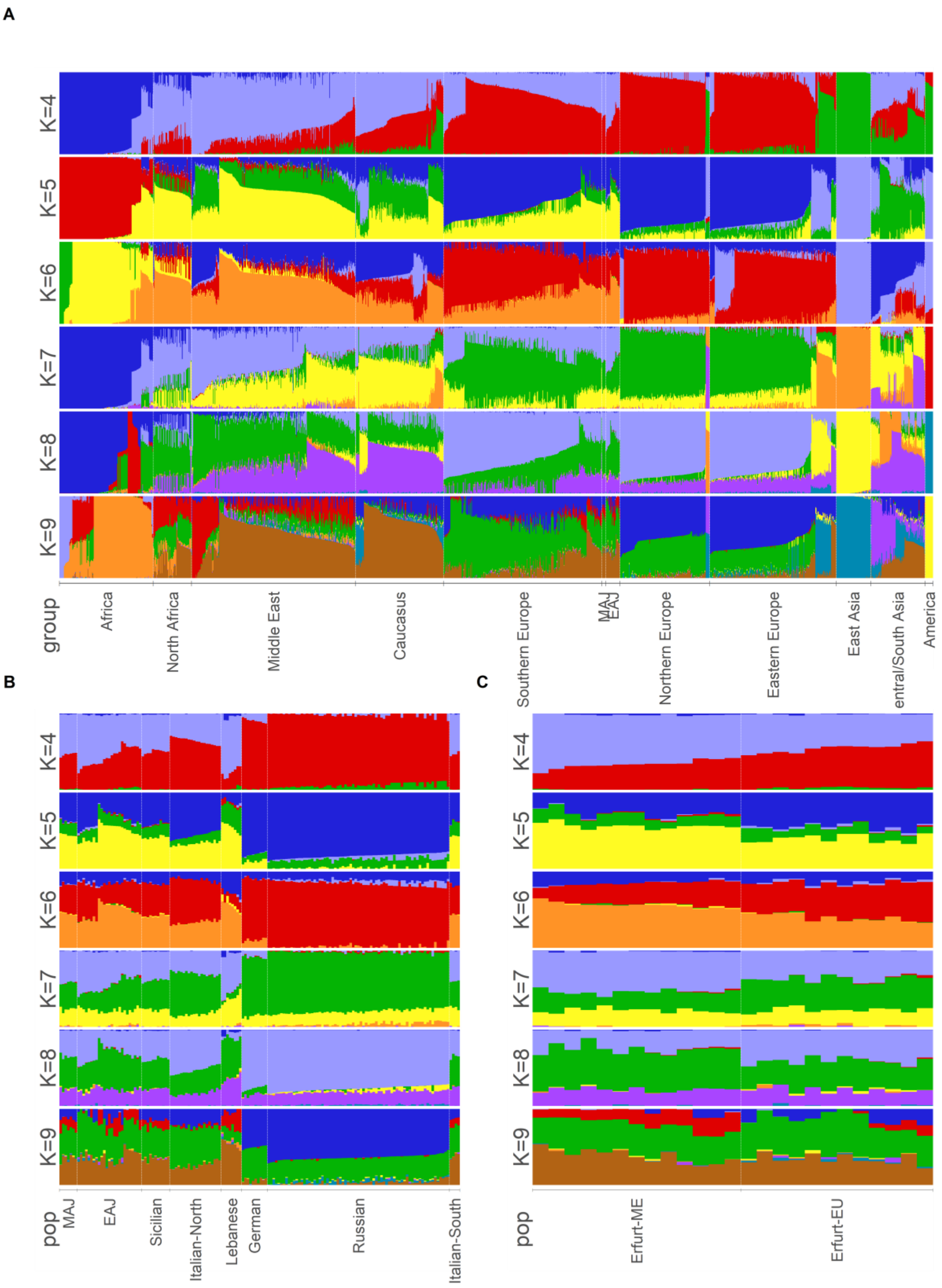

Figure 5. *ADMIXTURE* results. (A) Results for all populations, grouped by regions, for K =4,5,6,7,8, 9 ancestral components. We also show a zoom-in on populations relevant for our study (B) and EAJ alone (C). In (C), we divided EAJ into Erfurt-EU and Erfurt-ME and sorted the samples by the European component (red) in $K = 4$.

Section 6. Evidence for the presence of two Erfurt sub-groups

1. <u>The gap statistic</u>

The gap statistic method [38] identifies the number of clusters that best fit the data given a clustering method and a range of possible numbers of clusters. We used the function fviz_nbclust() from the *factoextra* package in *R*. We used *K*-means to cluster the samples (“kmeans” option with nstart = 25) based on the first two PCs. We set the maximal number of clusters to $K = 4$ and the number of bootstrap samples to 500. The low-coverage samples were not included in this analysis. The optimal number of clusters was $K = 2$, providing statistical support for the existence of two sub-groups. As a control, we determined the number of clusters in modern AJ and in Moroccan Jews (from the Human Origins dataset), either separately or jointly, with the results as expected (Figure 6 below).

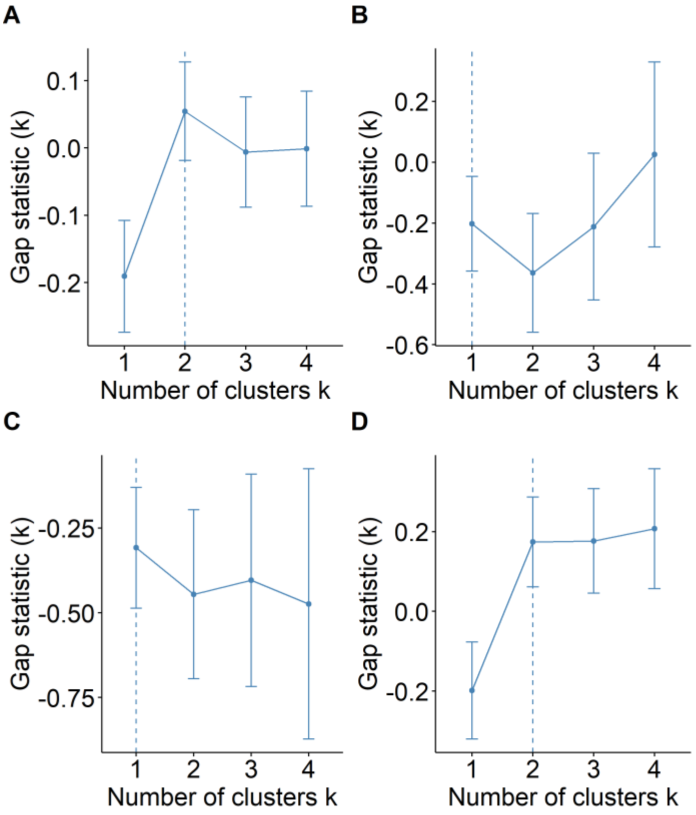

Figure 6. The number of clusters in EAJ based on the first two PCs. We used the gap statistic to infer the optimal number of clusters (dashed line) for EAJ (A). As a control, we also inferred for the optimal number of clusters for modern AJ ((B); one cluster expected); Moroccan Jews ((C); one cluster expected); and modern AJ and Moroccan Jews together ((D); two clusters expected).

2. <u>A significance test for the difference between the EAJ clusters</u>

The clustering generated by *K*-means with $K = 2$ corresponds to our Erfurt-EU and Erfurt-ME groups. We used the function test_cluster_approx() from the *clusterpval* package in *R* [39] to calculate the P-value for the difference in means between those two clusters. The number of importance samples ("ndraws") was 10,000. The difference between the two EAJ clusters was statistically significant (P=0.007).

3. <u>Robustness of the clustering</u>

To test the robustness of the clustering of the EAJ individuals, we used two additional methods to cluster the EAJ individuals into two groups. First, we used f_3(EAJ1, EAJ2; Yoruba) as a metric for the similarity between a pair of EAJ genomes, representing the genetic drift shared between them relative to an outgroup. We computed the f_3 statistics with *ADMIXTOOLS* version 5.1 with default settings. We then defined the distance between the genomes as 1-f_3. We finally used hierarchical clustering (*hclust* function in *R*) to cluster the EAJ genomes based on this distance. The two clusters generated by the first split in the tree are identical to the (PCA-based) Erfurt-EU/Erfurt-ME clusters (Figure 7 below).

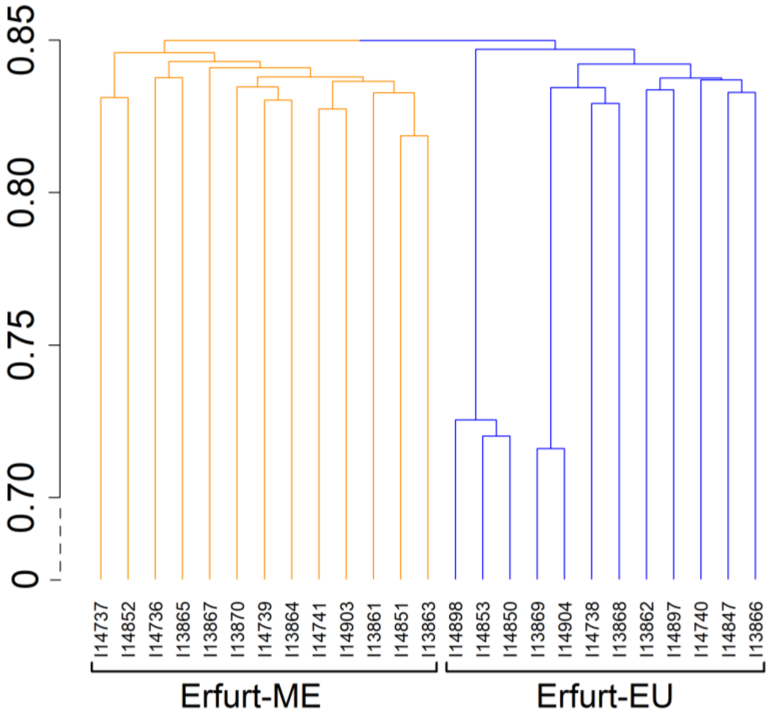

Figure 7. Clustering based on f_3 statistics. The distance metric we used between genomes EAJ1 and EAJ2 was f_3(EAJ1, EAJ2; Yoruba). The blue and orange colors represent clustering based on the first split in the tree. The orange and blue clusters are identical to Erfurt-ME and Erfurt-EU, respectively. Low-coverage genomes were not included in the analysis. The late-splitting branches correspond to Families A and B.

We next used a similar clustering approach, now defining the distance between genomes as the number of mismatching autosomal SNPs normalized by the number of SNPs covered in both genomes. Note that as these genomes are pseudo-haploid, their alleles can either match or mismatch. We then ran hierarchical clustering using *hclust* in *R*. Here too, the first split in the tree generated clustering identical to the Erfurt-EU/Erfurt-ME assignment (Figure 8 below).

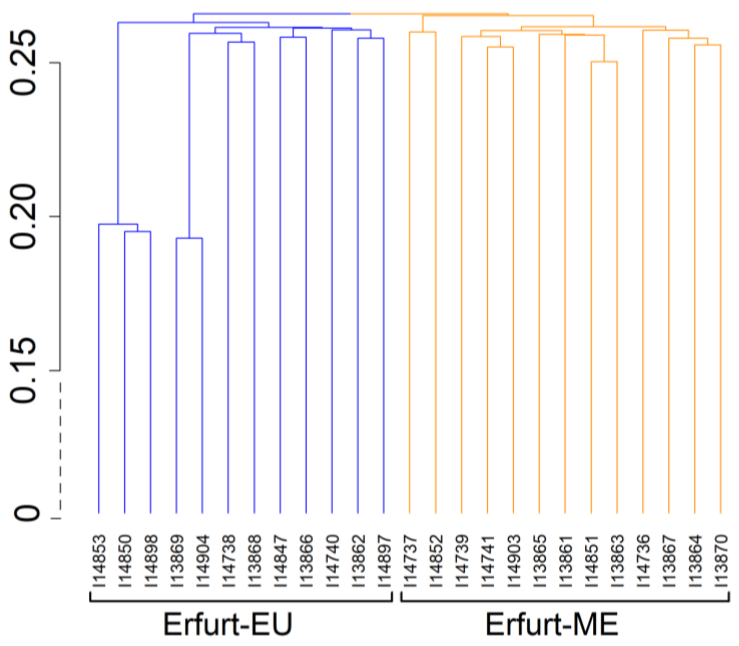

Figure 8. Clustering based on allele sharing. The distance metric we used for each pair of EAJ genomes was the proportion of mismatching SNPs. The blue and orange colors represent clustering based on the first split in the tree. The orange and blue clusters are identical to Erfurt-ME and Erfurt-EU, respectively. Low-coverage genomes were not included in the analysis. The late-splitting branches correspond to Families A and B.

4. <u>Dependence of the group assignment on the coverage</u>

The Erfurt-EU individuals had higher coverage than the Erfurt-ME individuals (Figure 9 below). This raises the concern that placement of individuals in PC space is coverage-dependent. However, our down-sampling experiments demonstrated that coverage did not affect the Erfurt sub-group assignment (Section 5 above). We also verified that there is no correlation between the proportion of European ancestry and the type of library preparation (Figure 9 below).

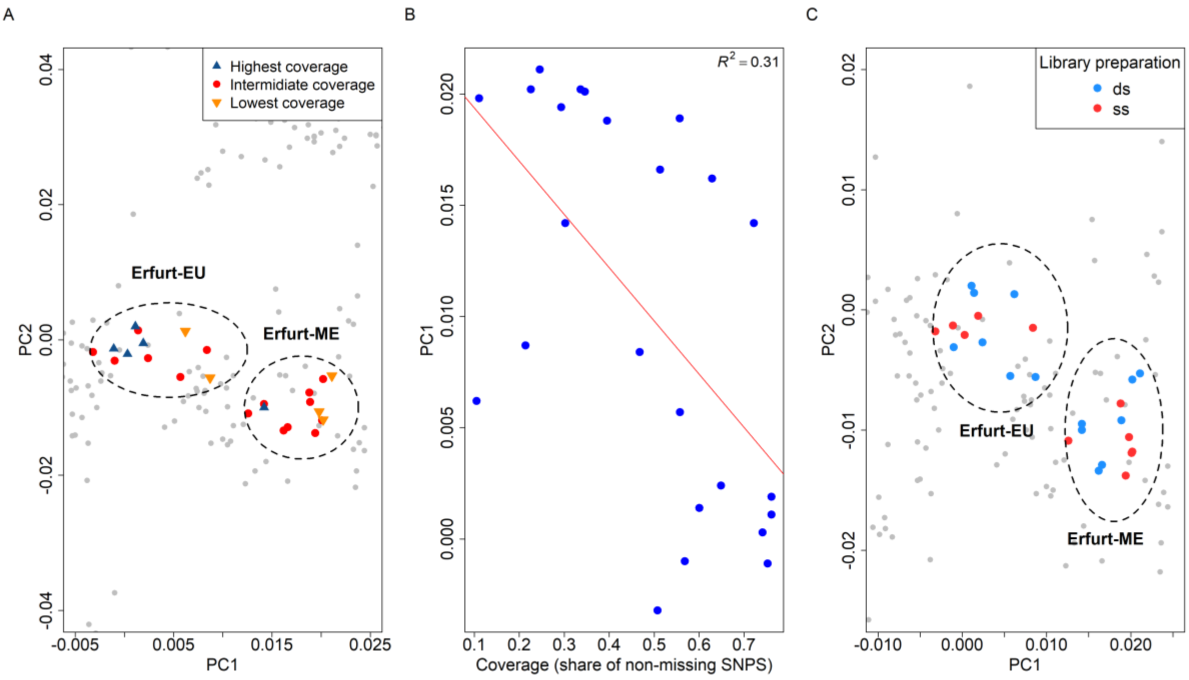

Figure 9. The association of the sequencing coverage with the PCA placement. (A) The figure shows a zoomed-in version of the PCA of the main text, where Erfurt samples are labeled by their coverage. The five individuals with the highest and lowest coverage are marked as "highest coverage" and "lowest coverage". [The PCA plot does not include the eight samples with <50k SNPs. In other words, the "lowest coverage" samples are the lowest only among the samples that were used in the PCA.] Four of the five highest coverage samples are part of the Erfurt-EU group. (B) The PC1 coordinate vs the coverage level, along with a regression line. (C) A zoomed-in version of the PCA of the main text with EAJ samples marked by their type of library preparation (ss: single-stranded; ds: double-stranded). The difference in PC1 coordinates between the two treatments was not significant (P=0.95, two-tailed t-test).

5. Simulations of substructure

To study the genetic composition of EAJ from a population genetics perspective, we simulated demographic scenarios with or without substructure. We then tested the similarity between summary statistics of the real Erfurt data and either simulated scenario. In the first scenario, there was a single admixture event between Middle Eastern (50%), Southern European (35%), and Eastern European (15%) sources (based on the model of ref. [40]) that happened five generations prior to sampling (Figure 10 below).

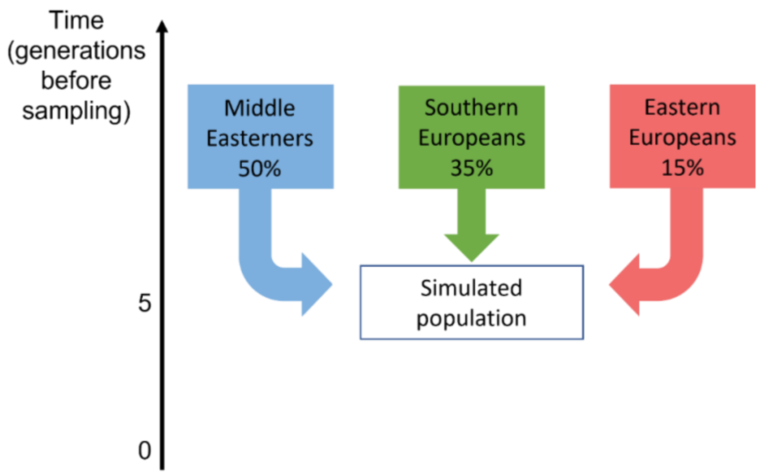

Figure 10. A schematic of an admixture model representing a single EAJ group. Under the model, the EAJ population experienced a 3-way admixture five generations prior to sampling.

In the second scenario, we simulated two groups. Both groups experienced an admixture event ten generations prior to sampling between Middle Eastern (45%) and Southern European (55%) sources. One of the groups experienced a second admixture event with Eastern Europeans (15%) five generations prior to sampling (Figure 11 below).

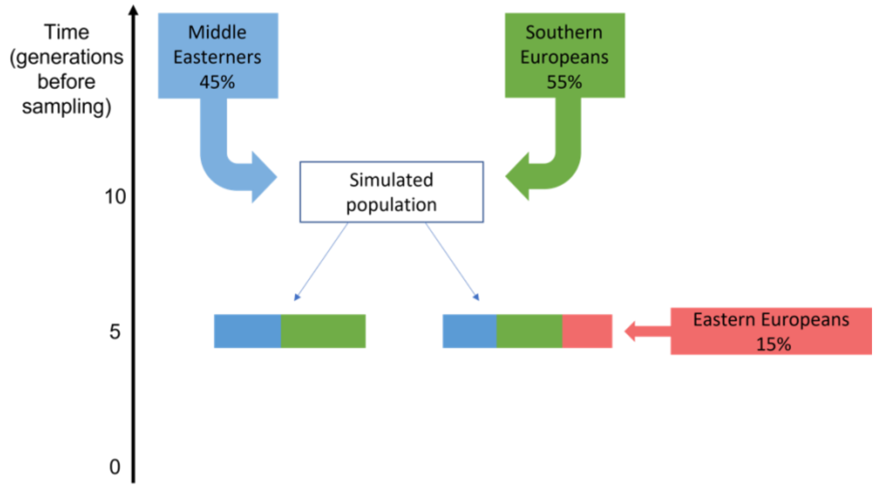

Figure 11. A schematic of an admixture model representing two EAJ groups. In this model, the population experienced an admixture event between Southern Europeans and Middle Easterners ten generations prior to sampling. The population then split in two, and one group experienced an additional admixture event with Eastern Europeans, five generations prior to sampling.

We emphasize that the two scenarios we simulated do not exhaustively cover the space of possible models for the EAJ demographic history. Rather, they were selected as representatives of models with or without an admixture event into an EAJ subgroup.

We generated the simulated genomes as follows. Each of the three sources included several populations, as listed in Table 1 below. We phased the source genomes using the Sanger Imputation Service (https://www.sanger.ac.uk/tool/sanger-imputation-service) [41] with the Haplotype

Reference Consortium reference panel. To simulate the admixture events, we followed the method described in [42]. For the simulation of a single admixture event, we randomly selected ten individuals from each of the three sources. We simulated the genomes as mosaics of haplotypes along the 22 autosomal chromosomes. We randomly assigned the source of each segment based on the simulated admixture proportions. We drew the length in cM of each segment at random from an exponential distribution with rate $G/100$, where G is the time of the admixture event in generations prior to sampling. We generated diploid genomes by pairing two simulated haploid genomes. For the scenario of two groups we simulated a single admixture event between Southern Europeans and Middle Easterners in a similar way for the first group and two admixture events in the second group. To simulate two admixture events, we first randomly selected ten individuals from Southern European sources and ten individuals from Middle Eastern sources and generated simulated diploid genomes with this admixture only. We then then used the simulated genomes and ten individuals from the East-EU source to simulate the second admixture event in a similar way. We simulated 30 genomes for the first demographic scenario (a single group), and 20 genomes for each group in the second (two-group) scenario.

Middle Eastern populations	Palestinians, Lebanese, Jordanians, Syrians, Egyptians, Bedouin A, Bedouin B, Saudis, Druze
Southern European populations	North-Italians, Greeks
Eastern European populations	Belarusians, Lithuanians, Ukrainians, Russians

Table 1. A list of the populations that were used as sources in the admixture analyses. All genomes were from the Human Origins dataset.

We ran PCA on each simulated dataset and used the Kolmogorov-Smirnov test (*ks.test* in *R*) to compare the PC1 distribution between the real data and the simulations (Figure 12 below). The distribution of PC1 coordinates was similar between the EAJ genomes and that simulated under the two-group scenario (Figure 12D below; P=0.19). The corresponding distribution under the single group scenario was different from that of EAJ (Figure 12B below; P=0.03).To validate that coverage does not affect the results, we ran PCA on pseudo-haploid down-sampled genomes from the two-group simulation. We matched the number of genomes and the number of SNPs of the (non-low-coverage) Erfurt-ME and Erfurt-EU samples. The results (Figure 12E below) showed no qualitative difference in the PCA plot compared to the full genomes.

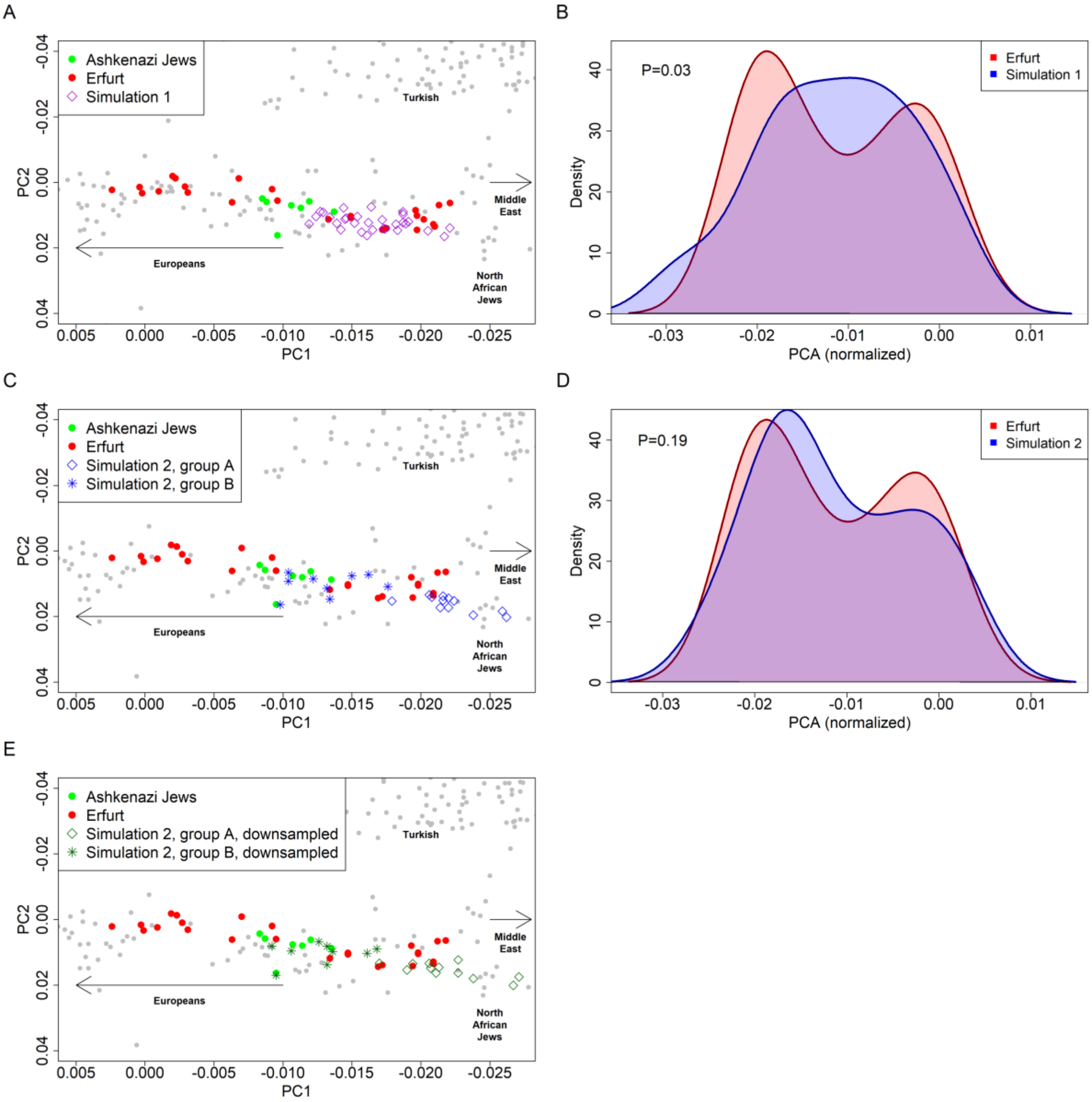

Figure 12. PCA of genomes simulated under two admixture scenarios and a comparison to EAJ. (A) PCA results for simulations of a single group that has experienced recent admixture between Middle Eastern, Southern European, and Eastern European sources. Both simulated genomes and EAJ genomes were projected on the PC plane. The grey dots correspond to modern populations from the Human Origins dataset (see labels). (B) A comparison between the distributions of PC1 of the simulated genomes (from (A)) and EAJ. The distribution of the simulated data was shifted and scaled to match the mean and variance of the EAJ data. The EAJ distribution is bimodal and does not fit the simulated data. (C) PCA results for simulations of two groups, one that has experienced admixture between Middle Eastern and Southern European sources, and one that had additional admixture with Eastern Europeans. (D) A comparison between the distributions of PC1 of the simulated genomes (from (C)) and EAJ. The distribution of the simulated data was shifted and scaled to match the mean and variance of the EAJ data. Here, the distribution of the simulated data is also bimodal. (E) PCA results for the two-group simulation after down-sampling the simulated genomes to match the coverage of the Erfurt samples. The results are qualitatively similar to those of (C).

We then ran *qpAdm* modeling on the simulated datasets with Southern European, Middle Eastern, and Eastern European sources (South-Italians, Lebanese, and Russians, respectively). When *qpAdm*

inferred a negative East-EU ancestry proportion, we used the *qpAdm*-reported Middle Eastern and Southern European ancestry proportions that would be expected had the East-EU ancestry proportion been set to zero. We used permutation testing to compare the proportion of individuals without Eastern European ancestry as inferred by *qpAdm* (Figure 13 below) between the real and simulated data. In each permutation, we pooled the samples of Erfurt-EU (11 samples), Erfurt-ME (13 samples), and the simulation (30 or 40 genomes). We then randomly labelled 24 samples as "Erfurt" and the remaining as "simulated", and computed the difference in the proportion of individuals without East-EU ancestry between the two sets. The P-value was the fraction of permutations (out of 10k) in which the absolute value of the difference was greater than in the real data. The proportion of individuals without (*qpAdm*-inferred) East-EU ancestry in EAJ was similar to that simulated under the two-group scenario (Figure 13D below; P=0.79) but not under the single group scenario (P=0.02).

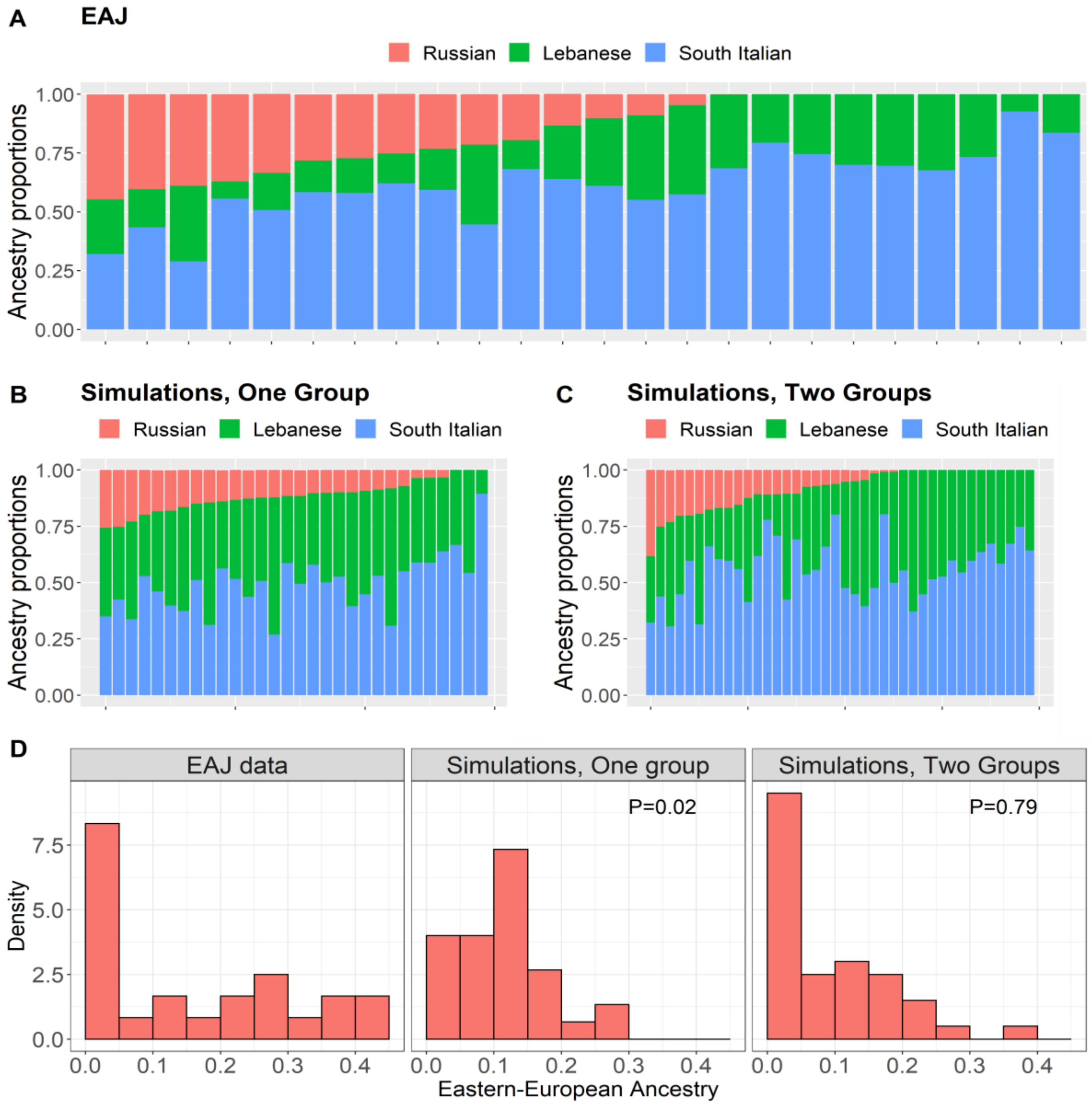

Figure 13. *qpAdm* results for genomes simulated under two admixture scenarios and a comparison to EAJ. (A) *qpAdm* results for the real Erfurt data with Lebanese, South-Italians, and Russians as sources. This panel is identical to Figure 3B of the main text. (B) *qpAdm* results for the single-group simulations. (C) *qpAdm* results for two-group simulations. (D) The distribution of the Eastern European ancestry proportions in the real EAJ data,

the single-group simulations, and the two-group simulations. The proportion of individuals with no East-EU ancestry in the real EAJ data is significantly different from that of the single-group simulation (P=0.02; permutation test, randomly shuffling the labels of simulated and real data points), but not from that for the two-group simulation (P=0.79).

Section 7. Ancestry modeling: robustness and additional models

1. Robustness of the ancestry models

To validate the robustness of our *qpAdm* models, we repeated the *qpAdm* analyses described in the main text with the following changes. First, we used all SNPs instead of only transversions (Figure 14A below). Second, we used the Ami population as the outgroup (the first population of the “right” populations in the *qpAdm* analysis) instead of Mbuti (Figure 14B below). The models that we presented in Figure 3A in the main text are models with P>0.05 in both the main analysis and the robustness tests.

Next, we tested a model for the ancestry of single individuals with the same Middle Eastern and Eastern European sources (Lebanese and Russian, respectively) as in the main text and North-Italians instead of South-Italians as the Southern European source. As in the main analysis, Erfurt-EU individuals have a substantial East-EU component that is missing from most Erfurt-ME individuals (Figure 14C below). We finally sought to determine whether the absence of Eastern European ancestry in some Erfurt-ME individuals might be due to their lower coverage (Figure 14D below). We used five high-coverage samples (two Erfurt-ME and three Erfurt-EU) and down-sampled each genome 20 times to 100k random SNPs, as in Figure 4 in Section 5 above. Two samples from Figure 4 were not used: one had no East-EU ancestry, and one could not be modeled using the given sources. We used all SNPs and the same sources as in Figure 3B of the main text: South-Italians, Lebanese, and Russians. The results show that the inferred proportion of East-EU ancestry is reasonably robust to down-sampling.

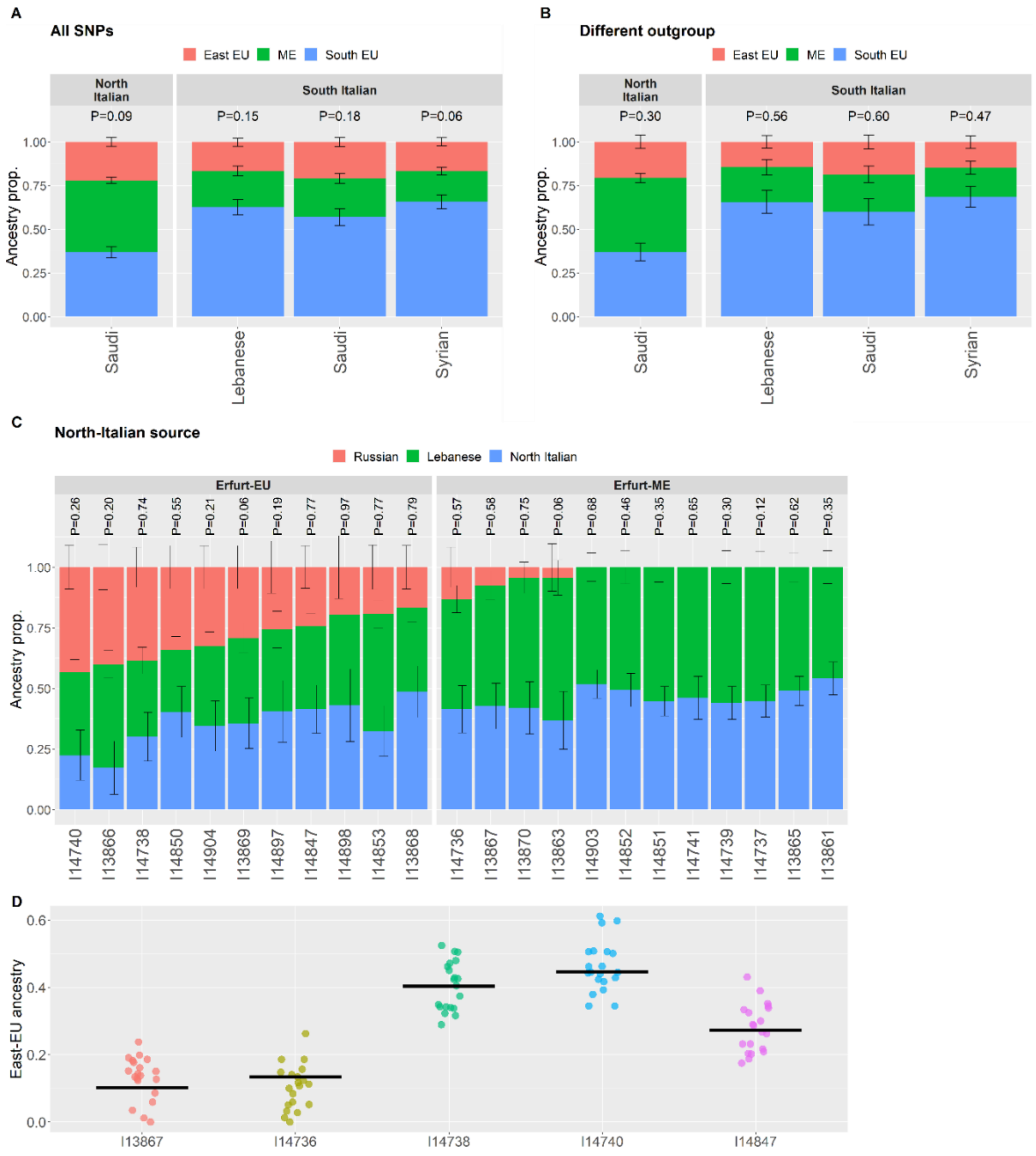

Figure 14. Robustness tests for *qpAdm*. Panels (A) and (B) are the same as in Figure 3A of the main text, showing models for the ancestry of EAJ, with the following changes. In (A), we used all SNPs instead of only transversions. In (B), we used the Ami population as the outgroup instead of Mbuti. The plots present the models that were plausible in the main analysis. Panel (C), which is analogous to Figure 3B of the main text, shows a model for the ancestry of single individuals (labeled by their IDs). The sources were Russians, Lebanese, and North-Italians (instead of South-Italians in the main text). Two individuals could not be modeled using these sources and are not presented. The individual-level models were estimated using all SNPs. (D) East-EU ancestry proportion inferred by *qpAdm* in the original EAJ samples (horizontal black lines) compared to those inferred in their down-sampled versions (colored dots).

2. Southern Mediterranean sources

We tested additional *qpAdm* models where we replaced South-Italians with other Mediterranean sources. All models included three source populations: the Mediterranean source, a Middle Eastern source (Druze, Egyptians, Bedouins, Palestinians, Lebanese, Jordanians, Syrians, or Saudi), and Russians. As in the main analysis, we only used transversion SNPs.

When we used a North-Italian source, two models, with Lebanese and Saudi Middle Eastern sources, were plausible (P>0.05), but only the model with Saudis was also plausible in the robustness tests (Table S3). When we used a Greek source, several models were plausible, but none of them was plausible in the robustness tests (Table S3). When we used Spanish, all models were implausible, and the highest p-value was 0.01 (using Druze as the Middle Eastern source). When we used a North-African source, all P values were close to 0.

We also merged all Middle Eastern sources that generated plausible models in the *qpAdm* analysis of the main text (Lebanese, Saudis, and Syrians) together with North-Italians, South-Italians, and Greeks into a single Mediterranean/Middle East source. We then tested a model with two sources, Mediterranean/Middle Eastern and Russian. This model was plausible using transversions (P=0.11), although less probable when using all SNPs (P=0.03).

3. Middle-Eastern sources

We tested *qpAdm* models where instead of using one of the Middle Eastern populations as a source, we merged all Levant populations (Palestinian, Jordanian, Druze, Bedouin A, Bedouin B, Syrian, Lebanese) into a single source. These models had three source populations: Southern European (North-Italians, South Italians, or Greeks), Levant, and Eastern Europeans (Russians). We only used transversion SNPs.

The model was plausible when we used South-Italians (P=0.07) but not when we used North-Italians or Greeks (Table S3). When we used all SNPs instead of transversions, the model with South-Italians was not plausible (Table S3). The results were similar when we added Cypriots to the Levant source (Table S3).

4. A Western European source instead of the Russian source

Our *qpAdm* models suggested that Erfurt, or at least Erfurt-EU samples, have a substantial Eastern European ancestry component. To test if the Eastern European component can be replaced with a Western European one, we repeated the *qpAdm* analysis, but replacing the Russian source with Germans. The other sources were different Southern European and Middle Eastern populations, as in the main analysis. We used transversions SNPs and Mbuti as the outgroup population.

All the tested models were implausible (P<0.05; Table 2 below), indicating that the Eastern European source cannot be replaced by a Western European one.

**Middle Eastern source**	**South-EU source**		
	**North Italian**	**South Italian**	**Greek**
**Druze**	2.8e-5	0.0025	9.6e-6
**Egyptian**	4.5e-24	9.5e-10	3.2e-7
**Bedouin A**	4.7e-17	7.7e-8	9.3e-6
**Bedouin B**	1.3e-6	0.00038	0.00093

**Palestinian**	2.3e-11	1.5e-5	8.1e-5
**Lebanese**	0.0058	0.020	0.0051
**Jordanian**	2.8e-07	0.00049	0.0020
**Syrian**	0.00012	0.0027	0.0011
**Saudi**	0.0024	0.0030	0.0058
**Levant**	9.1e-11	9.1e-6	5.4e-5
**Levant + Cyprus**	3.3e-10	1.5e-5	7.0e-5

Table 2. *qpAdm* models with a Western European source. In all models, EAJ was the target group and there were three source populations: Middle Eastern, Southern European, and Western European. The rows represent the Middle Eastern source in each model and the columns represent the Southern European source. We used Germans as the Western European source. Models with P>0.01 are highlighted in light green. No model has P>0.05.

5. East Asian ancestry

The *ADMIXTURE* analysis suggested a minor ancestry component in AJ that may be attributed to East Asia. To evaluate the potential contribution of East Asians to the ancestry of EAJ, we tested models where the sources were Lebanese, South-Italians or North-Italians, Russians, and Han Chinese (Han were dropped from the reference populations for this analysis). The models had P-values of $1.9 \cdot 10^{-10}$ and $1.8 \cdot 10^{-6}$ with South- and North-Italians, respectively. When the target was Erfurt-EU, the P-values were $7.5 \cdot 10^{-8}$ and $1.8 \cdot 10^{-4}$, respectively. Given that the same models for EAJ without Han had plausible P-values (Table S3), this analysis supports no major East-Asian ancestry in EAJ. On the other hand, one individual (I14740) carried the mtDNA terminal haplogroup N9a3a1b1, which is nested within a Central/East Asian branch (https://www.yfull.com/mtree/N9a3a1/).

6. Relations between EAJ, MAJ, and other Jewish groups

To quantify the difference in Eastern European ancestry between MAJ and Erfurt-ME, we used *qpAdm* to model MAJ as the target of admixture between Erfurt-ME and Russians. We only used transversion SNPs. The model was plausible with P=0.76, with ancestry proportions 87% for Erfurt-ME and 13% for Russians. The model was plausible also with Germans as a source instead of Russians (P=0.74; ancestry proportions 86% for Erfurt-ME and 14% for Germans).

To quantify the relation between Erfurt-ME and Sephardi Jews, we used *qpAdm* to model Erfurt-ME using Turkish Jews and Germans as sources. We again used only transversion SNPs. The model was plausible with P=0.96, with ancestry proportions 97% for Turkish Jews and 3% for Germans. A model with Russians instead of Germans was also plausible (P=0.96; ancestry proportions 96% for Turkish Jews and 4% for Russians). Finally, we were able to model modern AJ with Erfurt-ME as Erfurt-EU as sources (ancestry 60% and 40%, respectively; P=0.74).

7. Ancient sources

Given that the true ancestral sources of EAJ were ancient, we tried to model EAJ as a mixture of ancient sources. The sources we used were Imperial or late antique Romans [43], Canaanites [44], and early medieval Germans [45]. These models gave poor fit (P<0.01 for both Roman sources), suggesting a missing ancestry component. Alternatively, the poor fit might reflect technical artifacts due to

inhomogeneous data types: the Canaanite and EAJ datasets were produced by in-solution enrichment, while the Imperial/late antique Roman and early medieval German datasets were produced by shotgun sequencing.

Section 8. Estimating admixture times

1. <u>Estimating the admixture time based on EAJ data</u>

We hypothesized that the Erfurt individuals may provide information regarding the timing of admixture in AJ due to their proximity in time to the events, and attempted to estimate the admixture times using *DATES* [46, 47]. As *DATES* cannot infer the dates of multiple admixture events, we focused on the more recent event, which likely involved Eastern Europeans. We used Erfurt-EU as the target admixed population, as most Erfurt-ME individuals lack Eastern European ancestry. We omitted one of each pair of first-degree relatives, keeping the individual with the higher coverage. The source populations were chosen only as those who were plausible sources in the *qpAdm* models, with Russians as one source and Middle Easterners and Southern Europeans as the other source (Lebanese, Syrian, Jordanian, BedouinB, Saudi, South-Italians, North-Italians, and Sicilian. We used an equal sample size (37 genomes) from each of the Middle Eastern and the Southern European sources. The other source was Russians, with 71 genomes. We used the following *DATES* parameters: binsize: 0.001; maxdis: 1; qbin: 10; and lovalfit: 0.45. The estimated admixture time was 21.2 $\pm$ 7.3 (Figure 15 below).

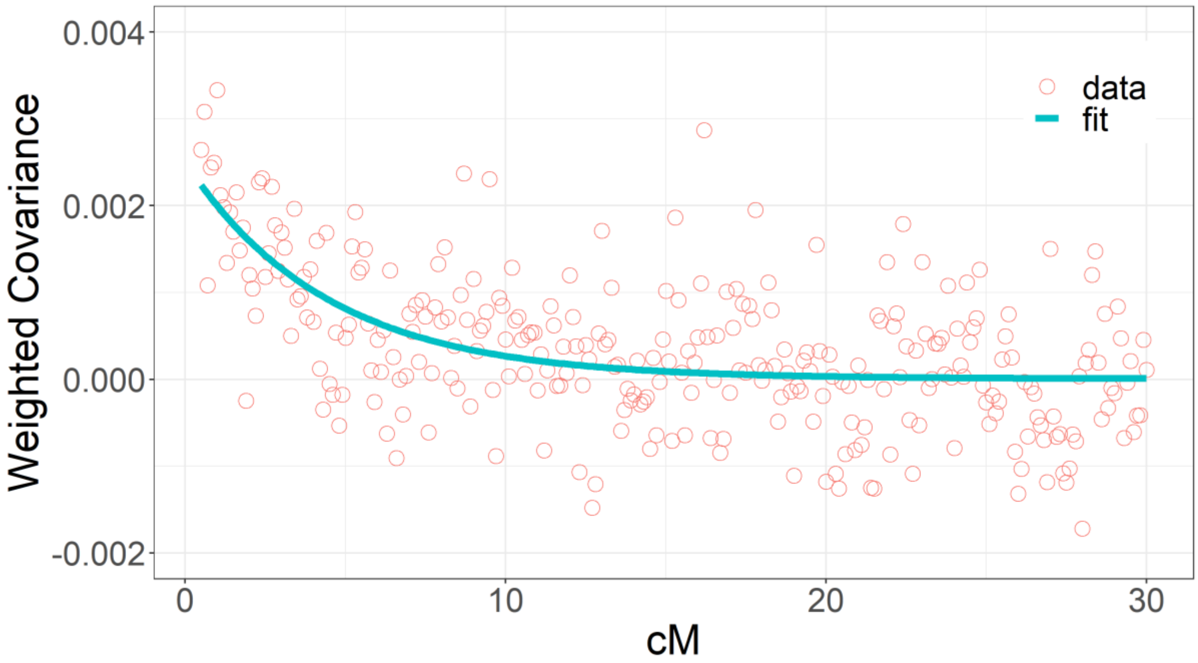

Figure 15. *DATES* results for Erfurt-EU. The coral circles represent the observed weighted covariance (described in [46-48]) between the genotypes of SNPs in each genetic distance apart. The blue line represents the least squares fit to an exponential decay.

2. <u>Estimating admixture time in simulated data</u>

We used simulations to evaluate the accuracy of *DATES*. The simulated demographic history included two admixture events: the first between Middle Eastern and Southern European sources (35% and 65% ancestry from each source, respectively), and the second with Eastern Europeans (replacing 15%

of the gene pool). We simulated two scenarios: one with the admixture events occurring 60 and 10 generations prior to sampling the target genomes, and another with events 70 and 20 generations prior to sampling. We generated the simulated genomes as described in Section 6 above, except that we added South-Italians and Sicilians to the pool of the Southern European source. All genomes used for the simulations were removed from subsequent *DATES* analyses. We simulated nine genomes, and down-sampled them to form pseudo-haploid data with coverage matching that of Erfurt-EU — the target group in the real *DATES* analysis.

We repeated each simulated scenario 50 times and analyzed the simulated genomes with *DATES*. We used a combined, balanced Middle Eastern and Southern European source, as in the real data analysis. Each of the sources included the populations listed in Table 1 in Section 6 above (with the addition of South-Italians and Sicilians to the Southern-European sources). We found that the *DATES* estimates had an upward bias and a very large variance (Figure 16 below). Hence, we conclude that *DATES* cannot reliably infer the admixture time between Middle Eastern/Southern European and Eastern European sources.

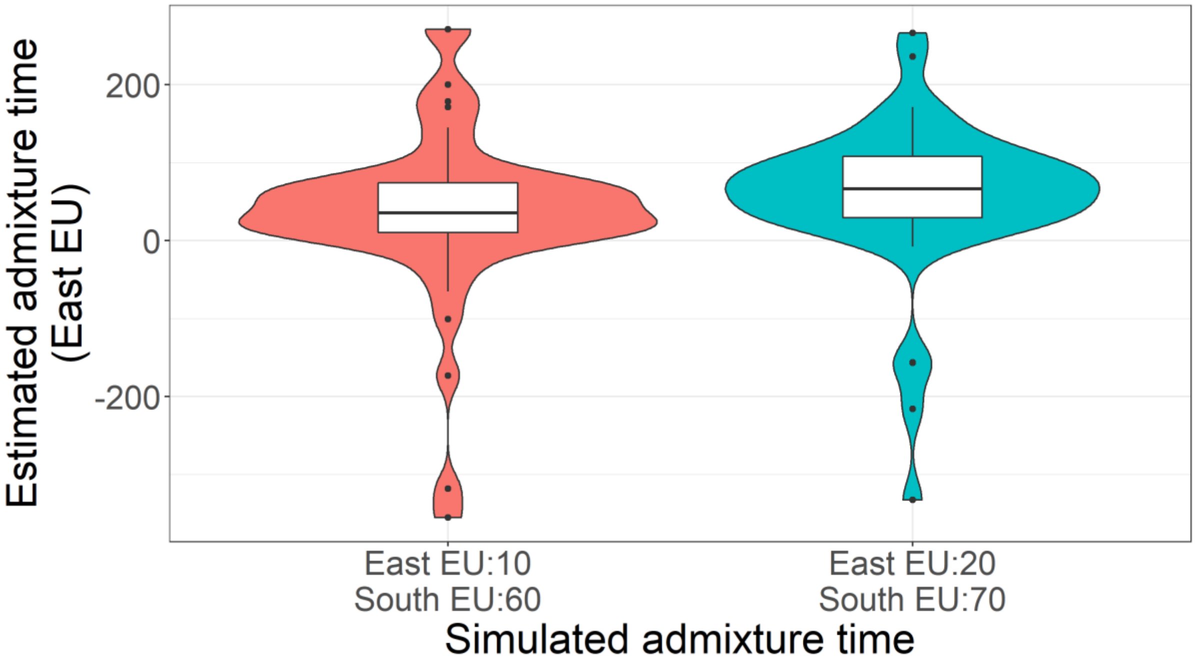

Figure 16. Simulations testing the accuracy of *DATES*. We simulated two admixture scenarios, as indicated in the x-axis labels. The y axis represents the inferred admixture times by *DATES*. The plot shows the densities of the *DATES* estimates under the two simulated scenarios. In the box plots, the bold horizontal line represents the median, the borders of the box are the first and third quartiles, and the vertical lines extend to the most extreme value no more distant than 1.5x the inter-quartile range from the quartiles. We omitted from the plot one data point for which the inferred admixture time was 1,915 generations ago. The distribution of the estimated admixture times is extremely wide under both scenarios, suggesting that *DATES* cannot reliably infer the admixture time for the real Erfurt-EU data.

Section 9. The mitochondrial DNA analysis

1. Aligning the K1a1b1a sequences of the modern and ancient samples

We noticed that all EAJ carriers had identical sequence except for a single site at position 16223. At that site, samples I13867, I13870, and I14903 had the C allele, while the remaining eight carriers had T. The same polymorphism also segregated in 107 MAJ K1a1b1a carriers (C count: 48/107). In the modern samples, beyond 16223C/T, there were 36 variants: 32 singletons, one doubleton, two variants that appeared in three samples, and one variant that appeared in four. Excluding the 16223C/T site, 76/107 MAJ carriers had an identical sequence to that of EAJ.

To determine whether MAJ carriers have significantly more diversity compared to EAJ carriers, we used down-sampling experiments. In each experiment, we sampled at random 11 MAJ carriers, and computed the number of pairwise differences (excluding site 16223). In comparison, the 11 EAJ carriers had no pairwise differences. Over 10,000 runs, the mean number of pairwise differences in MAJ carriers was 0.83 (SD: 0.44). The proportion of runs where MAJ carriers had zero pairwise differences (as in EAJ) was 1.83%. These results are expected given the longer time of the modern samples to their most recent common ancestor (TMRCA) compared to the ancient samples.

2. The *BEAST* analysis

The total effective sample size (ESS) for the TMRCA, as estimated by BEAST, was 3508. The mutation rate (across the entire mtDNA sequence) was estimated as $4.6 \cdot 10^{-8}$ per bp per year, broadly in agreement with previous estimates [49, 50], although with a relatively low ESS of 148. We show the posterior distribution of the TMRCA in Figure 17 below.

Estimates | Marginal Density | Joint-Marginal | Trace

Summary Statistic	TreeHeight
mean	2534.3609
stderr of mean	92.7218
stdev	5492.1968
variance	3.0164E7
median	1498.6651
value range	[655.2723, 2.2072E5]
geometric mean	1773.9897
95% HPD interval	[655.2723, 6701.3475]
auto-correlation time (ACT)	2.5654E5
effective sample size (ESS)	3508.2
number of samples	9000

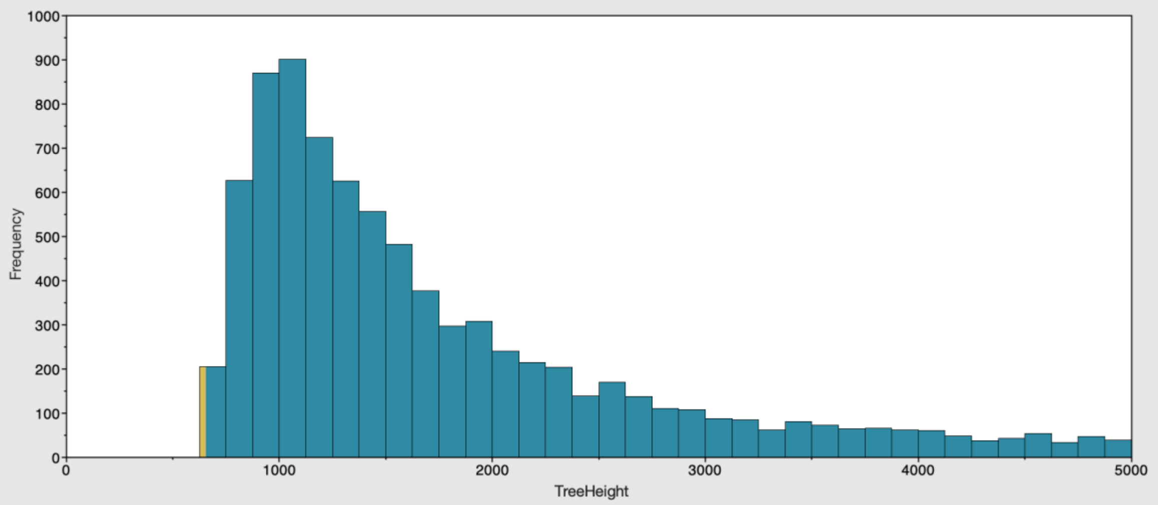

Figure 17. The posterior distribution of the mtDNA tree height (time to the most recent common ancestor (TMRCA)) based on ancient and modern K1a1b1a carriers. The plot shows a screenshot of the *Tracer* software showing the output of the *BEAST* analysis, as described in STAR Methods. Briefly, we used an alignment of the mtDNA sequence of 11 EAJ and 107 MAJ K1a1b1a carriers. We ran *BEAST* with a strict clock, Gamma distributed mutation rates, and a skyline population size prior. The effective sample size (ESS) was 3508, and the total

number of samples from the posterior was 900. The median posterior TMRCA was 1499 years ago, with a 95% highest posterior density (HPD) interval 655-6701 years ago. Other characteristics of the distribution are shown above the plot.

We present the maximum clade credibility tree based on these runs in Figure 18 below. As expected given the pattern of polymorphism in 16223, the Erfurt lineages (IDs S1XXXXMT, where X is any digit) coalesced with the modern lineages based on their genotype at 16223, and thus the TMRCA of the Erfurt samples is the same as that of the modern samples.

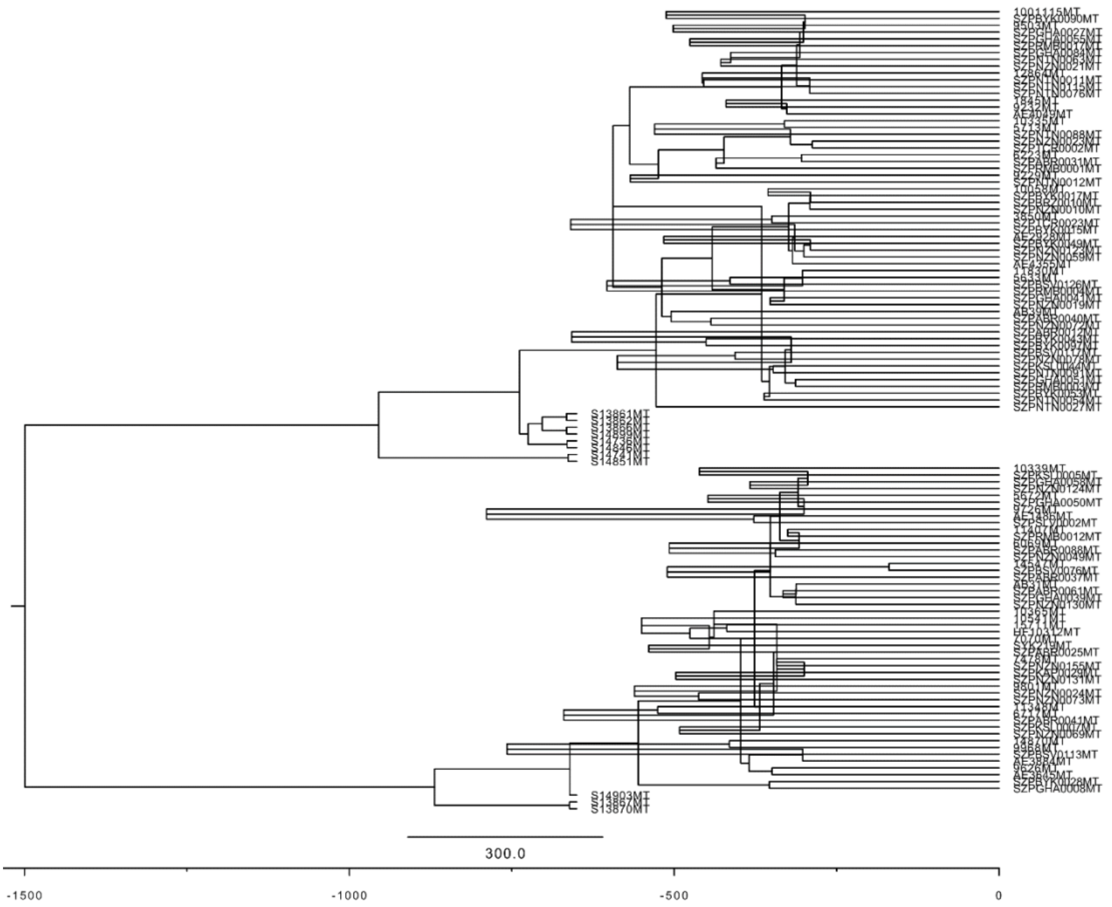

Figure 18. The maximum clade credibility tree of modern and ancient K1a1b1a carriers based on the output of *BEAST*. The tree was visualized using *FigTree*. The x-axis represents the time since the present. The ancient Erfurt genomes were assumed to be sampled 650 years ago.

We show the inferred effective population size trajectory in Figure 19 below. The extremely large uncertainty associated with the inferred population size (see the 95% highest posterior density interval) does not permit definitive conclusions based on this data alone. The median estimate shows population expansion starting about 750 years ago, consistent with autosomal IBD results [51] (Figure 4A and Figure 4E in the main text).

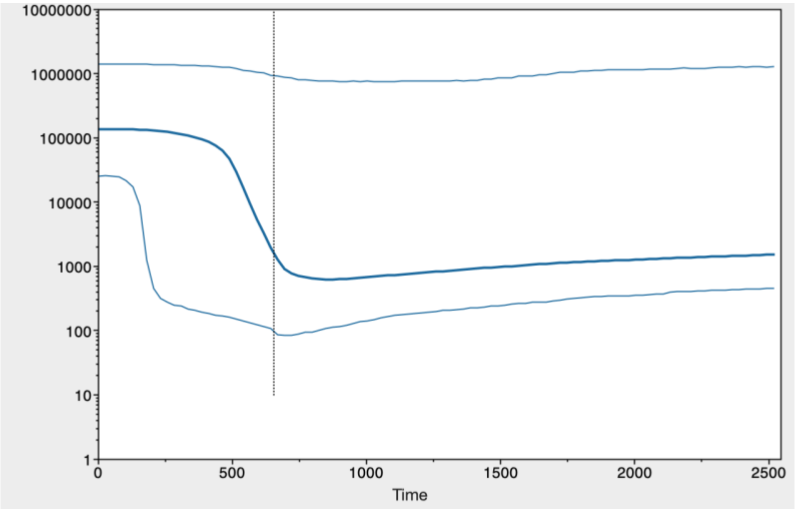

Figure 19. The reconstructed effective population size history based on the mtDNA sequences of modern and ancient K1a1b1a carriers. We ran *BEAST* on 11 ancient EAJ samples and 107 modern samples carrying the K1a1b1a mtDNA haplogroup, as explained in STAR Methods and in Figure 17 above. The plot shows a *Tracer* screenshot of the inferred population size history. The x-axis represents years before present. The y-axis is the effective population size. The thick middle line is the median estimate, and the two thin lines are the upper and lower bounds of the 95% highest posterior density (HPD) interval. Based on these estimates, the AJ population began to expand about 700-800 years ago. The dotted vertical line is the bottom of the 95% HPD interval for the TMRCA of K1a1b1a.

We finally performed the same *BEAST* analysis on the 107 modern carriers alone. Given that all samples are present-day, the mutation rate cannot be learned from the data itself. We used the value of the mutation rate as estimated in the joint modern-ancient analysis ($4.6 \cdot 10^{-8}$ per bp per year) to convert the estimated TMRCA to years ago. All other *BEAST* parameters were as in the joint analysis. The estimated median posterior was 1409 years ago, slightly earlier than in the joint analysis. The 95% highest posterior density (HPD) interval was 478-4041 years ago. Therefore, the availability of ancient samples from 650 years ago pushed the estimated TMRCA backwards ($P<2.2 \cdot 10^{-16}$; two-tailed Wilcoxon test comparing the two posterior distributions). This is expected, given the presence of the polymorphism 16223C/T in the Erfurt carriers, which excludes the possibility that the TMRCA of all modern carriers has post-dated their time.

Section 10. Runs of homozygosity

1. The effect of the coverage on ROH calling

We verified that the number of ROH segments does not depend on the coverage. The correlation between the number of segments and the coverage was r=-0.07 (P=0.8). There was also no correlation between the coverage and the number of ROH segments of length <10cM, which are more prone to error (r=-0.01; P=0.96).

2. ROH levels across Erfurt sub-groups

For each of 16 high-coverage (>400k SNPs) EAJ genomes, we computed the total length of ROH segments longer than 4 cM. We compared the total ROH length between Erfurt sub-groups (Figure 20 below). First, we compared Erfurt-EU and Erfurt-ME genomes and found similar total ROH lengths in the two groups (P=0.43; two-tailed Wilcoxon test). Next, we compared K1a1b1a carriers to the rest of

the samples and found that the total ROH length in K1a1b1a carriers was greater (P=0.03; one-tailed Wilcoxon test).

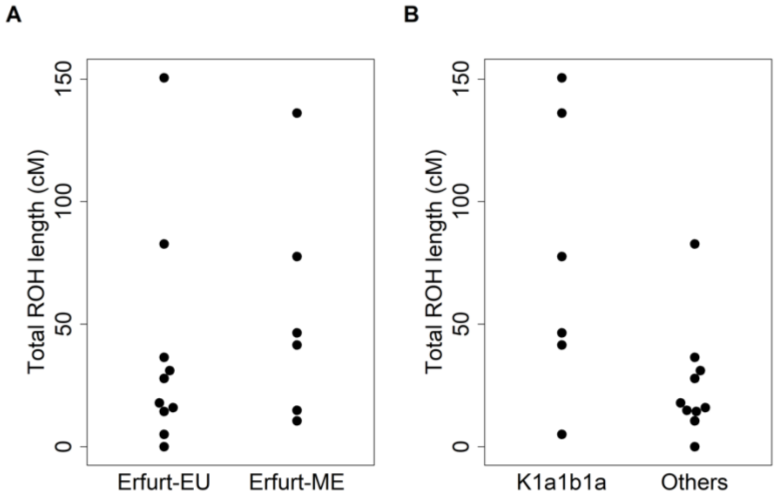

Figure 20. ROH segments across EAJ sub-groups. (A) The total ROH length (cM) per genome in Erfurt-EU and Erfurt-ME individuals. (B) The total ROH length per genome in K1a1b1a carriers compared to all other genomes.

Section 11. Founder alleles

1. Examining biases in the binomial simulations of the founder allele counts

We used binomial simulations to estimate whether the number of founder alleles in Erfurt is expected given that they already experienced the AJ bottleneck (STAR Methods). However, the number of observed founder alleles in EAJ may be underestimated due to a “reference allele bias”. To model the bias in our simulations, we assumed that if the real genotype is heterozygous, there is probability 0.55 that the observed allele will be the reference. [A homozygous genotype (alternate or reference) will be observed correctly.] Hence, the probability to observe the alternate allele changes from p to $p^2 + 0.45 \cdot 2p(1-p)$. When we repeated the simulations with these probabilities, the [2.5,97.5]-percentiles for the number of observed alleles became [12,29].

The expected number of founder alleles in EAJ may also be biased due to the conditioning on exceeding a given frequency in MAJ. This is because alleles that increased in frequency since ancient times to exceed the cutoff in the modern population are included, but alleles that decreased in frequency below the cutoff are not. Therefore, our binomial simulations would tend to overestimate the number of alleles that are expected to be present in EAJ. This problem should exacerbate with higher allele frequency cutoffs. Indeed, when we repeated the analysis with a cutoff of 1% (as opposed to 0.5% above), six alleles were observed in EAJ, which was at the lowest range of the expectation based on binomial simulations ([2.5,97.5]-percentiles: [6,18]). In contrast, when we set the cutoff to 0.1%, 32 alleles were observed in EAJ, compared to simulated [2.5,97.5]-percentiles of [26,49].

In conclusion across analyses, the number of founder alleles observed in EAJ was consistent with the expectation based on modern AJ allele frequencies.

2. Founder alleles in Erfurt sub-groups

Erfurt-EU and Erfurt-ME. The proportion of individuals carrying founder alleles was similar between Erfurt-EU (4/9, 44%) and Erfurt-ME (6/13, 46%). However, this result may be confounded by the higher coverage in Erfurt-EU. We therefore used quasi-Poisson regression to model the number of founder alleles carried by an individual as a function of the group affiliation (Erfurt-EU/Erfurt-ME), with the number of covered founder SNPs as an offset. Mathematically,

$$(1)\quad \log E\left[\frac{\#\,alleles}{\#\,SNPs\,per\,individual}\right] = \beta_0 + \beta_1 \cdot subgroup,$$

where the sub-group was coded as 1 for Erfurt-ME and 0 for Erfurt-EU. Even after adjusting for coverage, the correlation between the number of founder alleles and the group affiliation remained insignificant (Table 3 below).

	Coefficient	Standard error	P-value
Intercept (β_0)	-5.36	0.52	1.62e-09
Erfurt-ME (β_1)	0.50	0.65	0.45

Table 3. The quasi-Poisson regression for the number of founder alleles vs the Erfurt subgroup affiliation. The model is described in Eq. (1).

K1a1b1a carriers. We found that 8/11 (73%) carriers of K1a1b1a also carried at least one founder allele, compared to 3/18 (17%) of carriers of other mtDNA haplogroups (P=0.005, two-tailed Fisher's exact test). Here too, we accounted for differences in coverage using quasi-Poisson regression with an offset,

$$(2)\quad \log E\left[\frac{\#\,alleles}{\#\,SNPs\,per\,individual}\right] = \beta_0 + \beta_1 \cdot haplogroup,$$

where the haplogroup was coded as 1 for K1a1b1a and 0 for all others. Here, the correlation diminished after accounting for coverage, though the P-value remained less than 0.05 (Table 4 below).

	Coefficient	Standard error	P-value
Intercept (β_0)	-5.7424	0.4863	3.58e-12
K1a1b1a (β_1)	1.2631	0.5788	0.038

Table 4. The quasi-Poisson regression for the number of founder alleles vs the mtDNA haplogroup. The model is described in Eq. (2).

Together with the observation of longer ROH among carriers of the K1a1b1a mtDNA lineage (Section 10.2), these results suggest that the K1a1b1a carriers experienced of a narrower bottleneck compared to the rest of EAJ. However, the demographic interpretation of this finding is unclear.

Section 12. Demographic modeling using IBD and ROH segments

1. <u>Inferring the parameters of a single-population model using IBD sharing</u>

Our single-population demographic model is illustrated in Figure 4A of the main text. Under the model, the effective population size has been N_a (diploids) until T_b generations ago, at which point it became N_b for d generations (the bottleneck). The population size then expanded exponentially, until reaching a present-day population size of N_c. We assume generations are discrete. To infer these five parameters based on IBD sharing data, we used the counts of IBD segments across 11 length bins, equally spaced on a logarithmic scale between 4 to 15 cM. We then searched for the parameters of the demographic model that provided the best fit to the data.

To compute the expected number of segments in each length bin, we used theory from Ringbauer et al. (2017) [52] (see also [53-55]). Consider first two present-day chromosomes of length L (Morgan), and fix the coalescence time (i.e., their time to the most recent common ancestor (TMRCA)) to t generations before present. The expected number of IBD segments between these two chromosomes with length in the interval $[\ell_1, \ell_2]$ is [52]

(3) $E\left[n_{seg}(\ell_1,\ell_2) \mid L,t\right] = \int_{\ell_1}^{\ell_2} 4te^{-2t\ell}\left(1+t(L-\ell)\right)d\ell = e^{-2t\ell_1}[2t(L-\ell_1)+1] - e^{-2t\ell_2}[2t(L-\ell_2)+1].$

Denote the historical (diploid) population size as $N(t)$, for $t = 0,1,2.,\ldots$. Under our demographic model (Figure 4A in the main text),

(4) $$N(t) = \begin{cases} N_a, \ t > T_b \\ N_b, \ T_b - d < t \le T_b \\ N_c \cdot \left(\frac{N_b}{N_c}\right)^{\frac{t}{T_b-d+1}}, \ 0 \le t \le T_b - d \end{cases}$$

The probability of the TMRCA at a random locus to equal t is

(5) $P(TMRCA = t) = \frac{1}{2N(t)} \cdot \prod_{\tau=1}^{t-1}\left(1-\frac{1}{2N(\tau)}\right).$

[Eq. (5) is true for any single-population demographic model. It is the probability not to coalesce until and including generation $t-1$, multiplied by the probability of coalescence $(1/2N)$ at generation t.] In the regime $t > T_b$, $N(t) = N_a$ is independent of t, and thus the distribution of the TMRCA is

(6) $P(TMRCA = t) = \frac{1}{2N_a}\left(1-\frac{1}{2N_a}\right)^{t-T_b-1} \prod_{\tau=1}^{T_b}\left(1-\frac{1}{2N(\tau)}\right); \quad t > T_b$

Summing over all t, the mean number of IBD segments of length in $[\ell_1, \ell_2]$ between two chromosomes of length L is

(7) $$\lambda_2(\ell_1,\ell_2\,;L) = \sum_{t=1}^{\infty} E\left[n_{seg}(\ell_1,\ell_2) \mid L,t\right] \cdot P(TMRCA = t) =$$
$$\sum_{t=1}^{T_b} E\left[n_{seg}(\ell_1,\ell_2) \mid L,t\right] \cdot \frac{1}{2N(t)} \cdot \prod_{\tau=1}^{t-1}\left(1-\frac{1}{2N(\tau)}\right) + \frac{1}{2N_a}\prod_{\tau=1}^{T_b}\left(1-\frac{1}{2N(\tau)}\right) \sum_{t=T_b+1}^{\infty} E\left[n_{seg}(\ell_1,\ell_2 \mid L,t)\right]\left(1-\frac{1}{2N_a}\right)^{t-T_b-1}$$

Finally, the mean number of (autosomal) IBD segments of length in $[\ell_1, \ell_2]$ between n diploid genomes is

(8) $\lambda_{IBD,n}(\ell_1,\ell_2) = \left[\binom{2n}{2} - n\right]\sum_{i=1}^{22}\lambda_2(\ell_1,\ell_2\,;L_i),$

where L_i is the length of chromosome $i = 1,\ldots,22$ in Morgan. The pre-factor $\binom{2n}{2} - n$ is the number of haplotype pairs when comparing n diploid individuals to each other. We used *Mathematica* to find a closed-form solution to the term with the infinite sum in Eq. (7). Eq. (8) thus provides the expected number of segments in each length bin under our demographic model.

Following previous studies [52-55], we assumed that the number of segments in each bin inferred from the real data is Poisson distributed with the expected mean (Eq. (8)) and independent across bins. This allowed us to write a composite likelihood for the observed segment counts given a

proposed demographic model. Denote by $\mathcal{B}$ the set of bins and by $c(\ell_1, \ell_2)$ the observed number of IBD segments in the bin $[\ell_1, \ell_2]$ (across $n = 637$ modern genomes). The composite likelihood is

(9) $likelihood = \prod_{(\ell_1,\ell_2)\in\mathcal{B}} e^{-\lambda_{IBD,n}(\ell_1,\ell_2)} \frac{\lambda_{IBD,n}(\ell_1,\ell_2)^{c(\ell_1,\ell_2)}}{c(\ell_1,\ell_2)!}$.

The log-likelihood is (up to an additive constant)

(10) $ll = \sum_{(\ell_1,\ell_2)\in\mathcal{B}} \left[c(\ell_1,\ell_2) \cdot \log\lambda_{IBD,n}(\ell_1,\ell_2) - \lambda_{IBD,n}(\ell_1,\ell_2)\right]$.

We maximized the log-likelihood with respect to the five model parameters (N_a, T_b, N_b, d, N_c) using the function *Deoptim* from the *R* package "Deoptim". When inferring these parameters, we used the following boundaries to the search space: $N_a \in [1000, 50{,}000]$, $N_b \in [100, 5000]$, $T_b \in [20,60]$, $d \in [1,30]$, and $N_c \in [10^5, 10^7]$. These ranges span previous models inferred for the AJ demography [12, 13, 51, 53] and are historically plausible [56]. We ran the optimizer for 5000 steps after setting the seed to 1, and validated that the inferred parameters remained very similar when starting from other seed values. The inferred parameters of the model are listed in Table S5, model (A). We also inferred the model parameters after fixing the bottleneck duration to $d = 1$, as in previous studies [12, 51]. The estimated parameters are listed in Table S5, model (B).

To compute confidence intervals for the inferred model parameters, we used parametric bootstrap. In a naïve implementation of the non-parametric bootstrap, we would resample individuals with replacement. However, detecting IBD sharing between an individual and itself would be nonsensical. We therefore generated each new bootstrap sample as follows. For each segment length bin, we drew a new count for the total number of segments as a Poisson variable with mean equals to the count in the real data. We generated 100 bootstrap samples, and, for each sample, we inferred all model parameters as for the real data. For each parameter θ, we computed the 95% confidence interval for the parameter as $\left[2\hat{\theta} - \theta_{97.5\%}, 2\hat{\theta} - \theta_{2.5\%}\right]$ [57], where $\hat{\theta}$ is the estimate based on the real data, and $\theta_{2.5\%}$ and $\theta_{97.5\%}$ are the 2.5- and 97.5-percentiles, respectively, of the estimates across the bootstrap samples. We calculated the 2.5-percentile as the average of the estimates that ranked second and third (out of 100), and similarly for the 97.5-percentile.

2. Inference using modern ROH segments

We next attempted to infer the parameters of the single-population model (Figure 4A in the main text) using counts of ROH segments in modern genomes. The derivation is exactly as in Eqs. (3) to (10) above, except that in Eq. (8), the pre-factor $\binom{2n}{2} - n$ is replaced by n (the number of haplotype pairs that would generate ROH is n),

(11) $\lambda_{ROH,n}(\ell_1, \ell_2) = n \sum_{i=1}^{22} \lambda_2(\ell_1, \ell_2\,; L_i)$.

We also assumed $N(t) \to \infty$ for $t = 1,2$. This represents the fact that two chromosomes in the same individual cannot coalesce in the immediately following generation, as well as that sib-mating is unlikely. We then found the demographic parameters that maximized the composite likelihood as with the IBD data. Across runs, optimization converged to two distinct optima of similar likelihood, likely due to the small amount of data. The first is listed in Table S5, model (C) ($N_b = 1295$, $T_b = 30$, and $d = 11$). The other optimum was at $N_b = 598$, $T_b = 25$, and $d = 3$. Both models date the end of the bottleneck to around the same time and have similar bottleneck intensities, but they differ in their bottleneck duration.

3. Modeling consanguinity in the ancient individuals

The empirical results (Figure 4D in the main text) suggest that the inferred demographic model (based on IBD sharing in modern genomes; Table S5, model (A)) underestimates the expected number of ROH segments in the ancient genomes. We observed that a few EAJ individuals had very long ROH segments (five individuals with an average of 43.7cM in ROH segments of length >20cM; Figure 4C in the main text), which may result from their parents being related. We thus hypothesized that modeling consanguinity in EAJ may better fit the expectation based on the modern data, and we attempted to fit a model where the demographic parameters are as in Table S5, model (A), but a proportion α of the ancient individuals are offspring of first cousins.

To determine the expected number of ROH segments of a given length under the consanguinity model, we followed Ringbauer et al. (2021) [58]. For children of rth full-cousins, the expected number of ROH segments due to consanguinity in a chromosome of length L Morgan is (see also Eq. (3))

(12) $\lambda_{2,cons}(\ell_1, \ell_2\ ; L) \equiv E\left[n_{seg,cons}(\ell_1, \ell_2 \mid L, r)\right] = \int_{\ell_1}^{\ell_2} \frac{4}{2^m} e^{-\ell m}(2m + (L - \ell)m^2)d\ell,$

where $m = 2r + 4$ is the total number of meioses between the two chromosomes of the child and the most recent common ancestor. For the case of first cousins, where this common ancestor is a great-grandparent, $m = 6$.

The mean number of ROH segments of length in $[\ell_1, \ell_2]$ due to consanguinity in n genomes of children of first cousins is

(13) $\lambda_{ROH,cons,n}(\ell_1, \ell_2) = n \sum_{i=1}^{22} \lambda_{2,cons}(\ell_1, \ell_2\ ; L_i).$

The mean number of ROH segments between two ancient chromosomes of length L due to genetic drift, i.e., due to coalescence under the demographic model, is

(14) $\lambda_{2,anc}(\ell_1, \ell_2\ ; L) = \sum_{t=T_E+3}^{\infty} E\left[n_{seg}(\ell_1, \ell_2) \mid L, t - T_E\right] \cdot P(TMRCA = t) = \sum_{t=T_E+3}^{T_b} E\left[n_{seg}(\ell_1, \ell_2) \mid L, t - T_E\right] \cdot \frac{1}{2N(t)} \cdot \prod_{\tau=T_E+3}^{t-1}\left(1 - \frac{1}{2N(\tau)}\right) + \frac{1}{2N_a} \prod_{\tau=T_E+3}^{T_b}\left(1 - \frac{1}{2N(\tau)}\right) \sum_{t=T_b+1}^{\infty} E\left[n_{seg}(\ell_1, \ell_2 \mid L, t - T_E)\right]\left(1 - \frac{1}{2N_a}\right)^{t-T_b-1}.$

T_E is the number of generations ago when the Erfurt population has lived. We assumed a generation interval of 25 years, slightly lower than previous studies [59-63], given that early AJ often married extremely young [64]. Given our radiocarbon dating to the 14th century, i.e., about 650 years ago, this gives $T_E = 26$. $N(t)$ is given by Eq. (4). Eq. (14) is the same as Eq. (7), except that no coalescence is possible until T_E generations ago and that the number of generations to the TMRCA is $t - T_E$. We started the sums at $T_E + 3$ to represent the constraint of no sib-mating. The mean number of ROH segments in n ancient genomes due to drift is

(15) $\lambda_{ROH,anc,n}(\ell_1, \ell_2) = n \sum_{i=1}^{22} \lambda_{2,anc}(\ell_1, \ell_2\ ; L_i).$

Finally, the total number of ROH segments of length in the interval $[\ell_1, \ell_2]$ in the ancient genomes has mean

(16) $(1 - \alpha)\lambda_{ROH,anc,n}(\ell_1, \ell_2) + \alpha\left[\left(1 - \frac{4}{2^m}\right)\lambda_{ROH,anc,n}(\ell_1, \ell_2) + \lambda_{ROH,cons,n}(\ell_1, \ell_2)\right],$

where $\lambda_{ROH,anc,n}$ is the expected number of ROH segments due to genetic drift (Eq. (15)), and $\lambda_{ROH,cons,n}$ is the expected number of segments due to consanguinity (Eq. (13)). The term $\left(1 - \frac{4}{2^m}\right)\lambda_{ROH,anc,n}(\ell_1, \ell_2)$ represents ROH in children of first cousins in genomic regions where the two chromosomes do not coalesce at the shared great-grandparents. We then assumed, as above,

that the observed total number of (ancient) ROH segments (across the $n = 16$ ancient genomes) in each length bin follows a Poisson distribution with the given mean. This gave a composite-likelihood similar to Eq. (9).

We then fixed all demographic parameters to their inferred values as in Table S5, model (A) and used the optimization procedure to find the value of α that maximized the log-likelihood. Note that we used neither modern IBD nor modern ROH data. For the set of bins $\mathcal{B}$, we used (here and in all other models based on ancient ROH) 29 bins equally separated on a logarithmic scale between 4 to 40 cM. This is different from modern data in that we also considered relatively long ROH segments. The long segments likely appeared because (*i*) the parents of some individuals may have been related, and (*ii*) the individuals lived closer in time to the bottleneck.

The inferred proportion of individuals who were children of first cousins (Table S5, model (D)) was $\alpha = 0.22$, which corresponds to 3-4 individuals out of the total of 16. This estimate is reasonable given the distribution of total ROH lengths (Figure 4C in the main text). However, the fit to the ROH counts did not sufficiently improve (Figure 4D in the main text), possibly as consanguinity generates predominantly very long segments (mean nearly 17cM for children of first cousins), whereas the ROH counts were underestimated at shorter lengths. We therefore no longer considered consanguinity in our next models.

4. <u>Modeling a narrower or a longer bottleneck</u>

We next hypothesized that the excess of ROH segments in EAJ is due to the EAJ population experiencing a narrower or a longer bottleneck compared to what we inferred based on IBD sharing in MAJ (Table S5, model (A)). In the following, we fixed some of the parameters of the modern-based model (Table S5, model (A)) and inferred the other parameters using ancient ROH data to fit models with a narrower or a longer bottleneck.

For a model with a narrower bottleneck, we fixed the bottleneck starting time to $T_b = 41$ and inferred the ancestral population size (N_a) and the bottleneck size (N_b) based on the counts of ROH segments in the ancient genomes. We assumed that the population size remained at N_b until the time of the EAJ individuals. In other words, the EAJ population size history has been

(17) $N(t) = \begin{cases} N_a, \ t > T_b = 41 \\ N_b, \ T_E = 26 < t \leq T_b \end{cases}.$

We plugged this expression for $N(t)$ into Eq. (14) and used Eq. (15) to compute $\lambda_{ROH,anc,n}(\ell_1, \ell_2)$, the expected number of ROH segments in the ancient genomes under our demographic model (without consanguinity). We again assumed a Poisson distribution for the number of segments in each bin, and used the optimization procedure to find the values of N_a and N_b that maximized the composite-likelihood. [While our focus was on the bottleneck size N_b, and we generally did not attempt the interpret the (highly uncertain) estimate of N_a, we found numerically that allowing N_a to vary improved the fit.]

This above described procedure did not yet use any modern data. Accordingly, we did not infer the values of d and N_c, as these do not appear in Eq. (17) and thus do not affect ancient ROH levels. Once we estimated N_a and N_b using the ancient ROH data, we fixed these values (along with T_b, which is fixed to its value from Table S5, model (A)) and estimated d and N_c using modern IBD data, in the same way we inferred the full model (assuming $T_b - d \leq T_E$). The complete set of inferred model parameters is given in Table S5, model (E). The fit of the model to the ancient ROH data is shown in Figure 4D in the main text.

We used a similar procedure to infer the parameters of a model with a longer bottleneck. First, we fixed N_a and N_b to their values from Table S5, model (A), giving the following EAJ population size history,

(18) $$N(t) = \begin{cases} N_a = 47{,}961, \ t > T_b \\ N_b = 1{,}563, \ T_E = 26 < t \leq T_b \end{cases}$$

We then used the ancient ROH data to infer the value of T_b (assuming $80 \geq T_b \geq 41$) by comparing the inferred segment count based on the data to the expectation based on Eq. (15), as above. We finally used the modern IBD data to infer the parameters d and N_c. The inferred parameters are listed in Table S5, model (F), and the fit is shown in Figure 4D in the main text. Both a narrower and a longer bottleneck fit the ancient ROH data (in particular the narrower model). However, neither model fit the modern IBD data (Figure S5A).

5. Joint inference based on modern and ancient data

To identify model parameters that would fit both modern and ancient data, we used the same five-parameter model (Figure 4A in the main text) with population size history $N(t)$ given in Eq. (4), and attempted to infer its parameters using ancient and modern data jointly. Recall that the log-likelihood for the modern IBD data was

(19) $$ll_{modern} = \sum_{(\ell_1,\ell_2)\in\mathcal{B}} c_m(\ell_1,\ell_2) \cdot \log \lambda_{IBD,n_m}(\ell_1,\ell_2) - \lambda_{IBD,n_m}(\ell_1,\ell_2),$$

where $c_m(\ell_1,\ell_2)$ is the number of IBD segments of length in $[\ell_1,\ell_2]$ shared between any pair of haplotypes among $n_m = 637$ modern genomes, and $\lambda_{IBD,n_m}(\ell_1,\ell_2)$ is the expectation based on Eq. (8). Similarly, for the ancient data,

(20) $$ll_{ancient} = \sum_{(\ell_1,\ell_2)\in\mathcal{B}} c_a(\ell_1,\ell_2) \cdot \log \lambda_{ROH,anc,n_a}(\ell_1,\ell_2) - \lambda_{ROH,anc,n_a}(\ell_1,\ell_2),$$

where $c_a(\ell_1,\ell_2)$ is the number of ROH segments of length in $[\ell_1,\ell_2]$ in any of the $n_a = 16$ ancient genomes, and $\lambda_{ROH,anc,n}(\ell_1,\ell_2)$ is the expectation based on Eq. (15). We defined a joint log-likelihood as

(21) $$joint\ ll = \frac{ll_{modern}}{\binom{2n_m}{2} - n_m} + \frac{ll_{ancient}}{n_a}.$$

This definition addresses the issue that the number of haplotype pairs is about 50k times larger in the modern IBD data compared to the ancient ROH data. Under Eq. (21), each log-likelihood class (modern IBD/ancient ROH) contributes roughly equally to the log-likelihood. For both IBD and ROH, $\mathcal{B}$ was 29 bins between [4,40]cM. We then searched for the maximum likelihood parameters as before, except that we enforced the time of EAJ sampling ($T_E = 26$) to be within the bottleneck (i.e., $T_b \geq T_E \geq T_b - d$). The inferred parameters are listed in Table S5, model (G). The fit to the modern data was still imperfect (Figure S5B).

6. Inferring the parameters of a two-population model

To reconcile the demographic models of EAJ and MAJ, we expanded the model to account for substructure in AJ during the Middle Ages. While an expanded model can take various forms, we sought to minimize overfitting, and hence added only a single parameter. In our model, the AJ population split T_b generations ago into two groups. The first represents EAJ, with effective population size N_b. The second had population size $N_a - N_b$. This is an arbitrary choice, in order to model different population sizes for the two groups without increasing the number of parameters. The populations then merged d generations later, with proportions f and $1 - f$, respectively, and then expanded exponentially until reaching the present population size. Note that we did not explicitly

model the substructure within EAJ, again in order not to add parameters, and given that we have modelled substructure in AJ as a whole. The model is illustrated in Figure 4E in the main text.

We defined a joint modern-ancient likelihood as in Eqs. (19)-(21). The likelihood based on the ancient ROH data remains the same, as the model is identical to that of Figure 4A (in the main text) from the perspective of the EAJ population. As above, we assumed that the bottleneck must have spanned the time of EAJ, i.e., $T_b > T_E > T_b - d$. For the likelihood based on the modern IBD data, we modified Eq. (7) (for the mean number of segments between a pair of chromosomes of length L) as follows,

$$(22)\lambda_2(\ell_1, \ell_2\,; L) = \sum_{t=1}^{T_b-d} E\left[n_{seg}(\ell_1, \ell_2) \mid L, t\right] \cdot \frac{1}{2N(t)} \cdot \prod_{\tau=1}^{t-1}\left(1 - \frac{1}{2N(\tau)}\right) + \prod_{\tau=1}^{T_b-d}\left(1 - \frac{1}{2N(\tau)}\right) \cdot \sum_{t=T_b-d+1}^{T_b} E\left[n_{seg}(\ell_1, \ell_2) \mid L, t\right] \cdot \left[\left(1 - \frac{1}{2N_b}\right)^{t-(T_b-d+1)} \cdot \frac{1}{2N_b} \cdot f^2 + \left(1 - \frac{1}{2(N_a - N_b)}\right)^{t-(T_b-d+1)} \cdot \frac{1}{2(N_a - N_b)} \cdot (1-f)^2\right] + \prod_{\tau=1}^{T_b-d}\left(1 - \frac{1}{2N(\tau)}\right) \cdot \left[\left(1 - \frac{1}{2N_b}\right)^d f^2 + \left(1 - \frac{1}{2(N_a - N_b)}\right)^d (1-f)^2 + 2f(1-f)\right] \cdot \frac{1}{2N_a} \cdot \sum_{t=T_b+1}^{\infty} E\left[n_{seg}(\ell_1, \ell_2) \mid L, t\right] \cdot \left(1 - \frac{1}{2N_a}\right)^{t-T_b-1}.$$

The population size history is

$$(23)N(t) = \begin{cases} N_a,\ t > T_b \\ N_b \text{ or } N_a - N_b,\ T_b - d < t \le T_b \\ N_c \cdot \left(\frac{N_b}{N_c}\right)^{\frac{t}{T_b-d}},\ 0 \le t \le T_b - d \end{cases}.$$

In Eq. (22), for coalescence to occur within the first sub-population, both lineages must descend from that population, which happens with probability f^2, and similarly for the second sub-population (probability $(1-f)^2$). For coalescence to happen in the ancestral (pre-split) population, coalescence must not have happened during the bottleneck. This is the case if both lineages descended from the first population (probability f^2) and then there was no coalescence (probability $\left(1 - \frac{1}{2N_b}\right)^d$), if both lineages descended from the second population followed by no coalescence ($(1-f)^2\left(1 - \frac{1}{2(N_a-N_b)}\right)^d$) or if each lineage descended from a different population (probability $2f(1-f)$). We used the same optimization procedure as in the other cases to obtain the maximum likelihood estimate for the six parameters (N_a, N_b, T_b, d, f, and N_c). To compute confidence intervals, we used parametric bootstrap as for the single population model. Here, we re-sampled segment counts per bin for both modern IBD and ancient ROH from Poisson variables with means as in the real data.

Section 13. Testing demographic models using simulations

1. Model selection

The improved fit of the IBD and ROH data to the two-population model could be due to its increased complexity. To evaluate whether the fit is sufficiently improved to justify the additional parameter, we used parametric bootstrap [65], testing the null hypothesis that the real data comes from the single-population model. We simulated segment length counts under the (five parameter) single-population model. For each simulated dataset, we fit both the single-population and the (six-parameter) two-population model, and we recorded the increase in composite log-likelihood when (over)fitting the more complex model. We then determined whether the increase in likelihood observed in the real data is beyond what is expected when the data is truly derived from the single-population model.

To simulate from the single-population model, we used the best-fit parameters we inferred jointly from the MAJ and EAJ data (Table S5, model (G)). We calculated the expected number of segments in each length bin (29 bins from 4 to 40 cM) for both IBD segments in MAJ (Eq. (8)) and ROH segments in EAJ (Eq. (15)). We then drew a new count at each length bin as a Poisson variable with mean equals to the expected count. For each simulated dataset, we maximized the log- composite-likelihood based on Eq. (21) for either the single-population or the two-population model. Over 100 simulated datasets, the difference in the optimal log-likelihood between the two models, $ll_{two\ populations} - ll_{single\ population}$, was in the range [-0.003, 0.09]. In the real data, the log-likelihood difference (based on the models in Table S5, models (G) and (H)) was 0.21. We thus conclude that, with P<0.01, we can reject the hypothesis that the real data derives from the single-population model.

2. <u>Simulations of the two-population model</u>

We sought to validate, using simulations, that we can use data of the type available to us to accurately infer the two-population model parameters. We used *ARGON* version 1.0 [66] to simulate the demographic model shown in Figure 21 below (all population sizes are haploids).

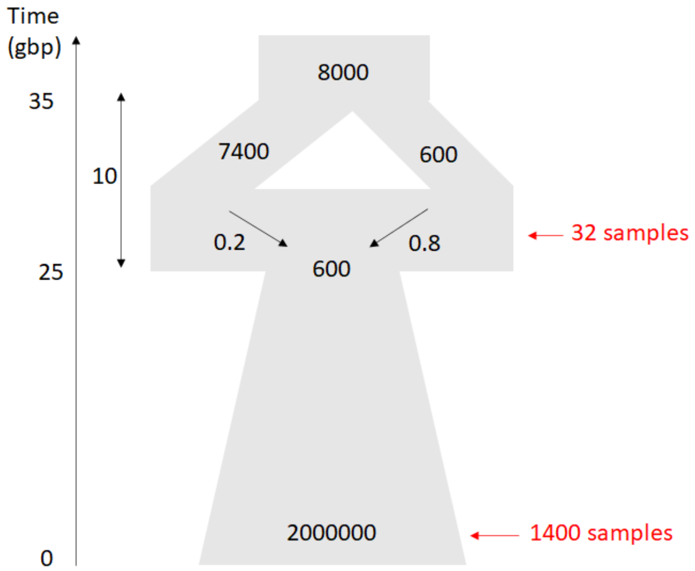

Figure 21. An illustration of the simulated demographic model. All times are in generations before present (gbp). The width of the diagram at different time points increases (schematically) with the effective population sizes. The indicated population sizes are in haploid individuals. In our simulations, we sampled either 1400 haploid chromosomes at present, or 32 haploid chromosomes 26 generations before present (red arrows), representing our modern and ancient samples, respectively.

To mimic the extreme imbalance in the real data between the number of modern and ancient observations, we sampled 1400 haploid individuals from the present-day population ("modern" data), and then ran the simulation again and sampled 32 haploid individuals from the right population at the end of the bottleneck ("ancient" data). These sample sizes roughly correspond to our 637 modern genomes and 16 ancient genomes. For each individual we simulated a single chromosome of length 280 Mb with the default recombination rate of 1cM/Mb. The simulator provided ground-truth information on all IBD segments shared between all pairs of either "modern" or "ancient" individuals. [We computed pairwise IBD also for the "ancient" genomes (and not runs of homozygosity), in order

to generate sufficient amount of data, given that we only simulated a single chromosome.] We then recorded the number of segments per length bin (29 bins between [4-40] cM).

We first used the simulated modern data alone to infer the demographic parameters of the single-population model (Figure 4A in the main text). We used the same methods as for the real data. The inferred parameters were $N_a = 46.1 \cdot 10^3$, $N_b = 780$, $T_b = 35$, $d = 12$, and $N_c = 1.8 \cdot 10^6$ (population sizes are in haploid individuals). As we observed for the real data (Figures 4B and 4D in the main text), the fit was good for the modern IBD data, but it underestimated the number of ancient ROH segments (Figure 22A below). We then used the simulated modern and ancient data jointly to infer all six parameters of the two-population model (Figure 4E in the main text). The inferred parameters were very close to their simulated values: $N_a = 9.1 \cdot 10^3$, $N_b = 704$, $T_b = 36$, $d = 13$, $f = 0.84$ and $N_c = 3.4 \cdot 10^6$. The fit was now good for both modern and ancient data (Figure 22B below). These results, while not comprehensive, hint that even given the relative scarcity of the ancient ROH data, our method should be able to accurately infer the parameters of the two-population model.

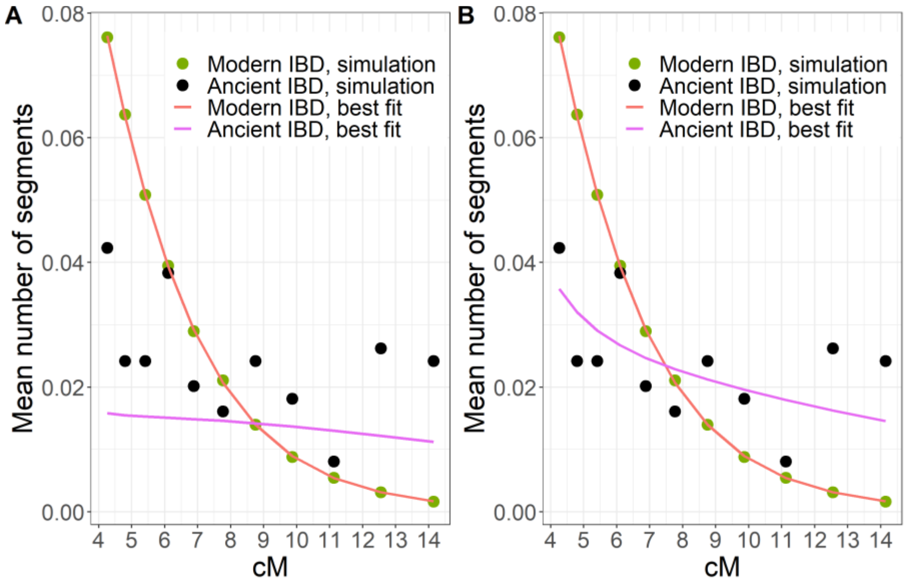

Figure 22. Simulated and fitted IBD segment counts. (A) The mean number of IBD segments per haplotype pair across segment length bins. We simulated those IBD segments based on the demographic model shown in Figure 21 above. Symbols show simulated mean counts (legend). Lines (legend) show the best fit based on a single-population demographic model (Figure 4A in the main text). (B) The same simulated data as in (A), but with the fitted lines based on the two-population demographic model (Figure 4E in the main text).

Section 14. Imputation accuracy and the pathogenic variants

1. Mendelian inconsistency

To test the accuracy of imputation using *PHCP*, we used the two Erfurt families to estimate the rate of Mendelian inconsistency. The analysis included SNPs both genotyped and fully imputed, because even genotyped SNPs were imputed from haploid to diploid. Given that only one parent was available from each family, Mendelian inconsistency would be observable only when the parent and child carry opposing homozygous genotypes. Thus, in each family, we started with all SNPs imputed as homozygous in the parent, and counted the number of SNPs that were imputed in each child as

homozygous to the opposite allele. Overall, we tested three parent-child pairs: mother (I14850) and son (I14853) and mother and daughter (I14898) from family A, and father (I14904) and daughter (I13869) from family B (Table 5 below). To compare the results to a baseline, we repeated the analysis with the mother from family A and the daughter from family B, and for the mother with an arbitrarily selected high-coverage genome (I13866) from Erfurt-EU (since the two families belong to Erfurt-EU).

The proportion of inconsistent SNPs was 0.19-0.23% for the two children who were covered in >500k SNPs, and 0.59% for the child with 113k SNPs (Table 5 below). In comparison, the inconsistency rate was 2.13-2.15% in unrelated individuals (Table 5 below).

	**Number of covered SNPs**	**Number of imputed homozygous SNPs**	**Number of covered SNPs**	**Proportion of inconsistent SNPs (%)**	**Number of covered SNPs**	**Proportion of inconsistent SNPs (%)**
	**Parent**	**Parent**	**Child 1**	**Child 1**	**Child 2**	**Child 2**
**Family A**	$562 \cdot 10^3$	$15.52 \cdot 10^6$	$519 \cdot 10^3$	0.23%	$113 \cdot 10^3$	0.59%
**Family B**	$701 \cdot 10^3$	$15.52 \cdot 10^6$	$601 \cdot 10^3$	0.19%		
**Mother of family A vs unrelated**	$562 \cdot 10^3$	$15.52 \cdot 10^6$	$601 \cdot 10^3$	2.13%	$643 \cdot 10^3$	2.15%

Table 5. Evaluating the imputation accuracy of PHCP using Mendelian inconsistency. We tested the rates of Mendelian inconsistency in the imputed genomes of families A and B and compared to the inconsistency in unrelated individuals. The table presents the number of covered SNPs (before imputation) in all individuals, the number of homozygous SNPs in the imputed genome of the parent (this includes genotyped SNPs, as these were also imputed from haploid to diploid), and the percentage of SNPs that are homozygous to the opposite allele in the child (out of the number of homozygous SNPs in the parent). In family A, Child 1 is the son (I14853) and Child 2 is the daughter (I14898). The unrelated individuals are the mother from family A (I14850) and the daughter from family B (I13869; "Child 1"), or another unrelated individual (I13866; "Child 2").

2. Concordance against masked founder alleles

We masked genotypes in the 216 founder SNPs defined above and in three pathogenic variants that were genotyped and were present in at least one EAJ genome (F11/p.E135X, F11/p.F301L, and LRRK2/p.G2019S; Data S2, Table 6). We then imputed these SNPs and tested the concordance between genotyped and imputed alleles. Among the 219 SNPs that were tested, 9 were not present at the reference panel and were not imputed.

There were overall 20 cases (across all individuals and SNPs) where the ancient (pseudo-haploid) genotype showed the alternate allele. Among these, we correctly imputed at least one alternate allele in 15 cases. In the remaining five cases, the imputed genotype was homozygous reference, and we thus estimate the false negative rate as 5/20=25%. This is an upper bound, as some of these errors may be false positives in the ancient DNA genotypes. Interestingly, the false negative rate was 15% in

Erfurt-ME (2/13) but 50% in Erfurt-EU (3/6), though the number of variants is too small to draw any conclusion (P=0.26; Fisher's exact test).

We evaluated the false-positive rate as follows. First, we identified all cases, across the 29 individuals that were tested for founder SNPs (i.e., without the children of family A and B and without the individual that was not covered in any of the founder SNPs), and across all masked SNPs, where the pseudo-haploid genotype was the reference allele. We then computed the proportion of these cases where an alternate allele was imputed. (In all of these cases, the imputed genotype was heterozygous.) The observed proportion was 13/2541=0.005. This is an upper bound for the false positive rate, because in some cases, the true genotype may have been heterozygous, but only the reference allele was observed. To quantify this, we computed the expected number of cases where the true genotype should have been heterozygous, based on MAJ allele frequency (*gnomAD*), but the observed allele is the reference. Specifically, we multiplied the MAJ frequency of each founder allele by the number of genomes that had the reference allele at this SNP and summed over all SNPs. The expected number of heterozygotes was 24.04, greater than the imputed number of alternate alleles (13). This could be due to (i) false negatives of imputation; or (ii) lower frequency of the founder alleles in EAJ compared to MAJ (Figure S5C). Either way, our estimate of the imputation false positive rate (13/2541, or about 1/195) is likely as an upper bound.

3. Concordance between *PHCP* and *GLIMPSE*

To evaluate the concordance between *PHCP* and *GLIMPSE*, we compared their output on the pathogenic variants (STAR Methods; Data S2, Table 6). We first considered eight variants that were not genotyped and that were imputed by *PHCP* as having at least one alternate allele with posterior probability >97%. A carrier of one variant was not run in *GLIMPSE* due to low coverage. For the remaining variants, *GLIMPSE* imputed at least one alternate allele with probability >50% in 6/7 variants. We then considered six variants where *GLIMPSE* imputed an alternate allele with probability >97%. *PHCP* imputed the alternate allele with probability >50% for all such variants.

4. Characterizing the detected pathogenic variants

Some of the AJ-enriched pathogenic variants (Table 1 of the main text) were previously dated using genomic modern data. Among dominant variants, the *BRCA1* c.68_69delAG (also known as 185delAG) is one of three common variants in *BRCA*1/2 genes in AJ [67] and is known to increase the lifetime risk of breast and ovarian cancer to 84% and 35%, respectively [68]. It is also present in Iraqi and other Jews, and was previously dated to descend from a single founder who lived 47-77 generations ago [69]. The G2019S variant on *LRRK2* (which was genotyped) increases the risk of Parkinson's disease [70]. The variant is common in North-Africans and it was found in about 20% and 40% of Parkinson's disease cases in AJ and North-Africans, respectively. The variant was previously dated to a few thousands of years ago [71, 72]. The remaining variants were recessive. The c.84dupG variant in *GBA* is an AJ-specific variant for Gaucher disease (along with the more common N409S variant). It was previously dated to 56 generations ago [73]. Two variants on *F11*, leading to Factor XI deficiency, were genotyped. The type II variant (E135X; also known as E117X) is present in other Jewish and Arab populations [74] and was also found in a Levant individual living 9kya [75]. It was previously dated to 120-189 generations ago [76]. The Type III variant (F301L; also F283L) is AJ-specific and was dated to at least 31 generations ago [76]. The familial Mediterranean fever *MEFV* variant V726A is found in multiple Middle Eastern populations and was dated to a few thousand years ago [77-79].

Other recessive pathogenic variants we detected include the cystic fibrosis *CFTR* variant G542X [80], the retinitis pigmentosa *DHDDS* variant 124A>G [81], the Usher syndrome (type 3) *CLRN1* variant N48K [82], and the glycogen storage disease (type 1A) *G6PC* variant R83C [83]. We also identified a female child carrier of the *ACADS* c.319C>T variant, who had a 44% probability to be homozygous (Data S2, Table 6) and thereby affected by acyl CoA dehydrogenase deficiency. While the disease may be associated with failure to thrive [84], we did not notice any pathologies in her skeleton that might be associated with this phenotype.

Section 15. Phenotypes

1. Lactase persistence

The lactase persistence dominant allele rs4988235/T [85] is known to have a much lower frequency in MAJ compared to Europeans (10.0% vs 60.1%, respectively; *gnomAD*; Data S2, Table 6). This difference may reflect a gradient within Europe, where the allele frequency is 69.8% in North-Western Europeans but only 33.0% in Southern European (*gnomAD*). To estimate the frequency of the allele in EAJ, we excluded the children from both families, leaving 30 individuals (60 chromosomes). While the SNP was captured, it was missing in some individuals, and we used the most likely genotype based on the *PHCP* imputation. The T allele frequency in EAJ was 11.7% (7/60; 95% CI: [6,22]%), similar to the MAJ allele frequency. The 95% confidence interval was computed using Wilson's method, as implemented in the *binconf* function from the *Hmisc* package in *R*.

2. Pigmentation

We used the same method as above. The blue eye recessive allele rs12913832/G [86] had frequency 55% in EAJ (33/60; 95% CI: [42,67]%; Data S2, Table 6), again similar to the MAJ frequency (54.8%). The red hair recessive alleles rs1805007/T, rs1805008/T, and rs1805009/C [87] were present in 8.3% of EAJ (5/60; 95% CI: [3.6,18.1]%, Data S2, Table 6) compared to 12.4% in MAJ. No homozygous carriers were observed.

3. Plague-related alleles

A recent study [88] found a sharp change in allele frequency for rs17514136 and rs10839708 between 16th-century plague victims in Ellwangen, Germany, and modern individuals from the same town. We estimated the allele frequency in EAJ as above. We found that allele frequencies were similar between 14th-century EAJ and MAJ (P=0.18 and 0.81, respectively; one-tailed binomial test in the direction of the change observed in Immel et al [88]; Data S2, Table 6).

4. Stature estimation

We reconstructed body height for two individuals using the anatomical method [89], and compared the results with those obtained using the mathematical method [90] based on several long bone measurements. As the results matched well, we used Pearson's regression formulae to reconstruct body height in all 14 adult individuals whose long bones were sufficiently preserved (Data S2, Table 7). We then used, for each individual, the mean over the estimates from all available bones.

5. A polygenic score for height

We aimed to test the ability of polygenic scores to predict stature in EAJ. We used summary statistics from [91] without additional adjustments. Our data included 704,830 SNPs overlapping the summary statistics. We calculated the score for each individual using *Plink* version 1.9 [92] (--score) with the "sum" option and otherwise default settings, such that missing genotypes were imputed to their allele

frequencies. The mean number of informative SNPs per individual (among those with osteological height estimates) was 340,799 (range 150,420-507,616).

We compared the osteological height estimates to the polygenic scores in 13 unrelated adults (Data S2, Table 7; the daughter from Family B was excluded). We added 9.84 cm, the mean difference between males and females in our sample, to the height of each female. The osteological heights and the polygenic scores were correlated ($r = 0.48$, 95% CI: [-0.10,0.81]; Figure 23 below). While our sample size is too small to reach a definitive conclusion, the ability to genetically predict the stature of ancient individuals, even if at reduced accuracy, agrees with recent studies [93, 94].

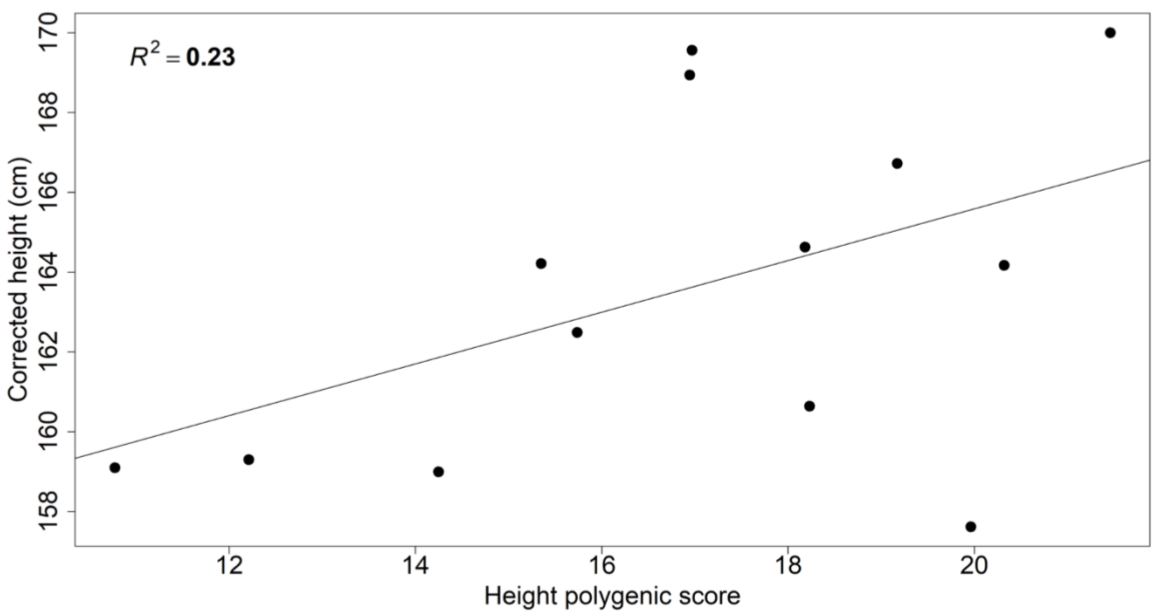

Figure 23. The correlation between the estimated stature and the polygenic score for height. For each individual, we plot the estimated height (mean over all available estimates, shifted up by 9.84 cm for females (the empirical mean difference between the sexes); Data S2, Table 7) and the polygenic score for height based on summary statistics from [91]. We also plot the linear regression line. The proportion of variance in height explained by the score was 23% ($r = 0.48$, 95% CI: [-0.10,0.81]).

Section 16. Historical interpretation of the genetic results

1. <u>Models for AJ ancestral sources</u>

The good fit of *qpAdm* models for EAJ that had Italy as a source (particularly Southern Italy) provides some support for (although do not definitively prove) the theory of AJ origins in Italy (Section 1.1). Southern Italy is one of the very few places in Europe where there is evidence for Jewish demographic and cultural continuity from the late Roman into the early Medieval period and beyond [5, 95-102]. During this timeframe, the Jewish communities of Southern Italy were at the crossroads of Jewish Mediterranean life. They were in direct contact with the Jewish communities of Byzantine and early Muslim Palestine from whom they received liturgical traditions that they transmitted into Europe and that later turned up in the AJ prayer book. They were also in touch with Jewish communities elsewhere in the Eastern Mediterranean by virtue of the fact that Southern Italy was part of the Byzantine Empire into the late 11th century.

All the evidence currently available indicates that during the Roman and early Medieval periods Jews were highly integrated in Southern Italy. There is historical evidence that there was at least some gene flow between Jews and non-Jews in Southern Italy, because, in the late Roman and early Medieval periods, imperial and ecclesiastical authorities tried to prevent the practice of intermarriage between Jews and Christians, as well as the phenomenon of conversion of non-Jews to Judaism. When, in due course, highly accomplished and connected Jews from Southern Italy started moving north, they were joined by others from Central and Northern Italy. For example, the Kalonymus family—a Jewish family from the Italian peninsula—is known to have had major impact on AJ intellectual life in 10th-century Mainz and Speyer [5, 103]. This was the multilayered migratory legacy that may be reflected in the Southern European-related genetic ancestry we observed in our models for the genomes of Erfurt Jews.

We also found possible fit for a model with Greek as a Mediterranean European source. During the Hellenistic age, from the 4th century BCE onwards, Jews moved north into Asia Minor and Greece, including islands such as Delos, which was a major commercial hub at the times. While relations between Jews and Greeks were strained at first, there was much interaction going on during the Roman period. There is plenty of archaeological and inscriptional evidence for vibrant Jewish communities in both Greece and on the West coast of Asia Minor in cities that had originated as Greek colonies [104]. Later on, from the early Roman Imperial period onwards, we also find Jewish communities in Southern Italy and in Sicily in cities that had begun their life as Greek colonies, particularly in Taranto (Puglia) and Siracusa (Sicily) [105].

Our estimate of about 15% Eastern European-related ancestry is consistent with a previous study [40]. The identification of this source as Eastern European relies on the f_4 results (Figure S3) and the *qpAdm* models (Table S3). However, this ancestry might derive from a broad area across Central or Eastern Europe, particularly given the recorded migration into Erfurt from Bohemia, Moravia, and Silesia (Section 2.2). The genetic data suggested a high degree of endogamy in AJ through the last ≈700 years. Historical evidence indicates that the social practice of intermarriage between Jews and Christians was frowned upon by medieval Jewish and Christian authorities [106, 107]. Our genetic results suggest that in practice there was indeed very little gene flow into the Jewish community since this period. This suggests that the majority of Eastern European-related gene flow has predated the 14th century.

2. Limitations of the model

Models with a South-Italian source were more frequently favored by *qpAdm* (Table S3) and have a plausible historical basis (above). However, these models suggested that only about 20% of EAJ ancestry derived from Middle Eastern sources. This is less than previous estimates based on modern SNP and sequencing data [40, 51]. This may also be interpreted to imply that present-day AJ derive only a small proportion of their ancestry from ancient Judaeans; and if so, most AJ ancestry would owe its origin to European converts. While this is one possible explanation, modern Italians themselves have had much higher proportions of ME admixture since at least European Imperial Roman times [43] and this is especially the case in modern Southern Italy [108]. Thus, an alternative explanation for these observations is that the true ME proportion in AJ is higher than in our fitting model, and that the actual contribution of Italians is not as large as suggested by this analysis. Under this scenario, good *qpAdm* fits are obtained using South-Italians as sources simply because they are a modern population that harbors a relatively high proportion of ME ancestry. If this alternative explanation is right, the true ME proportion could be higher than in our models, e.g., close to the 30-50% estimates from previous studies [40, 51] or when modeling EAJ using North-Italians.

In an opposite scenario, AJ may have no ancestry at all from the ancient Levant. This could be the case if an unsampled Italian population (with more Levantine-like admixture than in modern South-Italians) is the source of all the Levantine-like ancestry seen in AJ. At present, we believe all types of scenarios are plausible. Co-analysis of ancient DNA data from the Middle East and the Italian peninsula from Antiquity and the early Medieval period would make it possible to distinguish them.

Further complicating the interpretation of the Middle Eastern origins are multiple demographic changes that have also affected the Middle East during the past two millennia. Most notably is African admixture, as documented in multiple populations [109-111], particularly Saudis [112, 113] and Egyptians [114]. Bottlenecks and population structure were identified in Druze, Bedouin, and Lebanese [111, 113, 115, 116], and temporal changes in ancestry were observed in Syria [117] and Lebanon [118, 119].

3. Historical interpretation of the Erfurt substructure

Our genetic data support the presence of population structure in Erfurt, where one group had elevated levels of Eastern European-related ancestry. These findings may correspond to a documented cultural and linguistic division that existed within medieval AJ along a west/east axis in Central Europe [120]. The Western communities were referred to as Rhineland Jews due to the geographic location of the initial AJ settlements, which at the time was referred to as *Ashkenaz*. These Jews, who were known in medieval Rabbinic literature as "*bney hes*" (after their way of pronunciation of the eighth letter in the Hebrew alphabet [121]), were likely derived mainly from Jews inhabiting Northern France and Italy in the centuries immediately prior. They maintained a unique religious rite and a different set of given names from the Jews living to the East, and during the first centuries of the second Millennium spoke German dialects structurally similar to those of the Christian majority [120]. These communities may have been represented by Erfurt-ME, given the genetic similarity between Erfurt-ME and MAJ of Western European origin.

The Eastern Jews inhabited southeastern and eastern Germany, Austria, Bohemia, Moravia, and Silesia, with early major settlements being Regensburg, Prague, Magdeburg, and Halle. They were known in as "*bney khes*." During the first centuries of the second Millennium, the Hebrew geographic term of *Knaan* was applied to a large part of this area, with Old Czech being the main language spoken by local Jews. In the westernmost part of the *bney khes* area (covering Regensburg, Austria, and the western communities of eastern Germany) Jews spoke German dialects. Much less is known about the earlier roots of the *bney khes*, but possibilities include Italy, the Byzantine realm, and Jews living even further east. Eastern Jews may correspond to Erfurt-EU, given the Eastern European-related minor genetic ancestry in that group. This hypothesis naturally requires that Eastern AJ had previously admixed with local non-Jewish populations.

Studies of the names, dialects, and religious rite of modern AJ suggest that the Western and Eastern communities eventually merged and formed a single Ashkenazi culture, defined by a unified religious rite and the Yiddish language [120]. This is consistent with the near lack of genetic structure in modern AJ.

Erfurt might have been at the boundary between the two AJ communities [122]. The available lists of Jewish martyrs from Erfurt (1221) and neighboring Weißensee (1303) [123] show the presence of given names typical to Western AJ [124]. These Jews or their ancestors migrated to Thuringia from the Rhineland. Yet, in the sources from Erfurt from the second half of the 14th century, numerous Slavic given names show the presence there of Eastern AJ [124]. For some of these individuals, as we

described above (Section 2.2), their provenance from Bohemia, Moravia, and Silesia is explicitly indicated in the historical documents [125]. Others who have resettled in Erfurt came from surrounding towns in Thuringia and from Western Germany [125], and likely belonged to the Western community. Migration is also supported by the isotope analysis. These considerations may explain why our sampling of a single cemetery section was able to capture the medieval AJ substructure.

4. Timing demographic events in Ashkenazi history

Our modeling of shared haplotypes dated the onset of the AJ bottleneck to ≈40-45 generations ago, or approximately about 1000-1200 years ago. This period is well before the time in the late 11th century when the persecution of Jews in the Rhineland became endemic. The appearance of a bottleneck in the early stages of the AJ community formation could reflect the historical evidence that the original AJ settlers comprised only a few dozen families, which were not always welcome and lacked the benefit of a fully developed Jewish community [126].

Our models dated the onset of expansion of AJ to about 20-25 generations ago, or approximately about 500-700 years ago. This confirms historical research pointing towards a gradual demographic growth within the Jewish community in German lands. The growth is hard to quantify numerically, but, especially from the 1300s onwards, it appears to have been substantial, considering the rapid increase in the number of towns that accommodated Jewish communities [127].

In this work, we were unable to reliably estimate the dates of the historical admixture events of AJ in Europe. Our previous work inferred a minor post-bottleneck gene flow event from Eastern Europeans based on a depletion of EU ancestry in IBD segments [40] (as such segments are expected to descend from ancestors who lived during the bottleneck). However, with a model of a prolonged bottleneck (about 20 generations; Table S5), such a depletion may be observed also if the admixture event had happened late during the bottleneck. Our previous work estimated that admixture between Middle Eastern and European sources in AJ history occurred about 30 generations ago [40]. This date may be associated with the admixture event with Eastern Europeans. Unfortunately, our EAJ genomes did not provide additional insights, as we found that a state-of-the-art tool for admixture time inference (*DATES*) provided unreliable results under simulations of AJ-like demographic history (Section 8 above).

References

1. Harck, O., *Archäologische Studien zum Judentum in der europäischen Antike und dem zentraleuropäischen Mittelalter*. Schriftenreihe der Bet Tfila – Forschungsstelle. 2014, Petersberg: Michael Imhof.
2. Schütte, S. and M. Gechter, *Von der Ausgrabung zum Museum-Kölner Archäologie zwischen Rathaus und Praetorium: Ergebnisse und Materialien 2006-2012*. 2012, Köln: Stadt Köln.
3. Haverkamp, A., *The Beginning of Jewish Life North of the Alps with Comparative Glances at Italy (ca. 900-1100)*, in *"Diversi angoli di visuale" fra storia medievale e storia degli ebrei*, A.M. Pult Quaglia and A. Veronese, Editors. 2016, Pacani editore: Pisa. p. 85-102.
4. Heil, J., *Von Italien an den Rhein und zurück: Migration, soziale Mobilität und kultureller Wandel bei den aschkenasischen Juden (950–1500)*, in *Migration als soziale Herausförderung: Historische Formen solidarischen Handelns von der Antike bis zum 20. Jahrhundert*, J. Bahlcke, R. Leng, and P. Scholz, Editors. 2011, Steiner: Stuttgart. p. 101-122.
5. Heil, J., *From Venosa to Mainz? Considerations on the Origins of Jewish Life North of the Alps*, in *Medieval Ashkenaz. Papers in Honour of Alfred Haverkamp Presented at the 17th World Congress of Jewish Studies, Jerusalem 2017*, C. Clüse and J.R. Müller, Editors. 2021, Harrassowitz: Wiesbaden. p. 1-14.
6. Toch, M., *The economic history of European Jews: Late antiquity and early Middle Ages*. Études sur le judaïsme médiéval. 2013, Leiden: Brill.
7. Gusev, A., et al., *The architecture of long-range haplotypes shared within and across populations.* Mol Biol Evol, 2012. **29**(2): p. 473-86.
8. Guha, S., et al., *Implications for health and disease in the genetic signature of the Ashkenazi Jewish population.* Genome Biol, 2012. **13**(1): p. R2.
9. Kopelman, N.M., et al., *High-resolution inference of genetic relationships among Jewish populations.* Eur J Hum Genet, 2020. **28**(6): p. 804-814.
10. Prive, F., et al., *Portability of 245 polygenic scores when derived from the UK Biobank and applied to 9 ancestry groups from the same cohort.* Am J Hum Genet, 2022. **109**(1): p. 12-23.
11. Behar, D.M., et al., *No evidence from genome-wide data of a Khazar origin for the Ashkenazi Jews.* Hum Biol, 2013. **85**(6): p. 859-900.
12. Granot-Hershkovitz, E., et al., *A study of Kibbutzim in Israel reveals risk factors for cardiometabolic traits and subtle population structure.* Eur J Hum Genet, 2018. **26**(12): p. 1848-1858.
13. Gladstein, A.L. and M.F. Hammer, *Substructured Population Growth in the Ashkenazi Jews Inferred with Approximate Bayesian Computation.* Mol Biol Evol, 2019. **36**(6): p. 1162-1171.
14. Han, E., et al., *Clustering of 770,000 genomes reveals post-colonial population structure of North America.* Nat Commun, 2017. **8**: p. 14238.
15. Costa, M.D., et al., *A substantial prehistoric European ancestry amongst Ashkenazi maternal lineages.* Nat Commun, 2013. **4**: p. 2543.
16. Feder, J., et al., *Ashkenazi Jewish mtDNA haplogroup distribution varies among distinct subpopulations: lessons of population substructure in a closed group.* Eur J Hum Genet, 2007. **15**(4): p. 498-500.
17. Klitz, W., et al., *Genetic differentiation of Jewish populations.* Tissue Antigens, 2010. **76**(6): p. 442-58.
18. Durst, R., et al., *Recent origin and spread of a common Lithuanian mutation, G197del LDLR, causing familial hypercholesterolemia: positive selection is not always necessary to account for disease incidence among Ashkenazi Jews.* Am J Hum Genet, 2001. **68**(5): p. 1172-88.
19. Risch, N., et al., *Geographic distribution of disease mutations in the Ashkenazi Jewish population supports genetic drift over selection.* Am J Hum Genet, 2003. **72**(4): p. 812-22.
20. Altwasser, E., *Die Alte Synagogue*. Die mittelalterliche jüdische Kultur in Erfurt, ed. S. Ostritz. 2009, Weimar: Beier & Beran.

21. *Decision of the German Rabbinical Conference of November 1991*, in *Hamburg Key Documents on German-Jewish History*. 1991.
22. Reinhold, R.-H., *Juden und Christen im spätmittelalterlichen Erfurt. Abhängigkeiten, Handlungsspielräume und Gestaltung jüdischen Lebens in einer mitteleuropäischen Großstadt.* 2007, Universität Trier.
23. Lämmerhirt, M., *Die Anfänge der jüdischen Besiedlung in Thüringen.* Zeitschrift für Thüringische Geschichte, 2015. **69**: p. 57-91.
24. Weigelt, C.M., *Das Erfurter Pestpogrom 1349. Eine kritische Rekonstruktion.*, in *Die Erfurter jüdische Gemeinde im Spannungsfeld zwischen Stadt, Erzbischof und Kaiser*. 2016, Bussert & Stadeler: Jena/Quedlinburg. p. 30-122.
25. Lämmerhirt, M., *Migration von Juden in Thüringen, insbesondere nach 1349 und Mitte des 15. Jahrhunderts.*, in *Jüdische Geschichte in Thüringen. Strukturen und Entwicklungen vom Mittelalter bis ins 20. Jahrhundert*, H.-W. Hahn and M. Kreutzmann, Editors.
26. *Cronicae S. Petri Erfordensis Moderna*, in *Cronica S. Petri*, E. Holder-Egger, Editor. 1896: Hannover. p. 335-489.
27. Toch, M., *Siedlungsstruktur der Juden Mitteleuropas im Wandel von Mittelalter zur Neuzeit*, in *Juden in der christlichen Umwelt während des späten Mittelalters*, A. Haverkamp and F.-J. Ziwes, Editors. 1992, Duncker & Humblot: Berlin. p. 29-39.
28. Lämmerhirt, M., *Neuedition des Liber Judeorum der Stadt Erfurt*, in *Wohnen, beten, handeln. Das hochmittelalterliche jüdische Quartier ante pontem in Erfurt. Mit einer Neuedition des Liber Judeorum der Stadt Erfurt.*, B. Perlich, Editor. 2019, Michael Imhof: Petersberg. p. 297-399.
29. Lämmerhirt, M., *Juden in den wettinischen Herrschaftsgebieten: Recht, Verwaltung und Wirtschaft im Spätmittelalter*. Veröffentlichungen der Historischen Kommission für Thüringen. 2007, Köln/Weimar/Wien: Vandenhoeck & Ruprecht.
30. Lämmerhirt, M., *Die Schutzaufkündigung 1453 und das Ende der zweiten jüdischen Gemeinde*, in *Die Erfurter jüdische Gemeinde im Spannungsfeld zwischen Stadt, Erzbischof und Kaiser*. 2016, Bussert & Stadeler: Jena/Quedlinburg. p. 124-137.
31. *Letters in the Dust: The Epigraphy and Archaeology of Medieval Jewish Cemeteries*. 2022, Leuven: Peeters.
32. Flohr, S., *Die Bestattungen vom mittelalterlichen Judenfriedhof "Große Ackerhofsgasse" in Erfurt*, in *Der Friedhof. Archäologie – Geschichte - Anthropologie - Genetik - Grabsteine*, S. Ostritz, Editor., Thüringisches Landesamt für Denkmalpflege und Archäologie: Weimar.
33. Soe, M.J., et al., *Ancient DNA from latrines in Northern Europe and the Middle East (500 BC-1700 AD) reveals past parasites and diet.* PLoS One, 2018. **13**(4): p. e0195481.
34. Willmann, C., et al., *Oral health status in historic population: Macroscopic and metagenomic evidence.* PLoS One, 2018. **13**(5): p. e0196482.
35. Schuenemann, V.J., et al., *Targeted enrichment of ancient pathogens yielding the pPCP1 plasmid of Yersinia pestis from victims of the Black Death.* Proc Natl Acad Sci U S A, 2011. **108**(38): p. E746-52.
36. Alexander, D.H., J. Novembre, and K. Lange, *Fast model-based estimation of ancestry in unrelated individuals.* Genome Res, 2009. **19**(9): p. 1655-64.
37. Tian, J.Y., et al., *A genetic contribution from the Far East into Ashkenazi Jews via the ancient Silk Road.* Sci Rep, 2015. **5**: p. 8377.
38. Tibshirani, R., G. Walther, and T. Hastie, *Estimating the number of clusters in a data set via the gap statistic.* J. R, Stat. Soc. Series B Stat. Methodol., 2002. **63**: p. 411.
39. Gao, L.L., J. Bien, and D. Witten, *Selective Inference for Hierarchical Clustering.* arXiv, 2020. **2012.02936**.
40. Xue, J., et al., *The time and place of European admixture in Ashkenazi Jewish history.* PLoS Genet, 2017. **13**(4): p. e1006644.

41. McCarthy, S., et al., *A reference panel of 64,976 haplotypes for genotype imputation.* Nat Genet, 2016. **48**(10): p. 1279-83.
42. Price, A.L., et al., *Sensitive detection of chromosomal segments of distinct ancestry in admixed populations.* PLoS Genet, 2009. **5**(6): p. e1000519.
43. Antonio, M.L., et al., *Ancient Rome: A genetic crossroads of Europe and the Mediterranean.* Science, 2019. **366**(6466): p. 708-714.
44. Agranat-Tamir, L., et al., *The Genomic History of the Bronze Age Southern Levant.* Cell, 2020. **181**(5): p. 1146-1157 e11.
45. Veeramah, K.R., et al., *Population genomic analysis of elongated skulls reveals extensive female-biased immigration in Early Medieval Bavaria.* Proc Natl Acad Sci U S A, 2018. **115**(13): p. 3494-3499.
46. Narasimhan, V.M., et al., *The formation of human populations in South and Central Asia.* Science, 2019. **365**(6457).
47. Chintalapati, M., N. Patterson, and P. Moorjani, *The spatiotemporal patterns of major human admixture events during the European Holocene.* Elife, 2022. **11**.
48. Loh, P.R., et al., *Inferring admixture histories of human populations using linkage disequilibrium.* Genetics, 2013. **193**(4): p. 1233-54.
49. Henn, B.M., et al., *Characterizing the time dependency of human mitochondrial DNA mutation rate estimates.* Mol Biol Evol, 2009. **26**(1): p. 217-30.
50. Poznik, G.D., et al., *Sequencing Y chromosomes resolves discrepancy in time to common ancestor of males versus females.* Science, 2013. **341**(6145): p. 562-5.
51. Carmi, S., et al., *Sequencing an Ashkenazi reference panel supports population-targeted personal genomics and illuminates Jewish and European origins.* Nat. Commun., 2014. **5**: p. 4835.
52. Ringbauer, H., G. Coop, and N.H. Barton, *Inferring Recent Demography from Isolation by Distance of Long Shared Sequence Blocks.* Genetics, 2017. **205**(3): p. 1335-1351.
53. Palamara, P.F., et al., *Length distributions of identity by descent reveal fine-scale demographic history.* Am J Hum Genet, 2012. **91**(5): p. 809-22.
54. Ralph, P. and G. Coop, *The geography of recent genetic ancestry across Europe.* PLoS Biol, 2013. **11**(5): p. e1001555.
55. Carmi, S., et al., *A renewal theory approach to IBD sharing.* Theor. Popul. Biol., 2014. **97**: p. 35-48.
56. Weinryb, B.D., *The Jews of Poland: A Social and Economic History of the Jewish Community in Poland from 1100 to 1800*. 1972: The Jewish Publication Society of America.
57. Wasserman, L., *All of Statistics: A Concise Course in Statistical Inference*. 2004, New York: Springer.
58. Ringbauer, H., J. Novembre, and M. Steinrucken, *Parental relatedness through time revealed by runs of homozygosity in ancient DNA.* Nat Commun, 2021. **12**(1): p. 5425.
59. Fenner, J.N., *Cross-cultural estimation of the human generation interval for use in genetics-based population divergence studies.* Am J Phys Anthropol, 2005. **128**(2): p. 415-23.
60. Helgason, A., et al., *A populationwide coalescent analysis of Icelandic matrilineal and patrilineal genealogies: evidence for a faster evolutionary rate of mtDNA lineages than Y chromosomes.* Am J Hum Genet, 2003. **72**(6): p. 1370-88.
61. Moorjani, P., et al., *A genetic method for dating ancient genomes provides a direct estimate of human generation interval in the last 45,000 years.* Proc Natl Acad Sci U S A, 2016. **113**(20): p. 5652-7.
62. Tremblay, M. and H. Vezina, *New estimates of intergenerational time intervals for the calculation of age and origins of mutations.* Am J Hum Genet, 2000. **66**(2): p. 651-8.
63. Wang, R.J., et al., *Human generation times across the past 250,000 years.* BioRxiv, 2021: p. 2021.09.07.459333v1.

64. Grossman, A., *Pious and rebellious: Jewish women Medieval Europe*. 2004, Waltham, MA: Brandeis University Press.
65. Gutenkunst, R.N., et al., *Inferring the joint demographic history of multiple populations from multidimensional SNP frequency data.* PLoS Genet, 2009. **5**(10): p. e1000695.
66. Palamara, P.F., *ARGON: fast, whole-genome simulation of the discrete time Wright-fisher process.* Bioinformatics, 2016. **32**(19): p. 3032-4.
67. Kedar-Barnes, I. and P. Rozen, *The Jewish people: their ethnic history, genetic disorders and specific cancer susceptibility.* Fam Cancer, 2004. **3**(3-4): p. 193-9.
68. Kuchenbaecker, K.B., et al., *Risks of Breast, Ovarian, and Contralateral Breast Cancer for BRCA1 and BRCA2 Mutation Carriers.* JAMA, 2017. **317**(23): p. 2402-2416.
69. Laitman, Y., et al., *Haplotype analysis of the 185delAG BRCA1 mutation in ethnically diverse populations.* Eur J Hum Genet, 2013. **21**(2): p. 212-6.
70. Thaler, A., et al., *The LRRK2 G2019S mutation as the cause of Parkinson's disease in Ashkenazi Jews.* J Neural Transm (Vienna), 2009. **116**(11): p. 1473-82.
71. Lesage, S., et al., *Parkinson's disease-related LRRK2 G2019S mutation results from independent mutational events in humans.* Hum Mol Genet, 2010. **19**(10): p. 1998-2004.
72. Ben El Haj, R., et al., *Evidence for prehistoric origins of the G2019S mutation in the North African Berber population.* PLoS One, 2017. **12**(7): p. e0181335.
73. Diaz, G.A., et al., *Gaucher disease: the origins of the Ashkenazi Jewish N370S and 84GG acid beta-glucosidase mutations.* Am J Hum Genet, 2000. **66**(6): p. 1821-32.
74. Peretz, H., et al., *The two common mutations causing factor XI deficiency in Jews stem from distinct founders: one of ancient Middle Eastern origin and another of more recent European origin.* Blood, 1997. **90**(7): p. 2654-9.
75. Toncheva, D., et al., *Spatio-temporal dynamics of pathogenic variants associated with monogenic disorders reconstructed with ancient DNA.* PLoS One, 2022. **17**(6): p. e0269628.
76. Goldstein, D.B., et al., *Age estimates of two common mutations causing factor XI deficiency: recent genetic drift is not necessary for elevated disease incidence among Ashkenazi Jews.* Am J Hum Genet, 1999. **64**(4): p. 1071-5.
77. Yepiskoposyan, L. and A. Harutyunyan, *Population genetics of familial Mediterranean fever: a review.* Eur J Hum Genet, 2007. **15**(9): p. 911-6.
78. Jalkh, N., et al., *Familial Mediterranean Fever in Lebanon: founder effects for different MEFV mutations.* Ann Hum Genet, 2008. **72**(Pt 1): p. 41-7.
79. Consortium, T.I.F., *Ancient missense mutations in a new member of the RoRet gene family are likely to cause familial Mediterranean fever. The International FMF Consortium.* Cell, 1997. **90**(4): p. 797-807.
80. Lerer, I., et al., *Cystic fibrosis mutations delta F508 and G542X in Jewish patients.* J Med Genet, 1992. **29**(2): p. 131-3.
81. Zelinger, L., et al., *A missense mutation in DHDDS, encoding dehydrodolichyl diphosphate synthase, is associated with autosomal-recessive retinitis pigmentosa in Ashkenazi Jews.* Am J Hum Genet, 2011. **88**(2): p. 207-15.
82. Herrera, W., et al., *Retinal disease in Usher syndrome III caused by mutations in the clarin-1 gene.* Invest Ophthalmol Vis Sci, 2008. **49**(6): p. 2651-60.
83. Ekstein, J., et al., *Mutation frequencies for glycogen storage disease Ia in the Ashkenazi Jewish population.* Am J Med Genet A, 2004. **129A**(2): p. 162-4.
84. Pedersen, C.B., et al., *The ACADS gene variation spectrum in 114 patients with short-chain acyl-CoA dehydrogenase (SCAD) deficiency is dominated by missense variations leading to protein misfolding at the cellular level.* Hum Genet, 2008. **124**(1): p. 43-56.
85. Enattah, N.S., et al., *Identification of a variant associated with adult-type hypolactasia.* Nat Genet, 2002. **30**(2): p. 233-7.

86. Eiberg, H., et al., *Blue eye color in humans may be caused by a perfectly associated founder mutation in a regulatory element located within the HERC2 gene inhibiting OCA2 expression.* Hum Genet, 2008. **123**(2): p. 177-87.
87. Han, J., et al., *A genome-wide association study identifies novel alleles associated with hair color and skin pigmentation.* PLoS Genet, 2008. **4**(5): p. e1000074.
88. Immel, A., et al., *Analysis of Genomic DNA from Medieval Plague Victims Suggests Long-Term Effect of Yersinia pestis on Human Immunity Genes.* Mol Biol Evol, 2021. **38**(10): p. 4059-4076.
89. Raxter, M.H., B.M. Auerbach, and C.B. Ruff, *Revision of the Fully technique for estimating statures.* American Journal of Physical Anthropology, 2006. **130**: p. 374.
90. Pearson, K., *On the reconstruction of stature of prehistoric races. Mathematical contributions to the theory of evolution.* Philos. Trans. R. Soc. A, 1899. **192**: p. 169.
91. Yengo, L., et al., *Meta-analysis of genome-wide association studies for height and body mass index in approximately 700000 individuals of European ancestry.* Hum Mol Genet, 2018. **27**(20): p. 3641-3649.
92. Chang, C.C., et al., *Second-generation PLINK: rising to the challenge of larger and richer datasets.* Gigascience, 2015. **4**: p. 7.
93. Cox, S.L., et al., *Predicting skeletal stature using ancient DNA.* Am. J. Biol. Anthropol., 2022. **177**: p. 162.
94. Marciniak, S., et al., *An integrative skeletal and paleogenomic analysis of stature variation suggests relatively reduced health for early European farmers.* Proc Natl Acad Sci U S A, 2022. **119**(15): p. e2106743119.
95. Grossman, A., *Emigration and settlement in Jewish and general history*, A. Shinan, Editor. 1982, Shazar: Jerusalem. p. 109-128.
96. Fleischer, E., *Prayer And Piyut in the Worms Mahzor*, in *Mahzor Worms: Introductory volume*, M. Beit-Arié, Editor. 1985, Jerusalem National and University Library: Jerusalem. p. 36-78.
97. Kahan Newman, Z., *The Jewish sound of speech: Talmudic chant, Yiddish intonation and the origins of early Ashkenaz.* Jewish Quart. Rev., 2000. **90**: p. 293-336.
98. Rutgers, L.V. and S. Bradbury, *The Diaspora, c. 235–638*, in *The Late Roman-Rabbinic Period*, S.T. Katz, Editor. 2008, Cambridge University Press: Cambridge. p. 492-518.
99. Bonfil, R., *History and folklore in a medieval Jewish chronicle: The Family Chronicle of Aḥimaʿaz ben Paltiel*. Studies in Jewish History and Culture. 2009, Leiden: Brill.
100. Idel, M., *Kabbalh in Italy 1280-1510: A survey*. 2011, New Haven/London: Yale University Press.
101. Haverkamp, A., *Chapter 9 - Germany*, in *The Middle Ages: The Christian World, The Christian World*, R. Chazan, Editor. 2018, Cambridge University Press: Cambridge. p. 239-281.
102. Brody, R., *On the dissemination of the Babylonian Talmud and the origins of Ashkenazi Jewry.* Jewish Quart. Rev., 2019. **109**: p. 265-288.
103. Lehnardt, A., *Die Kalonymiden - von Lucca an den Rhein*, in *Es war eine berühmte Stadt...: Mainzer mittelalterliche Erzählungen und ihre Deutung*, W. Dobras, Editor. 2016, Bistum Mainz & Echter: Mainz/Würzburg. p. 171-190.
104. Trebilco, P., *Jewish communities in Asia Minor*, in *Inscriptiones Judaicae Orientis: Volume I: Eastern Europe*, D. Noy, A. Panayotnov, and H. Bloedhorn, Editors. 1991, Mohr Siebeck: Germany.
105. Rutgers, L.V., *Interaction and its limits : some notes on the Jews of Sicily in late antiquity.* Zeitschrift für Papyrologie und Epigraphik, 1997. **115**: p. 245.
106. Brundage, J.A., *Intermarriage between Christians and Jews in Medieval Canon Law.* Jew. Hist., 1988. **3**: p. 25-40.
107. Nirenberg, D., *Conversion, Sex, and Segregation: Jews and Christians in Medieval Spain.* Am. Hist. Rev., 2002. **107**: p. 1065-1093.
108. Raveane, A., et al., *Population structure of modern-day Italians reveals patterns of ancient and archaic ancestries in Southern Europe.* Sci Adv, 2019. **5**(9): p. eaaw3492.

109. Moorjani, P., et al., *The history of African gene flow into Southern Europeans, Levantines, and Jews.* PLoS Genet, 2011. **7**(4): p. e1001373.
110. Hellenthal, G., et al., *A genetic atlas of human admixture history.* Science, 2014. **343**(6172): p. 747-51.
111. Haber, M., et al., *Genome-wide diversity in the levant reveals recent structuring by culture.* PLoS Genet, 2013. **9**(2): p. e1003316.
112. Fernandes, V., et al., *Genome-Wide Characterization of Arabian Peninsula Populations: Shedding Light on the History of a Fundamental Bridge between Continents.* Mol Biol Evol, 2019. **36**(3): p. 575-586.
113. Mineta, K., et al., *Population structure of indigenous inhabitants of Arabia.* PLoS Genet, 2021. **17**(1): p. e1009210.
114. Schuenemann, V.J., et al., *Ancient Egyptian mummy genomes suggest an increase of Sub-Saharan African ancestry in post-Roman periods.* Nat Commun, 2017. **8**: p. 15694.
115. Zidan, J., et al., *Genotyping of geographically diverse Druze trios reveals substructure and a recent bottleneck.* Eur. J. Hum. Genet., 2015. **23**(8): p. 1093-9.
116. Markus, B., I. Alshafee, and O.S. Birk, *Deciphering the fine-structure of tribal admixture in the Bedouin population using genomic data.* Heredity (Edinb), 2014. **112**(2): p. 182-9.
117. Srigyan, M., et al., *Bioarchaeological evidence of one of the earliest Islamic burials in the Levant.* Commun Biol, 2022. **5**(1): p. 554.
118. Haber, M., et al., *A Transient Pulse of Genetic Admixture from the Crusaders in the Near East Identified from Ancient Genome Sequences.* Am J Hum Genet, 2019. **104**(5): p. 977-984.
119. Haber, M., et al., *A Genetic History of the Near East from an aDNA Time Course Sampling Eight Points in the Past 4,000 Years.* Am J Hum Genet, 2020. **107**(1): p. 149-157.
120. Beider, A., *Origins of Yiddish Dialects*. 2015, Oxford: Oxford University Press.
121. Katz, D., *East and West, khes and shin and the origins of Yiddish*, in *Studies in Jewish culture in honour of Chone Shmeruk*, I. Bartal, E. Mendelsohn, and C. Turniansky, Editors. 1993, The Zalman Shazar Center for Jewish History: Jerusalem. p. 9-37.
122. Weinreich, M., *History of the Yiddish language*. 1 ed. Vol. 1. 2008, United States: Yale University Press.
123. Salfeld, S., *Das Martyrologium des Nürnberger Memorbuches*. Quellen zur Geschichte der Juden in Deutschland. Vol. 3. 1898, Berlin: Simion.
124. Beider, A., *A Dictionary of Ashkenazic Given Names: Their Origins, Structure, Pronunciation, and Migrations*. 2001: Avotaynu.
125. Süssmann, A., *Das Erfurter Judenbuch: 1357-1407*. Mitteilungen des Gesamtarchivs der deutschen Juden. Vol. 5. 1915: G. Fock.
126. Toch, M., *Die Juden im mittelalterlichen Reich*. Enzyklopädie deutscher Geschichte. 2013, Munich: Oldenbourg.
127. Toch, M., *The Formation of a Diaspora: The Settlement of Jews in the Medieval German Reich.* Aschkenas, 1997. **7**: p. 55-78.
128. Behar, D.M., et al., *The matrilineal ancestry of Ashkenazi Jewry: portrait of a recent founder event.* Am J Hum Genet, 2006. **78**(3): p. 487-97.

Supplementary Tables

Left population 1	Left population 2	P-value European populations	P-value Middle Eastern populations
Erfurt Ashkenazi Jews	Modern Ashkenazi Jews	0.15	0.38
Erfurt-EU	Modern Ashkenazi Jews	3.2e-06	0.14
Erfurt-ME	Modern Ashkenazi Jews	0.031	0.63
Erfurt Ashkenazi Jews	Turkish Jews	1.8e-10	0.18
Erfurt-EU	Turkish Jews	4.0e-18	0.085
Erfurt-ME	Turkish Jews	0.0081	0.60
Turkish Jews	Modern Ashkenazi Jews	2.9e-14	0.39
Erfurt Ashkenazi Jews	Germans	2.1e-81	0.00029
Germans	Modern Ashkenazi Jews	8.4e-187	2.0e-8
Erfurt Ashkenazi Jews	South-Italians	0.00020	0.030
South-Italians	Modern Ashkenazi Jews	8.1e-7	0.012

Table S1. *qpWave* results. Related to Figure 3. Each line presents the P-value for one *qpWave* test. The reference European populations were modern Russian, Norwegian, French, Spanish, Bulgarian, and Italian_North, with Primate_Chimp as an outgroup (first right population). The reference Middle Eastern populations were BedouinA, Lebanese, Jordanian, and Druze, with Primate_Chimp as an outgroup. Entries with P>0.05 are highlighted in dark green, and entries with 0.01≤P≤0.05 in light green. The only case with P>0.05 with respect to European populations is when the left populations are Erfurt Ashkenazi Jews (EAJ) and modern Ashkenazi Jews (MAJ). When Erfurt is replaced by Erfurt-ME or Erfurt-EU, the P-value is smaller, reflecting the differences in ancestry between each EAJ sub-group and MAJ. In the other tests, we replaced MAJ or EAJ with Sephardi (Turkish) Jews or with non-Jewish Italians and Germans. P-values were very small except when comparing Erfurt-ME and Sephardi Jews.

		Proportion of Eastern European ancestry				
		**1%**	**2%**	**4%**	**6%**	**8%**
**Admixture time (generations before present)**	**20**	0.36	0.34	0.23	0.034	0.0014
	**15**	0.33	0.091	0.0010	1.7e-07	2.2e-10
	**10**	0.040	0.040	0.0060	4.9e-05	2.6e-07
	**5**	0.21	0.087	0.0018	3.5e-06	2.1e-09

Table S2. Determining the degree of endogamy in AJ in the past ≈700 years. Related to Figure 3. We used simulations to quantify the maximal degree of gene flow from Eastern Europeans into a group of modern AJ such that this admixed group will remain consistent with being a clade with unadmixed AJ. Our unadmixed group was the modern AJ dataset used for the original *qpWave* analyses. For the admixed group, we used $n = 30$ modern AJ genomes that were not used in the original analysis (sample size selected to match the size of the Erfurt sample; STAR Methods). In the admixed group, we replaced a given proportion of the genome (columns) with haplotypes from Eastern European sources (STAR Methods). The haplotype lengths were determined based on the assumed admixture times (rows; STAR Methods). Each entry in the table shows the P-value for a *qpWave* test comparing the admixed and unadmixed groups with respect to European populations (as in Table S1; STAR Methods). Cells with P>0.05 are highlighted in green. The results suggest an upper bound of about 2-4% on the degree of Eastern European gene flow separating modern and Erfurt AJ.

Middle Eastern source	Transversion SNPs (main analysis)			Robustness test: all available SNPs			Robustness test: different outgroup		
	North Italian	South Italian	Greek	North Italian	South Italian	Greek	North Italian	South Italian	Greek
**Druze**	0.0016	0.0031	0.00012	6.9e-9	9.4e-10	1.2e-13	0.0027	0.12	0.00022
**Egyptian**	1.4e-14	5.4e-6	3.1e-6	0	4.1e-17	2.0e-19	2.3e-12	0.027	0.017
**Bedouin A**	1.7e-7	0.0042	0.0036	2.9e-16	2.0e-6	9.2e-7	2.8e-6	0.15	0.098
**Bedouin B**	0.022	0.21	0.091	0.00068	0.026	0.0080	0.057	0.45	0.21
**Palestinian**	0.0021	0.058	0.020	3.7e-6	0.0020	0.00015	0.0074	0.36	0.12
**Lebanese**	0.086	0.32	0.046	0.037	0.15	0.00046	0.15	0.56	0.077
**Jordanian**	0.0039	0.13	0.060	0.00014	0.039	0.0041	0.014	0.41	0.18
**Syrian**	0.019	0.21	0.021	0.0064	0.061	0.00026	0.053	0.47	0.058
**Saudi**	0.36	0.15	0.14	0.094	0.18	0.022	0.30	0.60	0.25
**Levant**	0.0090	0.070	0.014	0.00024	0.0013	9.5e-6	0.035	0.41	0.10
**Levant + Cyprus**	0.015	0.081	0.016	0.00070	0.0016	1.2e-5	0.052	0.43	0.11

Table S3. *qpAdm* P-values for models with EAJ as the target group. Related to Figure 3. In all models, EAJ was the target group and there were three source populations: Middle Eastern, Southern European, and Eastern European. The rows represent the Middle Eastern source in each model and the columns represent the Southern European source. The Eastern European source in all models is Russians. Models with $P>0.05$ are highlighted in green, and models with $0.01 \le P \le 0.05$ in light green. The three columns under "transversions SNPs" present the results that were described in the main text, where we used only transversions to avoid bias due to ancient DNA damage. The next three columns ("all available SNPs") present the results of the same models when all SNPs were used. The next three columns ("different outgroup") present the results of the same models when we set Ami as the outgroup instead of Mbuti (using only transversions). In models with "Levant" as the Middle Eastern source we grouped Palestinian, Jordanian, Druze, Bedouin A, Bedouin B, Syrian, and Lebanese as a single source population. In models with "Levant + Cyprus" we added Cypriot to the Levant source. Overall, a South-Italian source was more plausible than a North-Italian or a Greek source, and Lebanese, Saudi, Syrian, Jordanian, and Bedouin B were the more likely Middle Eastern sources.

Haplogroup	Freq. in modern AJ	Freq. in Erfurt	Freq. in Erfurt-ME	Freq. in Erfurt-EU	Freq. in low-coverage samples
K1a1b1a	20%	11/31	7/13	2/10	2/8
N1b2	9%	1/31	1/13	0/10	0/8
K1a9	6%	2/31	1/13	0/10	1/8
K2a2a1	5%	0			

Table S4. Ashkenazi Jewish mitochondrial DNA founder lineages. Related to STAR Methods. Previous studies showed that modern AJ carry four founder lineages, with a cumulative frequency of 40% [128]. We compared the frequencies of these lineages in modern AJ [15] and Erfurt. We excluded the two children of family A since their mother is also in the sample. The frequency of K1a1b1a in Erfurt (35%) was significantly higher than in modern AJ (P=0.041; two-tailed binomial test (binom.test in *R*)). The frequency of K1a1b1a in Erfurt-ME (7/13=54%) was higher than the frequency in Erfurt-EU (2/10=20%), but given the small sample sizes, the difference was not statistically significant (P=0.20; two-tailed Fisher's exact test). The combined count of N1b2 (also called N1b1b1), K1a9, and K2a2a1 carriers (3/31; 9.7%) was lower in EAJ than expected based on modern AJ frequencies (20%), but again the result was not statistically significant (P=0.18; two-tailed binomial test).

	A	B	C	D	E	F	G	H
	**Modern IBD, variable bottleneck length**	**Modern IBD, single-generation bottleneck**	**Modern ROH**	**Consanguinity**	**Narrower bottleneck**	**Longer bottleneck**	**Joint likelihood**	**Two-population model**
$\boldsymbol{N_a}$	47,961 [45,922 - 72,585]	10,010	4,671	47,961	5,042	47,961	50,000	50,000 [50,000 - 97,465.5]
$\boldsymbol{N_b}$	1,563 [1,363.5 - 1,750.5]	480	1,295	1,563	529	1,563	905	627 [355 - 958]
$\boldsymbol{N_c}$	363,484 [-3,831,093.0 - 545,656.5]	157,498	10^7	363,483	10^7	169,092	10^7	1,652,527 [-6,694,946 - 2,456,011]
$\boldsymbol{T_b}$	41 [39 - 43]	31	30	41	41	74	51	46 [36 - 58]
$\boldsymbol{d}$	20 [15 - 24]	1 (fixed)	11	20	15	48	25	22 [9 - 35.5]
				$\boldsymbol{\alpha} = 0.22$				$\boldsymbol{f} = 0.52$ [0.41 - 0.99]

Table S5. Demographic models for the AJ founder event. Related to Figure 4. The table presents the parameters we inferred for various demographic models of AJ history. In all models, the population has been of constant effective size N_a (diploids) until T_b generations before present, and the population has grown exponentially starting $T_b - d$ generations ago and until reaching present size N_c. In the single-population models (all models except (H); Figure 4A), the population size has been N_b for d generations, starting T_b generations ago. In the two-population model ((H); Figure 4E), the population has split T_b generations ago into one population of size N_b (representing EAJ) and another of size $N_a - N_b$. After d generations, these two populations merged with proportions f and $(1 - f)$, respectively. In the consanguinity model (D), we assume that a proportion α of the EAJ individuals were born to parents who were first cousins.

In all models, we estimated the parameters numerically by maximizing the composite-likelihood of observing the given number of segments in each length bin (STAR Methods; Data S1, section 12). In some models, the maximum likelihood was obtained when some parameters were at the boundary of the search space (e.g., $N_a = 50k$ and $N_c = 10^7$). For models (A) and (H), we computed 95% confidence intervals (shown under each point estimate) using parametric bootstrap by resampling the segment counts per bin (STAR Methods; Data S1, section 12).

In models (A) and (B), we inferred the demographic parameters based on modern IBD sharing. In (A), we inferred the bottleneck duration (d) from the data, while in (B), we fixed it to 1. In the modern

ROH model (C), we estimated the model parameters based on ROH segments inferred in modern AJ. In the consanguinity model (D), we fixed all parameters that were inferred in model (A), and used ROH segments in the EAJ genomes to infer α, the proportion of EAJ individuals whose parents were first cousins. In the narrower bottleneck model (E), we fixed T_b from model (A), and inferred the population sizes N_a and N_b based on ROH segments in EAJ. We then fixed N_a and N_b to their inferred values, and inferred d and N_c using modern IBD. In the longer bottleneck model (F), we fixed N_a and N_b from model (A), and inferred T_b based on ROH in EAJ. As in (E), we then inferred d and N_c using the modern IBD data. In the joint likelihood model (G), we inferred the demographic parameters based on both ancient and modern data. Finally, in the two-population model (H), we inferred all parameters based on both ancient and modern data, including f, the proportion of modern AJ lineages that descend from the EAJ-like population. For complete details, see STAR Methods and Data S1, section 12.
