# Supplementary material for: Genome-wide data from medieval German Jews show that the Ashkenazi founder event pre-dated the 14th century
Source: Cell. Author manuscript; Available in PMC 2022 Dec 27. (PMC9793425; doi:10.1016/j.cell.2022.11.002)
Supplement: 1 [file NIHMS1852590-supplement-1.pdf]

CellPress
OPEN ACCESS

Supplemental figures

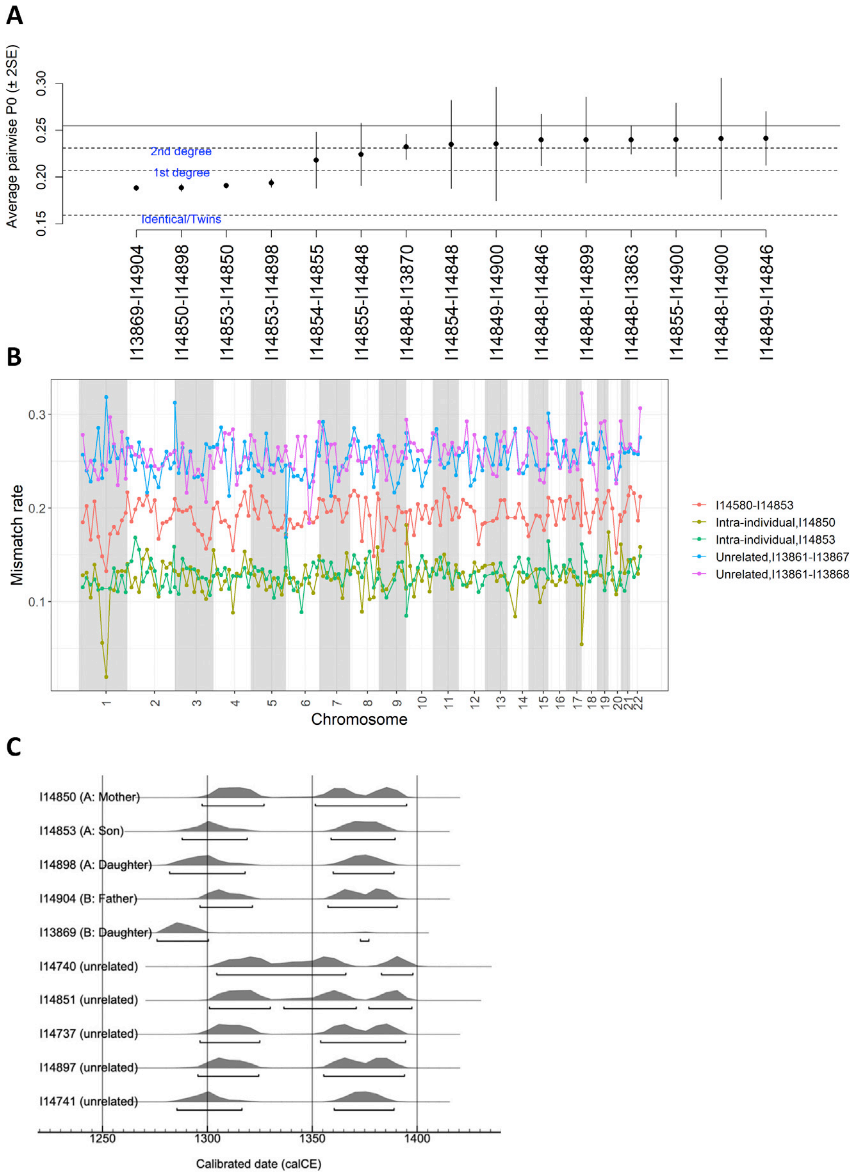

Figure S1. Genetic families and radiocarbon dating, related to Figure 1

(A) The output of *READ* for the first 15 pairs of EAJ individuals with the lowest proportion of non-matching alleles (P0). Each vertical line corresponds to a single pair. Seven of the pairs have a point estimate of having either a first- or a second-degree relationship. The y axis shows the mean P0 (across 1Mb genomic windows) between pairs of individuals. The horizontal solid line corresponds to the median P0 in the entire Erfurt sample, including pairs that are not shown. The horizontal dashed lines correspond to the cutoffs for first-degree relatives, second-degree relatives, and unrelated individuals (Monroy Kuhn et al., 2018). The vertical lines for each pair represent two standard errors of the mean (across genomics windows). Two pairs of individual, I14855 and I14854, and I14855 and I14848, were estimated to be second-degree relatives, although the confidence intervals also include a first-degree relationship and no relationship. The value of P0 for I14854 and I14848 was slightly above the cutoff for a second-degree relationship. All three samples had low coverage (<40 k SNPs). (B) The mismatch rate along the genome (in blocks of 20 Mb) between I14850 and I14853 is shown in red. For comparison, we show the mismatch rate for two pairs of unrelated individuals (blue and pink; legend) and the mismatch rate between two sets of randomly selected reads from the same individual (I14850 or I14853 in yellow and green, respectively). Data from blocks with fewer than 100 covered SNPs is not shown. The mismatch rate between I14850 and I14853 is intermediate between that of the unrelated individuals and the within-individual comparisons, indicating that they share exactly one chromosome along their entire genome, as

(legend continued on next page)

expected for a parent-child relationship. (C) The output of OxCal for ten samples that underwent radiocarbon dating. For each individual, the underlying bars denote intervals that have a cumulative probability of 95.4%. The estimated dates are almost equally likely to pre-date or post-date the 1349 pogrom. One individual — I13869 — was inferred to be much more likely to date to the end of the 13th century. However, there was a small peak in the late 14th century, and this individual is the daughter of I14904 (Figure 1B), who is more likely to date to the 14th century. This implies that I13869 might also date to the 14th century, despite this event having a smaller probability.

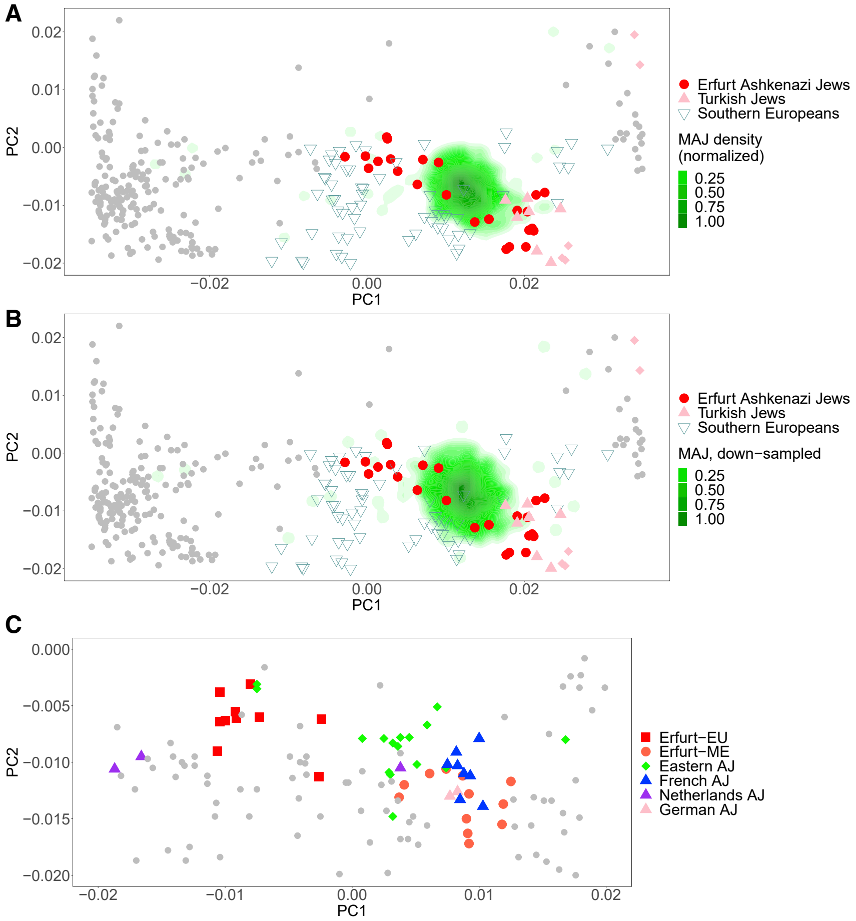

Figure S2. PCA plots of modern and ancient AJ, related to Figure 2

We learned the PCs using all West-Eurasian individuals from the Human Origins dataset. The figure shows just the subset of the space relevant for within-AJ structure. (A) Projection of a large modern AJ sample onto PC space. The plot is similar to the inset of Figure 2 of the main text, with two differences. (1) We projected both Erfurt and modern AJ individuals. (2) We did not include the seven modern AJ samples that were part of the Human Origins dataset. For modern AJ, we used The Ashkenazi Genome Consortium (TAGC) dataset (n = 544), down-sampled to the approximately 470k Human Origins SNPs. As the modern AJ sample is very large, we do not plot individual points but rather their density (STAR Methods). As in Figure 2, the Erfurt genomes show higher variability on the PC1 axis (the European/Middle Eastern cline) than modern AJ. (B) To demonstrate that the higher variability in EAJ is not due to their lower coverage compared to MAJ, we down-sampled each of the TAGC individuals to match the SNPs covered in an EAJ genome. We implemented this by arbitrarily ordering (randomly selected) 525 MAJ and the 25 non-low-coverage EAJ genomes, and sequentially matching MAJ and EAJ samples, cycling over the EAJ samples until covering all modern genomes. For each down-sampled MAJ genome, we further used a single, randomly-selected allele. Down-sampling did not qualitatively change the results. (C) To test whether the two subgroups of Erfurt correspond to modern AJ of Eastern European or Western European origin, we merged the Erfurt data with that of Behar et al. (2013). The merged dataset included about 246k SNPs. We projected both EAJ and MAJ onto the PC plane. The results show that French and German MAJ overlap with Erfurt-ME and that Eastern MAJ have more EU ancestry, although most of them still cluster primarily next to Erfurt-ME.

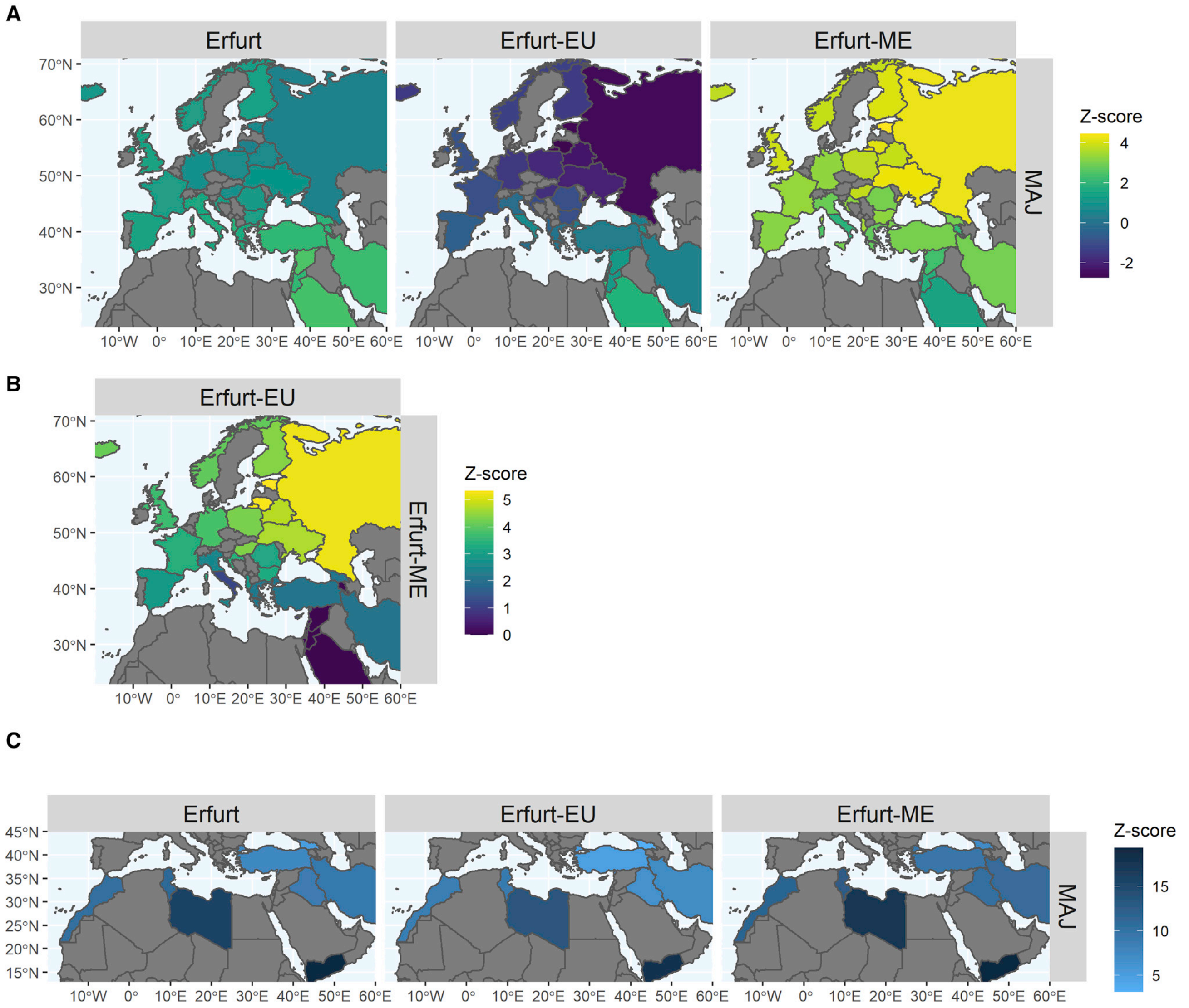

Figure S3. Results of the f_4-statistics tests, related to Figure 3

(A) Tests of the form f_4(MAJ, EAJ; X, chimp), where X represents any non-Jewish West-Eurasian population. Each country on the map was colored based on the *Z* score for deviation from zero of the f_4-statistic when replacing X with the local population in the Human Origins dataset. Gray represents countries that were not tested. In the middle and right columns, EAJ were replaced with Erfurt-EU and Erfurt-ME, respectively. (B) Tests of the form f_4(Erfurt-EU, Erfurt-ME; X, chimp). Here too, X represents any non-Jewish West-Eurasian population. (C) Tests of the form f_4(MAJ, X; EAJ, chimp), where X represents Jewish non-Ashkenazi populations. The location of each Jewish population on the map is represented by its origin in the diaspora. In the middle and right columns, EAJ were replaced with Erfurt-EU and Erfurt-ME, respectively. In all analyses, the *Z* score was positive and >3, indicating that MAJ is the closest Jewish population to EAJ.

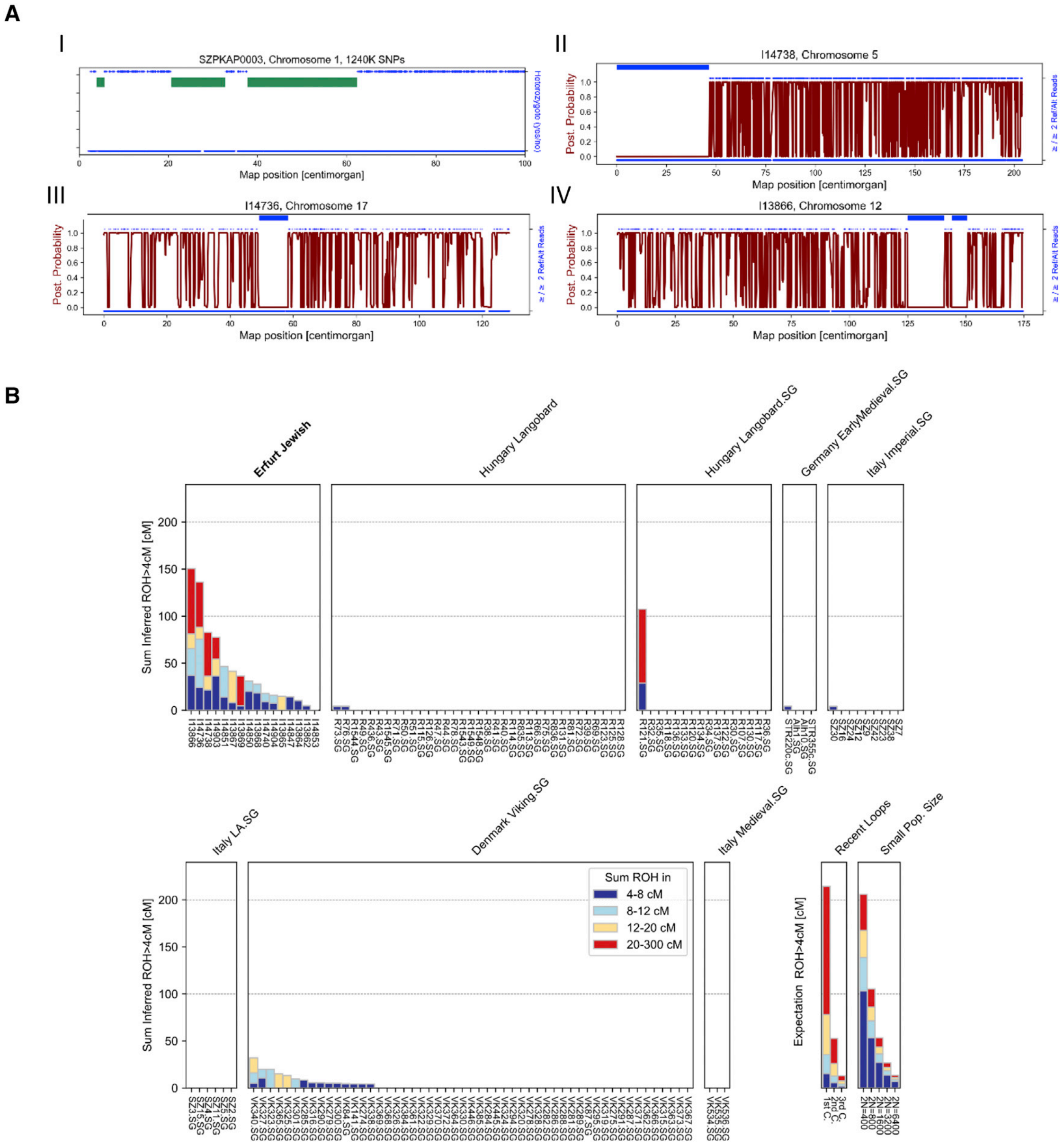

Figure S4. Visual inspection of runs of homozygosity (ROH) segments in modern and ancient genomes and a comparison to ancient European populations, related to Figure 4

(A) Examples of inferred ROH segments in one modern and three ancient AJ. Panel I demonstrates the inferred ROH segments (green bars, called with *bcftools/ROH*; STAR Methods) along a subset of chr1 in one modern AJ individual. We considered only bi-allelic SNPs included in the "1240" SNP panel. Blue dots at the top (bottom) of the panel show the positions of heterozygous (homozygous) sites. The inferred ROH segments are depleted of heterozygous sites. Panels II–IV demonstrate the inferred ROH segments (blue bars, inferred using hapROH; STAR Methods) in three chromosomes from three Erfurt individuals. The red lines show the posterior ("post.") probability estimated by hapROH that a SNP is in a non-ROH state given the data. The blue dots show sites that were covered by at least one read (STAR Methods). A dot is plotted at the top of the panel whenever the reads covered both alleles, which suggests heterozygosity. The inferred ROH segments are again depleted of these putatively heterozygous sites. (B) ROH levels across European populations from the past two millennia. Each bar represents one individual, and individuals are grouped by population labels. We show the sum of the lengths of ROHs in four length bins (see legend). On the bottom right, we demonstrate the expected sum of ROH lengths for individuals whose parents are close relatives (first, second, and third cousins; "recent loops"), as well as for individuals from a population of a given constant effective size (*N* is in number of diploid individuals; "small pop. size"). See (Ringbauer et al., 2021) for details. The Erfurt samples have substantially longer ROHs compared to all other populations. SG: shotgun sequencing. The Hungary Langobard data (SNP enrichment and SG) is from (Amorim et al., 2018). The Germany Early Medieval data is from (Veeramah et al., 2018). The Italy Imperial, Late Antiquity (LA), and Medieval data is from (Antonio et al., 2019). The Denmark Viking data is from (Margaryan et al., 2020).

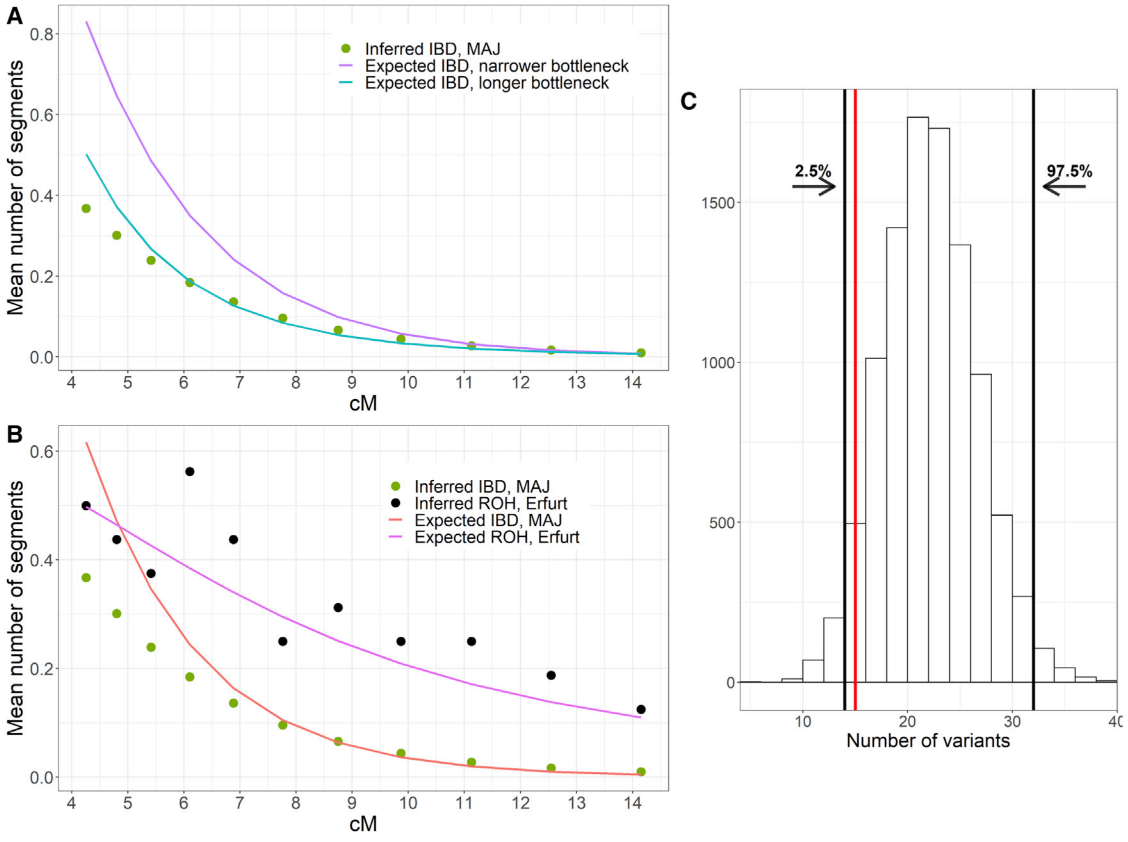

Figure S5. Evaluating demographic models and simulations of founder alleles, related to Figure 4

(A) and (B). Comparing predictions from demographic models for modern and Erfurt AJ vs observed data. (A) We plot the mean number of IBD segments (per pair of haploid genomes) across length bins in modern AJ in circles. The purple and teal lines show the expected counts (STAR Methods and Data S1, section 12) as predicted by models having a narrower or a longer bottleneck, respectively (Figure 4D; Table S5, models (E) and (F)), as compared to the model inferred using modern IBD. These models, in particular the narrower bottleneck model, do not fit the modern data well. (B) We plot the inferred number of IBD segments in MAJ and ROH segments in EAJ in green and black circles, respectively. The expectations based on the single-population joint-likelihood model, as described in Table S5, model (G), are shown in red and pink lines, respectively. The expected number of short IBD segments in MAJ is overestimated by the model. (C) Simulations for the expected number of AJ founder alleles in EAJ under modern allele frequencies. In each iteration and for each founder SNP, we drew a minor allele count as a binomial variable with n equals to the number of (pseudo-haploid) EAJ individuals that were genotyped in that SNP and p equals the allele frequency in MAJ (from gnomAD (Karczewski et al., 2020)). The figure shows the distribution of the simulated total number of founder SNPs with at least one minor allele across 10,000 runs. We show the 2.5- and 97.5-percentiles in black vertical lines, and the observed number in EAJ in a red line.
